# Identification of differentially methylated region (DMR) networks associated with progression of nonalcoholic fatty liver disease

**DOI:** 10.1038/s41598-018-31886-5

**Published:** 2018-09-11

**Authors:** Kikuko Hotta, Aya Kitamoto, Takuya Kitamoto, Yuji Ogawa, Yasushi Honda, Takaomi Kessoku, Masato Yoneda, Kento Imajo, Wataru Tomeno, Satoru Saito, Atsushi Nakajima

**Affiliations:** 1 Department of Medical Innovation, Osaka University Hospital, 2-2 Yamadaoka, Suita Osaka, 565-0871 Japan; 2 Advanced Research Facilities and Services, Hamamatsu University School of Medicine, 1-20-1 Handayama, Higashi-ku, Hamamatsu Shizuoka, 431-3192 Japan; 3 Department of Gastroenterology and Hepatology, Yokohama City University Graduate School of Medicine, 3-9 Fukuura, Kanazawa-ku, Yokohama, Kanagawa 236-0004 Japan; 4 Department of Gastroenterology, International University of Health and Welfare Atami Hospital, 13-1 Higashi Kaigancho, Atami, Shizuoka 413-0012 Japan

## Abstract

The progression of nonalcoholic fatty liver disease (NAFLD) is affected by epigenetics. We performed differentially methylated region (DMR) and co-methylation analyses to identify DMR networks associated with the progression of NAFLD. DMRs displaying differences in multiple consecutive differentially methylated CpGs between mild and advanced NAFLD were extracted. The average values of topological overlap measures for the CpG matrix combining two different DMRs were calculated and two DMR networks that strongly correlated with the stages of fibrosis were identified. The annotated genes of one network included genes involved in transcriptional regulation, cytoskeleton organization, and cellular proliferation. The annotated genes of the second network were primarily associated with metabolic pathways. The CpG methylation levels in these networks were strongly affected by age and fasting plasma glucose levels, which may be important co-regulatory factors. The methylation status of five DMRs in the second network was reversible following weight loss. Our results suggest that CpG methylation in DMR networks is regulated concomitantly via aging and hyperglycemia and plays important roles in hepatic metabolic dysfunction, fibrosis, and potential tumorigenesis, which occur during the progression of NAFLD. By controlling weight and blood glucose levels, the methylation of DMRs in the second network may be reduced.

## Introduction

Nonalcoholic fatty liver disease (NAFLD) includes a wide spectrum of liver diseases ranging from non-alcoholic fatty liver, which is a benign and non-progressive condition, to nonalcoholic steatohepatitis, which can progress to liver cirrhosis and hepatocellular carcinoma^1,2^. NAFLD has become a common disorder associated with metabolic syndrome in Japan, similar to other developed countries^3,4^. Considering the rapid increase in the number of hepatocellular carcinoma cases resulting from NAFLD, the investigation of NAFLD pathogenesis and the discovery of novel treatment targets are of utmost concern.

Recent progress in experimental and analytical techniques has provided novel insights into the genetic and epigenetic background underlying NAFLD development and progression^5,6^. We previously reported that the patatin-like phospholipase domain containing 3 (*PNPLA3*) rs738409 single nucleotide polymorphism (SNP) is the most predictive and effective variant for a Japanese NAFLD cohort since its risk allele frequency is much higher in the Japanese population compared with that in Caucasian and African populations^7–9^. Using targeted next-generation sequencing, we constructed a high-resolution linkage disequilibrium matrix including rs738409 and reported that variants in SAMM50 sorting and assembly machinery component (*SAMM50*) and parvin β (*PARVB*) are also associated with the development and progression of NAFLD^9^. Moreover, we performed targeted bisulfite sequencing and reported that CpG26 in the regulatory region of *PARVB* was markedly hypomethylated in the livers of patients with NAFLD with advanced fibrosis^10^. Conversely, the hypermethylation of CpG99 in the regulatory region of *PNPLA3* and the subsequent suppression of *PNPLA3* mRNA levels were observed in the livers of patients with NAFLD in advanced stages of fibrosis. To evaluate the epigenetic status of the NAFLD liver, we performed whole hepatic mRNA-sequencing followed by weighted gene co-expression network analysis (WGCNA). We identified two core gene networks involved in NAFLD progression: one contained a scale-free network with four hub genes associated with increases in fibrosis and tumorigenesis, and the other consisted of a random network associated with mitochondrial dysfunction^11^.

DNA methylation analysis represents one of the most important methods for evaluating the epigenetic status in the liver in NAFLD. We performed genome-wide hepatic DNA methylation analysis and a subsequent co-methylation analysis and identified three modules containing individual differentially methylated CpG sites^12^. Our co-methylation analysis demonstrated that one of the modules was associated with an increase in the immune response, while another was associated with mitochondrial dysfunction, impaired lipid metabolism, and reduced oxidoreductase activity in advanced NAFLD. Differentially methylated regions (DMRs), which comprise multiple consecutive methylated CpG sites, have important implications for disease development and progression compared with that of single CpG sites. We extracted 610 DMRs from Japanese patients with mild and advanced NAFLD and found comparable results from an American NAFLD group. DMR analyses indicated that NAFLD livers exhibit increased mitochondrial dysfunction, a decrease in lipid metabolism, impaired oxidoreductase activity, and that they acquire tumorigenic potential^12^.

Our previous RNA sequencing and DNA methylation analysis indicated that the progression of NAFLD arises through coordinated regulatory mechanisms that affect hepatic DNA methylation and the subsequent gene expression. Therefore, in the present study, we applied network modeling to the DMRs identified in mild and advanced NAFLD livers in a Japanese population to investigate the systematic regulatory DMR networks associated with disease progression.

## Results

### Co-methylation analysis of DMRs between mild and advanced NAFLD livers

**Figure 1:**
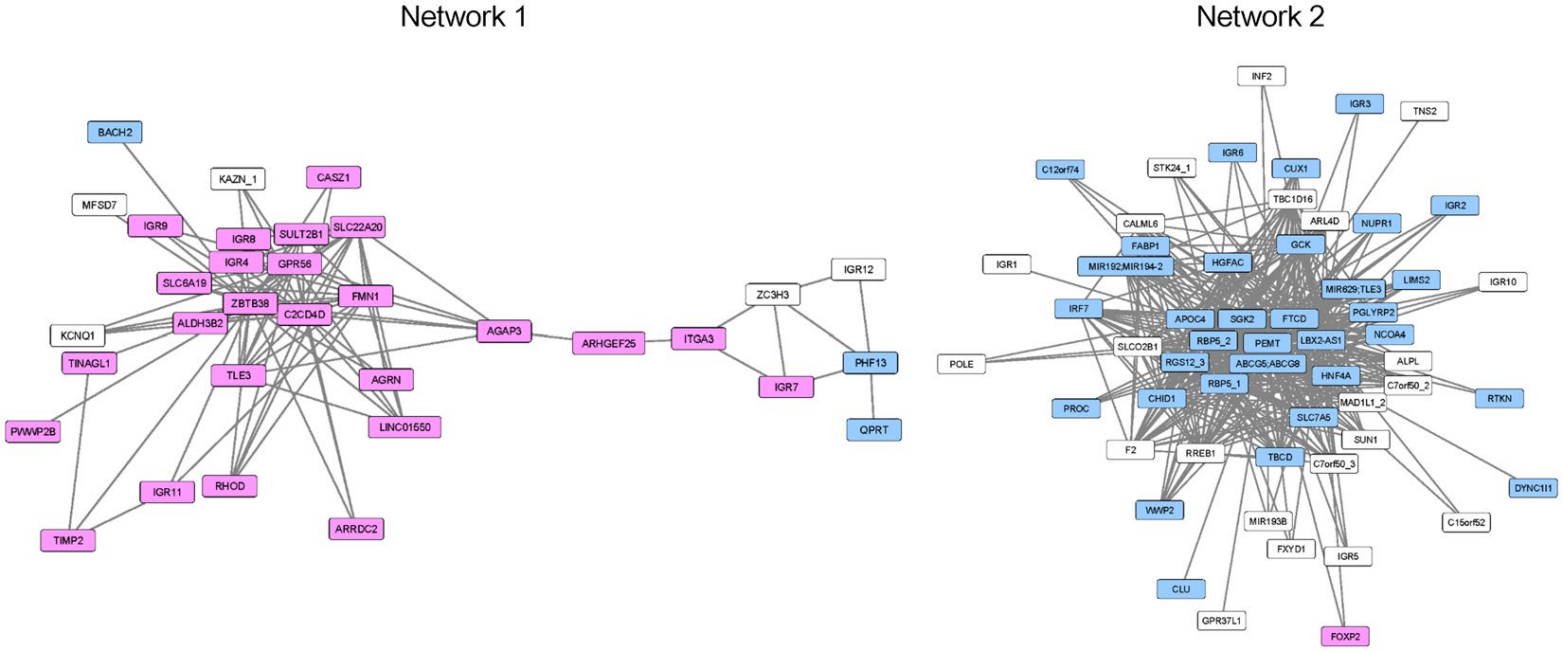
DMR networks in the Japanese NAFLD cohort. The DMR networks (1 and 2) of the Japanese NAFLD cohort were extracted as described in the Methods section. Nodes (DMRs in this case) identified in the Japanese and American NAFLD groups and control livers are indicated in pink. Nodes identified in the Japanese and American NAFLD groups, but not in control livers, are indicated in blue.

Compared with single CpG sites, DMRs are more highly associated with disease^13^. We have used the Probe Lasso method^14^ to investigate DMRs between mild and advanced NAFLD using two populations (Japanese and American) and have identified 610 DMRs consisting of 3,683 CpGs^12^. In this study, we used WGCNA and performed a co-methylation analysis of 3,683 CpGs in 610 DMRs to investigate the DMR networks associated with NAFLD progression in a Japanese cohort. A heatmap of topological overlap measures for 3,683 CpGs suggests that some CpGs in the 610 DMRs may form networks (Supplementary Fig. 1 A). A co-methylation analysis was performed for a combination of single CpGs but this analysis did not take DMRs into account. Thus, to evaluate the co-methylation interconnectedness between DMRs, we calculated the average values of topological overlap measures for the CpG matrix combining two different DMRs, as described in Supplementary Fig. 1B. Then, we constructed a heatmap using the average values of topological overlap measures, which indicated that the 610 DMRs were clustered into at least two networks (Supplementary Fig. 2). To extract a strong co-methylation interconnectedness between DMRs, the cutoff value of the average value of topological overlap measures between two different DMRs was set to 0.15. Two DMR networks were identified in the Japanese NAFLD cohort (Fig. 1). To confirm the reproducibility of these DMR networks, we performed co-methylation analysis and calculated the average values of topological overlap measures for the CpG matrix combining two different DMRs using the same 3,683 CpGs in the American NAFLD dataset (GSE31803)^15^. The heatmap of the average values of topological overlap measures for the American NAFLD dataset was similar to that of the Japanese set and we were able to extract two networks (Supplementary Figs 2 and 3). Many nodes (DMRs in this case) were commonly observed in the two NAFLD groups (the common nodes were indicated in pink or blue in Fig. 1 and Supplementary Fig. 3, respectively). A total of 62 DMRs, 28 DMRs in network 1 and 34 DMRs in network 2, were observed in both NAFLD groups, suggesting that the methylation levels of these DMRs are under a coordinated regulation and play important roles in the progression of NAFLD.

**Figure 2:**
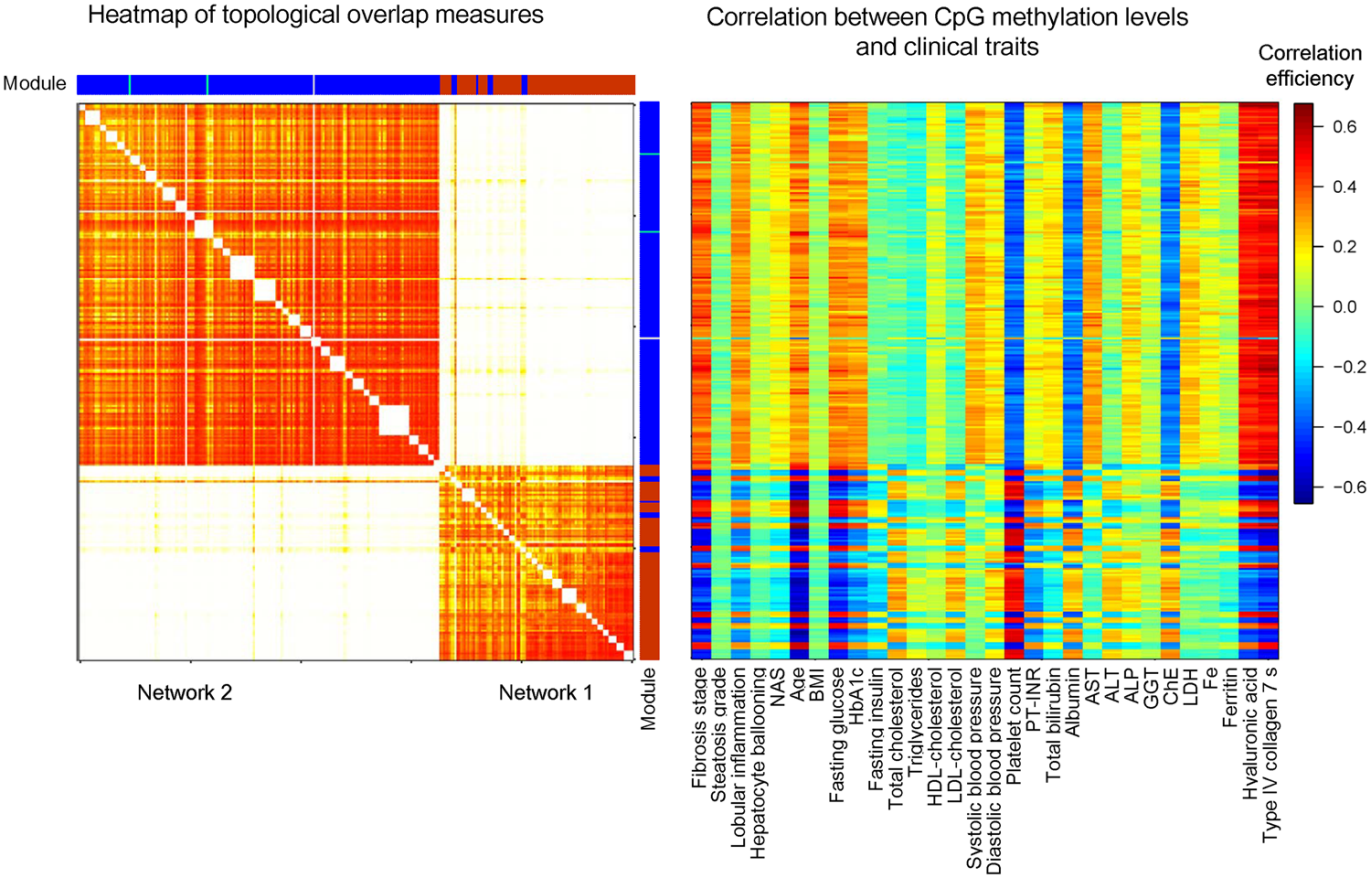
Heatmap of CpGs and correlations between CpG methylation levels and clinical traits in the Japanese NAFLD cohort. Left panel, heatmap of 294 CpGs comprising two DMR networks. The heatmap was constructed using topological overlap measures, a measure of co-methylation interconnectedness between CpGs in the Japanese data. The order of CpGs was sorted by DMRs as presented in Supplementary Table 2. The topological overlap measures of CpGs present in the same DMRs were not used for generating the heatmap (white blocks). Light colors represent low topological overlap; a progressively darker red color indicates increasing overlap. Blocks color-coded in turquoise, blue, brown, yellow, and grey along the diagonal correspond to CpG modules. Right panel, the correlation efficiencies between the methylation levels of CpG sites and clinical traits. Each line corresponds to one CpG site in the heatmap on the left. BMI, body mass index; HbA1c, hemoglobin A1c; HDL, high-density lipoprotein; LDL, low-density lipoprotein; PT-INR, international normalized ratio of prothrombin time; AST, aspartate aminotransferase; ALT, alanine aminotransferase; ALP, alkaline phosphatase; GGT, γ-glutamyl transpeptidase; ChE, cholinesterase; LDH, lactate dehydrogenase; NAS, NAFLD activity score.

We used the average values of topological overlap measures of the CpG matrix combining two different DMRs to extract DMR networks. To verify the validity of this method, the values of topological overlap measures and assigned module colors of 294 CpGs that existed in the 62 DMRs and were observed in both the Japanese and American NAFLD were extracted from the data of the 3,683 CpG sites of 610 DMRs first analyzed by WGCNA. The order of the CpGs was sorted by DMRs and is shown in Supplementary Table 2. We then constructed a heatmap using topological overlap measures of the 294 CpGs. Those CpGs also were clustered into two networks in both the Japanese (Fig. 2) and American NAFLD (Supplementary Fig. 4). Most of the CpGs in network 1 were assigned to the brown module and those in network 2 were assigned to the blue module, supporting the existence of DMR networks in the NAFLD livers.

### Co-methylation and network analyses in control livers

To investigate whether the two networks identified in the NAFLD livers existed in normal livers, we performed the same analysis using β-values for 3,683 CpGs in the German control samples (GSE48325) for which the hepatocyte inflammation, fibrosis stage, and NAFLD activity scores (NAS) are 0^16^. The heatmap containing the average values of topological overlap measures indicated that the genomic regions identified as DMRs in the NAFLD livers clustered into two networks; although the co-methylation interconnectedness between genomic regions in network 2 was weak (Supplementary Fig. 2). Network 1, but not network 2, was identified with a cut-off value of 0.15 in the control liver (Supplementary Fig. 3). The heatmap of topological overlap measures for the 294 CpGs also showed a strong co-methylation interconnectedness between CpGs corresponding to network 1 (Supplementary Fig. 4), similar to that observed in NAFLD patients. The 25th, 50th, and 75th percentiles of the topological overlap measures of network 1 (103 CpGs) were 0.08, 0.12, and 0.16, respectively, in the Japanese NAFLD; 0.06, 0.11, and 0.16, respectively, in the American NAFLD; and 0.06, 0.11, and 0.17, respectively, in the Germany control livers. The 25th, 50th, and 75th percentiles of the topological overlap measures of network 2 (191 CpGs) were 0.11, 0.15, and 0.19, respectively, in the Japanese NAFLD; 0.05, 0.11, and 0.19, respectively, in the American NAFLD; and 0.01, 0.05, and 0.10, respectively, in the Germany control livers. The interconnectedness between CpGs corresponding to network 2 was weak in the control group as compared to that of the NAFLD patients and did not form a clear network 2 in the control group. Most of the CpGs in network 1 were assigned to the brown module in the control group, similar to that observed in the NAFLD patients.

### Genes annotated to DMRs in networks 1 and 2 

A total of 25 nodes (DMRs) forming network 1 in NAFLD livers were also identified in the control livers (Fig. 1 and Supplementary Fig. 3). Among the 25 DMRs, 20 DMRs mapped to known genes and five were located to intergenic regions (Table 1). The annotated genes included genes involved in transcriptional regulation, such as zinc finger and BTB domain containing 38 (*ZBTB38*), transducin like enhancer of split 3 (*TLE3*), and castor zinc finger 1 (*CASZ1*)^17–19^. Moreover, DMRs also mapped to genes involved in cytoskeleton organization, including actin dynamics, and cellular proliferation. These included formin 1 (*FMN1*)^20^, ras homolog family member D (*RHOD*)^21^, and p63RhoGEF, which is encoded by rho guanine nucleotide exchange factor 25 (*ARHGEF25*)^22^. Other genes that overlapped with methylated DMRs were the G protein-coupled receptor 56 (*GPR56*)/adhesion G protein-coupled receptor G1 (*ADGRG1*), which are involved in the RhoA signaling pathway^23^, *ARHGEF25*, and *CASZ1*. In addition, tissue inhibitor of TIMP metallopeptidase inhibitor 2 (*TIMP2*), which interacts with integrin subunit α3β1 to inhibit cellular proliferation^24^ and the integrin subunit α3 (*ITGA3*) genes were also observed in network 1, as was the oncogene agrin (*AGRN*), which enhances cellular proliferation, migration, and oncogenic signaling^25^. Other annotated genes were related to metabolic pathways such as C2 calcium dependent domain containing 4D (*C2CD4D*), solute carrier family 22 member 20 (*SLC22A20*), solute carrier family 6 member 19 (*SLC6A19*), ArfGAP with GTPase domain, ankyrin repeat and PH domain 3 (*AGAP3*), sulfotransferase family 2B member 1 (*SULT2B1*), and aldehyde dehydrogenase 3 family member B2 (*ALDH3B2*).

**Table 1:** Characteristics of common DMRs in network 1.

Genes annotated to DMRs	Degree (the number of edges)	Correlation with methylation levels					Methylation levels in advanced NAFLD	Putative gene function
	NAFLD		Fibrosis stages	Age	Plasma glucose	mRNA levels		
	Japanese	American						
*ZBTB38* (zinc finger and BTB domain containing 38)	25	23	−	−	−	−	hypomethylated	Transcriptional regulator binding to methylated CpG dinucleotides.
*C2CD4D* (C2 calcium dependent domain containing 4D)	19	22	+	+	+	NA	hypermethylated	Calcium-dependent phospholipid binding
*GPR56* (G protein-coupled receptor 56)	17	20	−	−	−	−	hypomethylated	Receptor involved in cell adhesion and probably in cell-cell interactions.
*FMN1* (formin 1)	15	5	−	−	−	−	hypomethylated	Plays a role in the formation of adherens junctions and the polymerization of linear actin cables.
*SLC22A20* (solute carrier family 22 member 20)	13	6	+	+	+	NA	hypermethylated	Organic anion transporter that mediates the uptake of estrone sulfate.
*TLE3* (transducin like enhancer of split 3)	9	20	−	−	−	−	hypomethylated	Transcriptional corepressor that binds to a number of transcription factors. Inhibits the transcriptional activation mediated by catenin β1 (*CTNNB1*) and transcription factor (TCF) family members in Wnt signaling.
*AGAP3* (ArfGAP with GTPase domain, ankyrin repeat and PH domain 3)	7	6	+	+	+	−	hypermethylated	GTPase activity is stimulated by oxidative stress. GTPase which may be involved in the degradation of expanded polyglutamine proteins through the ubiquitin-proteasome pathway.
*SULT2B1* (sulfotransferase family 2B member 1)	6	6	−	−	−	NA	hypomethylated	Sulfotransferase that utilizes 3-phospho-5-adenylyl sulfate as a sulfonate donor to catalyze the sulfate conjugation of cholesterol, hormones, neurotransmitters, drugs and xenobiotic compounds, increasing water solubility and renal excretion.
*LINC01550* (long intergenic non-protein coding RNA 1550)	6	5	−	−	−	−	hypomethylated	Unknown
*ALDH3B2* (aldehyde dehydrogenase 3 family member B2)	5	5	−	−	−	NA	hypomethylated	An aldehyde + NAD(P)^+^ + H_2_O = a carboxylate + NAD(P)H. Oxidizes medium and long chain aldehydes into non-toxic fatty acids.
*RHOD* (ras homolog family member D)	5	3	+	+	+	NS	hypermethylated	Involved in endosome dynamics. Involved in the internalization and trafficking of activated tyrosine kinase receptors. Participates in the reorganization of the actin cytoskeleton.
*SLC6A19* (solute carrier family 6 member 19)	4	5	−	−	−	−	hypomethylated	Transporter that mediates resorption of neutral amino acids across the apical membrane of renal and intestinal epithelial cells. This uptake is sodium-dependent and chloride-independent.
*ARRDC2* (arrestin domain containing 2)	3	5	−	−	−	−	hypomethylated	Unknown
*TIMP2* (TIMP metallopeptidase inhibitor 2)	3	3	−	−	−	−	hypomethylated	TIMP-2 binds to integrin α3β1. TIMP-2 induces gene expression, to promote G1 cell cycle arrest, and to inhibit cell migration
*ITGA3* (integrin subunit α 3)	3	8	+	+	+	+	hypermethylated	Integrin α3/β1 is a receptor for fibronectin, laminin, collagen, epiligrin, thrombospondin and chondroitin sulfate proteoglycan 4. May participate in the adhesion, formation of invadopodia and matrix degradation processes, promoting cell invasion.
*AGRN* (agrin)	3	3	+	+	+	+	hypermethylated	Acetylcholine receptor aggregating factor, agrin, component of the synaptic basal lamina on the surface of muscle fibers. Enhances cellular proliferation, migration and oncogenic signaling. Regulate Arp2/3-dependent ruffling, invadopodia formation and the epithelial–mesenchymal transition through sustained focal adhesion integrity that drives liver tumorigenesis.
*ARHGEF25* (rho guanine nucleotide exchange factor 25)	2	2	+	+	+	NS	hypermethylated	May play a role in the reorganization of the actin cytoskeleton in different tissues. Functions as a guanine nucleotide exchange factor for the Rho family of small GTPases. Specifically links G α q/11-coupled receptors to RHOA activation. May be an important regulator of processes involved in axon and dendrite formation.
*TINAGL1* (tubulointerstitial nephritis antigen like 1)	2	6	−	−	−	NS	hypomethylated	May be implicated in the adrenocortical zonation and in mechanisms for repressing *CYP11B1* gene expression in adrenocortical cells.
*CASZ1* (castor zinc finger 1)	2	5	−	−	−	−	hypomethylated	Transcriptional activator. Involved in vascular assembly and morphogenesis through direct transcriptional regulation of EGFL7
*PWWP2B* (PWWP domain containing 2B)	1	6	−	−	−	−	hypomethylated	Unknown

NA: not available; NS: not significant.

Network 2 was observed only in NAFLD livers. Three DMRs were located in intergenic regions and 31 DMRs were annotated to genes that function in the lipid metabolism, the immune response, and transcription regulation (Table 2). The most meaningful gene annotated to DMRs was phosphatidylethanolamine N-methyltransferase (*PEMT*), which exhibited a high degree (number of edges connected to a given DMRs) and plays important roles in choline metabolism^26^. *PEMT*-deficient mice develop steatohepatitis in the presence of a choline-deficient diet. Among other genes that mapped to DMRs in network 2, the ATP binding cassette subfamily G member 5 (*ABCG5*) and 8 (*ABCG8*) form heterodimers and extrude sterols, especially xenosterols, from the liver into the bile^27^. Furthermore, the HGF activator (*HGFAC*), hepatocyte nuclear factor 4 α (*HNF4A*), and SGK2, Serine/Threonine Kinase 2 (*SGK2*) are targets of hepatocyte nuclear factor 1 α (HNF1A)^28^, while miR-194/192 (*MIR194-2*/*MIR192*) is the target of HNF4A^29^.

**Table 2:** Characteristics of common DMRs in network 2.

Genes annotated to DMRs	Degree (the number of edges)		Correlation with methylation levels				Methylation levels in advanced NAFLD	Putative gene function
	NAFLD		Fibrosis stages	Age	Plasma glucose	mRNA levels		
	Japanese	American						
*PEMT* (phosphatidylethanolamine N-methyltransferase)	55	38	+	+	+	NS	hypermethylated	Phosphatidylethanolamine N-methyltransferase, converting phosphatidylethanolamine to phosphatidylcholine, involved in hepatocyte proliferation and liver cancer.
*LBX2-AS1* (LBX2 antisense RNA 1)	49	1	+	+	+	NS	hypermethylated	Unknown
*RBP5* (2nd DMR) (retinol binding protein 5)	44	1	+	+	+	−	hypermethylated	Intracellular transport of retinol.
*FTCD* (formimidoyltransferase cyclodeaminase)	39	5	+	+	+	−	hypermethylated	Folate-dependent enzyme, that displays both transferase and deaminase activity. Channels one-carbon units from formiminoglutamate to the folate pool.
*ABCG5;ABCG8* (ATP binding cassette subfamily G member 5; ATP binding cassette subfamily G member 8)	35	8	+	+	+	−	hypermethylated	Transporter that appears to play an indispensable role in the selective transport of the dietary cholesterol in and out of the enterocytes and in the selective sterol excretion by the liver into the bile.
*SGK2* (SGK2, serine/threonine kinase 2)	34	16	+	+	+	+	hypermethylated	Serine/threonine-protein kinase involved in the regulation of a wide variety of ion channels, membrane transporters, cell growth, survival, and proliferation.
*RBP5* (1st DMR) (retinol binding protein 5)	34	2	+	+	+	−	hypermethylated	Intracellular transport of retinol.
*APOC4* (apolipoprotein C4)	31	5	+	+	+	−	hypermethylated	May participate in lipoprotein metabolism.
*RGS12* (3rd DMR) (regulator of G protein signaling 12)	30	1	+	+	+	−	hypermethylated	Regulates G protein-coupled receptor signaling cascades. Inhibits signal transduction by increasing the GTPase activity of G protein α subunits, thereby driving them into their inactive GDP-bound form. Behaves as a cell cycle-dependent transcriptional repressor, promoting inhibition of S-phase DNA synthesis.
*CHID1* (chitinase domain containing 1)	29	1	+	+	+	−	hypermethylated	Saccharide- and LPS-binding protein with possible roles in pathogen sensing and endotoxin neutralization.
*HGFAC* (HGF activator)	26	1	+	+	+	NS	hypermethylated	Activates hepatocyte growth factor (HGF) by converting it from a single chain to a heterodimeric form.
*GCK* (glucokinase)	23	1	+	+	+	NS	hypermethylated	Glucokinase
*PGLYRP2* (peptidoglycan recognition protein 2)	19	9	+	+	+	NS	hypermethylated	May play a scavenger role by digesting biologically active peptidoglycan into biologically inactive fragments
*TBCD* (tubulin folding cofactor D)	19	2	+	+	+	−	hypermethylated	Tubulin-folding protein implicated in the first step of the tubulin folding pathway and required for tubulin complex assembly.
*SLC7A5* (solute carrier family 7 member 5)	18	1	+	+	+	NS	hypermethylated	Sodium-independent, high-affinity transport of large neutral amino acids such as phenylalanine, tyrosine, leucine, arginine, and tryptophan.
*IRF7* (interferon regulatory factor 7)	18	1	+	+	+	NS	hypermethylated	Key transcriptional regulator of type I interferon-dependent immune responses: plays a critical role in the innate immune response against DNA and RNA viruses.
*MIR192;MIR194-2* (microRNA 192; microRNA 194-2)	17	7	+	+	+	NA	hypermethylated	MicroRNAs in cancer.
*HNF4A* (hepatocyte nuclear factor 4 α)	17	3	+	+	+	−	hypermethylated	Transcriptionally controlled transcription factor. Binds to DNA sites required for the transcription of alpha 1-antitrypsin, apolipoprotein CIII, transthyretin genes and HNF1-α
*MIR629;TLE3* (microRNA 629; tansducin like enhancer of split 3)	9	10	+	+	+	NA; +	hypermethylated	Transcriptional corepressor that binds to a number of transcription factors. Inhibits the transcriptional activation mediated by CTNNB1 and TCF family members in Wnt signaling.
*NCOA4* (nuclear receptor coactivator 4)	9	4	+	+	+	NS	hypermethylated	Enhances the androgen receptor transcriptional activity in prostate cancer cells. Ligand-independent coactivator of the peroxisome proliferator-activated receptor γ
*LIMS2* (LIM zinc finger domain containing 2)	8	1	+	+	+	NS	hypermethylated	Adapter protein in a cytoplasmic complex linking β-integrins to the actin cytoskeleton, bridges the complex to cell surface receptor tyrosine kinases and growth factor receptors. Plays a role in modulating cell spreading and migration.
*FABP1* (fatty acid binding protein 1)	8	2	+	+	+	NS	hypermethylated	Plays a role in lipoprotein-mediated cholesterol uptake in hepatocytes. Binds cholesterol, free fatty acids and their coenzyme A derivatives, bilirubin, and other small molecules in the cytoplasm.
*WWP2* (WW domain containing E3 ubiquitin protein ligase 2)	8	2	+	+	+	NS	hypermethylated	E3 ubiquitin-protein ligase which accepts ubiquitin from an E2 ubiquitin-conjugating enzyme in the form of a thioester and then directly transfers ubiquitin to targeted substrates.
*NUPR1* (nuclear protein 1)	7	1	+	+	+	NS	hypermethylated	Chromatin-binding protein that converts stress signals into a program of gene expression that results in cellular resistance to stress induced by a change in the microenvironment. Interacts with MSL1 and inhibits histone H4 Lys-16 acetylation (H4K16ac).
*CUX1*(cut like homeobox 1)	7	1	+	+	+	NS	hypermethylated	Probably has a broad role in mammalian development as a repressor of developmentally regulated gene expression.
*PROC* (protein C, inactivator of coagulation factors Va and VIIIa)	4	1	+	+	+	−	hypermethylated	Protein C is a vitamin K-dependent serine protease that regulates blood coagulation by inactivating factors Va and VIIIa in the presence of calcium ions and phospholipids.
*C12orf74* (chromosome 12 open reading frame 74)	3	1	+	+	+	NA	hypermethylated	Unknown
*RTKN*(rhotekin)	2	1	+	+	+	−	hypermethylated	Mediates Rho signaling to activate NF-κB and may confer increased resistance apoptosis for cells in gastric tumors.
*FOXP2* (forkhead box P2)	2	3	+	+	+	−	hypermethylated	Transcriptional repressor that may play a role in the specification and differentiation of the lung epithelium. May also play a role in developing neural, gastrointestinal and cardiovascular tissues.
*CLU* (clusterin)	1	2	+	+	+	−	hypermethylated	Functions as an extracellular chaperone that prevents aggregation of nonnative proteins. Secreted isoform 1 protects cells against apoptosis and cytolysis by the complement system. Intracellular isoforms interact with ubiquitin and SKP1-CUL1-F-box protein E3 ubiquitin-protein ligase complexes.
*DYNC1I1* (dynein cytoplasmic 1 intermediate chain 1)	1	1	+	+	+	NS	hypermethylated	Acts as one of several non-catalytic accessory components of the cytoplasmic dynein 1 complex that are thought to be involved in linking dynein to cargos and adapter proteins that regulate dynein function.

NA: not available; NS: not significant.

### Relationship between CpG methylation levels in DMRs and clinical traits

The β-values of each CpG in the mild and advanced NAFLD group and the control group are provided in Supplementary Table 2. Among the 25 DMRs in network 1, the methylation levels of CpGs in 17 DMRs were negatively correlated with the stages of fibrosis, age, fasting plasma glucose and hyaluronic acid levels, and Type IV collagen 7S and positively correlated to platelet count (Fig. 2, Table 1, and Supplementary Table 3). The remaining eight DMRs in network 1 showed inverted profiles. Information regarding the age and BMI were available for the control group. The methylation levels of CpGs in the above-mentioned 17 DMRs were negatively correlated with age, while those in the eight DMRs were positively correlated, as observed in the Japanese NAFLD cohort (Supplementary Fig. 4).

In contrast to the CpGs in network 1, the methylation levels of all the CpGs in network 2 were positively correlated with the stages of fibrosis, age, fasting plasma glucose and hyaluronic acid levels, and Type IV collagen 7S and negatively correlated to platelet count in the Japanese NAFLD livers.

### Relationship between CpG methylation levels in DMRs and gene expression levels

The relationship between the mRNA and CpG methylation levels was investigated in 56 Japanese and 44 American NAFLD livers. In network 1, the expression levels of 16 genes annotated to DMRs were available. The methylation levels of CpGs in 11 DMRs were negatively and those in two DMRs were positively correlated with the expression of their annotated genes (Table 1 and Supplementary Table 4). For three DMRs, no significant correlations were observed. In network 2, the expression levels of 29 genes annotated to DMRs were available. The methylation levels of CpGs in 13 DMRs were negatively and those in two DMRs were positively correlated with the expression of their annotated genes, while for 14 DMRs, no significant correlations were observed (Table 2 and Supplementary Table 4).

## Discussion

By introducing the average values of topological overlap measures into DMR and co-methylation analyses, we could identify two DMR networks associated with NAFLD progression. The methylation levels of CpGs in these DMR networks may be under coordinated regulation. Indeed, the genes annotated to DMRs in the two networks included genes involved in known functional pathways: *GPR56*/*ADGRG1*, *ARHGEF25* and *CASZ1*, *TIMP2* and *ITGA3*, and *SGK2*, *HNF1A* and *MIR194-2*/*MIR192*. These results would support the existence of these DMR networks in NAFLD livers.

**Figure 3:**
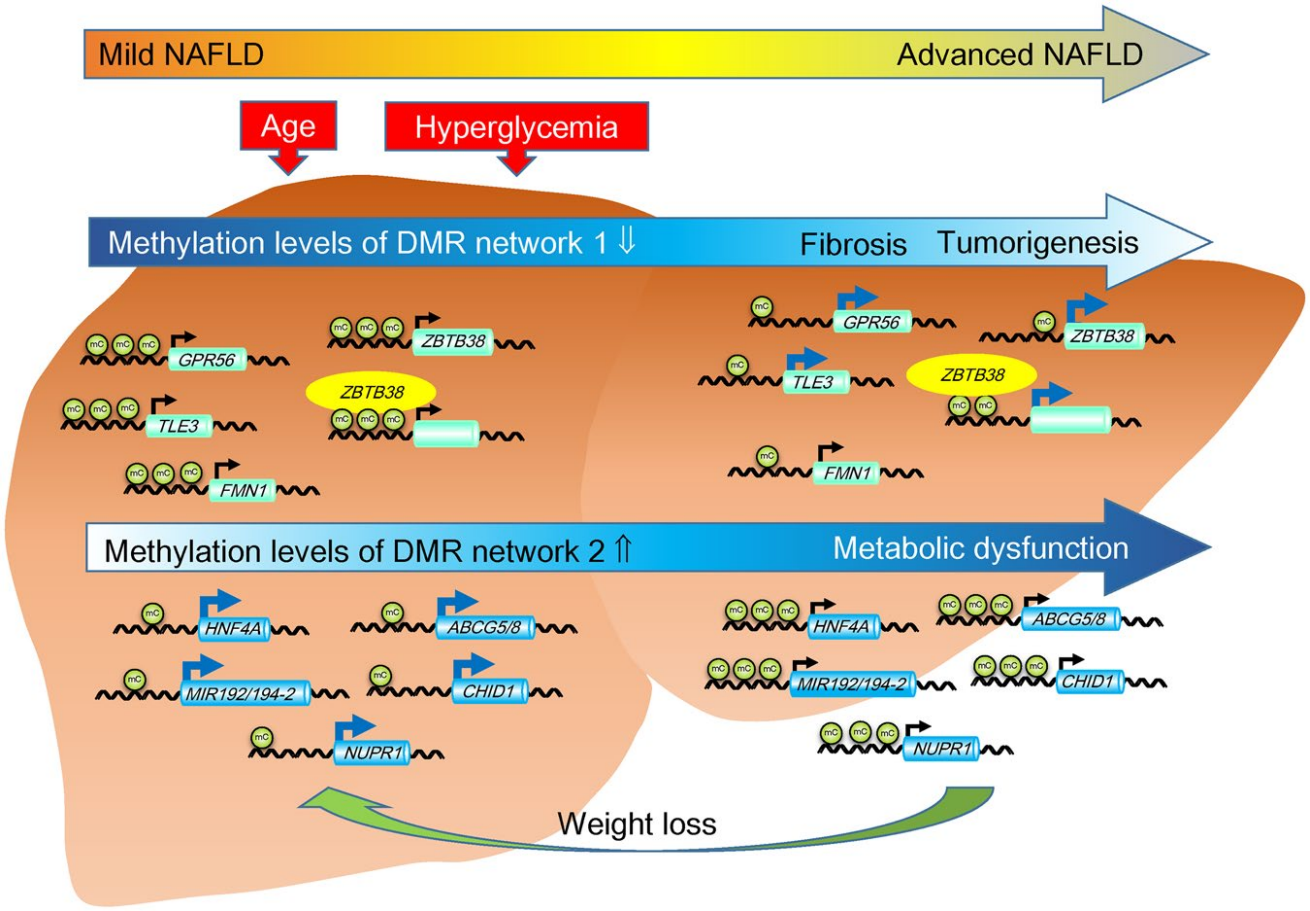
Potential mechanisms for the progression of nonalcoholic fatty liver disease (NAFLD) based on DMR networks.

The methylation levels of CpGs in two DMR networks were strongly correlated to the stages of fibrosis and serum markers (platelet count, hyaluronic acid, and Type IV collagen 7S). These results suggested an impaired coordinated regulation of methylation in these networks is occurring as NAFLD progresses. We were especially interested in the coordinated regulating factors in these DMR networks. Age was strongly correlated with CpG methylation levels in these networks in the Japanese NAFLD cohort. Aging affects DNA methylation, possibly through the cumulative effect of various environmental factors^30^. Therefore, aging may represent one of the factors undergoing a coordinated regulation in DMR networks, which leads to the progression of NAFLD. Another important factor is the blood glucose level, which was also shown to be correlated with methylation. Patients with NAFLD are often affected by obesity and type 2 diabetes. Considering the effect of aging, long-term hyperglycemia induced by excess calorie-intake may cause the coordinated change in methylation levels in DMR networks, resulting in the progression of NAFLD (Fig. 3).

One interesting gene annotated to DMRs in network 1 was *ZBTB38*, which exhibited a high degree of connection to DMRs based on the number of edges. ZBTB38 is a transcriptional regulator that binds directly to methylated CpG dinucleotides^31^. *ZBTB38* methylation levels decreased as fibrosis progressed and were inversely correlated with its expression. Therefore, increased expression of the *ZBTB38* may play an important role in regulating transcriptional levels in NAFLD livers. Another interesting candidate is *GPR56*/*ADGRG1*, which encodes a member of the G protein-coupled receptor family. The product of *GPR56*/*ADGRG1* is involved in the RhoA^23^ and NF-κB-signaling pathways^32^ and plays important roles in tissue development and cell proliferation. Other genes annotated to DMRs in network 1 included genes involved in transcriptional regulation (*TLE3* and *CASZ1*), cytoskeleton organization (*FMN1*, *RHOD*, and *ARHGEF25*), the RhoA signaling pathway (*ARHGEF25* and *CASZ1*), and cellular proliferation (*TIMP2*, and *ITGA3*). The expression levels of *GPR56*/*ADGRG1*, *TLE3*, *CASZ1*, *FMN1*, *TIMP2*, and *ITGA3* increased with the progression of fibrosis, suggesting that the NAFLD liver undergoes fibrosis and acquires tumorigenic potential through coordinated changes in DNA methylation and the subsequent changes in gene expression levels in network 1.

Many of the genes annotated to the DMRs in network 2 were part of metabolic pathway. The methylation levels of CpGs in network 2 increased with the progression of fibrosis, suggesting that network 2 is associated with the abnormal hepatic metabolism seen in NAFLD. One important gene in DMR network 2 is *PEMT*, which plays important roles in choline metabolism^26^. Moreover, *PEMT*-deficient mice develop steatohepatitis following the administration of a choline-deficient diet. Thus, a long-term high-calorie diet containing unbalanced nutrients may lead to a relative choline-deficient status, resulting in the progression of NAFLD.

Our previous report indicated that seven DMRs are hypermethylated in advanced NAFLD and that their methylation levels decrease in response to weight loss following bariatric surgery^12^. Surprisingly, five of the above seven DMRs were included in DMR network 2 (*HNF4A*, *MIR192*/*MIR194-2*, *ABCG5*/*ABCG8*, chitinase domain-containing 1 [*CHID1*], and nuclear protein 1, transcriptional regulator [*NUPR1*]). The *HNF4A*, *MIR192*/*MIR194-2* and *NUPR1* genes function in transcription regulation. CHID1 binds saccharides and lipopolysaccharides and may play potential roles in pathogen sensing and endotoxin neutralization^33^. ABCG5 and ABCG8 form a sterol transporter to prevent the accumulation of dietary sterols. Therefore, factors that regulate the methylation levels of DMR network 2 would represent good candidates for the treatment of NAFLD. Age and fasting plasma glucose levels represent such factors. Long-term weight control would normalize blood glucose levels, resulting in decreased methylation levels in DMR network 2 (Fig. 3). In contrast to DMR network 2, network 1 did not include DMRs that respond to weight loss. Follow-up biopsies were performed 5–9 months after bariatric surgery^16^. Despite the restoration of the methylation levels in DMR network 2 following prolonged weight loss, the methylation levels in network 1 proved resistant to weight loss.

We identified two networks by investigating the interconnectedness between DMRs, which represent differentially methylated genomic regions between mild and advanced NAFLD. Interestingly, the genomic regions in network 1 also formed a network in the control livers. Therefore, the genomic regions identified as DMRs associated with the progression of NAFLD may be regulated concomitantly before the development of NAFLD. Aging also represented an important factor that affects the methylation level in network 1 in the control liver. Therefore, aging along with other environmental factors, such as excess calorie intake, may cause NAFLD.

We previously reported the existence of two core gene expression networks; a scale-free network with four hub genes and a random network^11^. The genes annotated to the DMR networks did not completely overlap with gene expression networks. One reason for this is that various mechanisms other than DNA methylation are involved in the regulation of gene expression. Due to limitations of techniques used to measure mRNA, it is difficult to accurately reflect genes with high or low expression levels in network analysis. In contrast, methylation level can be uniformly measured in the whole genome, in DMRs in intergenic regions, and in genes with high or low expression levels that constitute the DMR networks. Thus, the networks of DMRs may be differ from gene expression networks. Furthermore, the techniques for measuring levels of DNA methylation are limited by CpG-specific probes and the intensity of fluorescence signal. Indeed, while CpG sites of CpG99 in *PNPLA3* and CpG26 in *PARVB* were shown in our previous study to be differentially methylated between mild and advanced NAFLD^10^, they were not involved in the DMRs in the current study since there was only one probe in CpG26 and the probes in CpG99 exhibited low β-values (<0.05). Therefore, further investigation is necessary to elucidate the integrated networks of gene expression and DNA methylation.

In summary, we identified two DMR networks associated with the progression of NAFLD. The methylation levels of both networks were affected by aging and blood glucose levels. Long-term weight control may normalize blood glucose levels, resulting in decreased methylation levels in DMR network 2 and the recovery of the hepatic metabolic function. The change in methylation levels in network 1 would occur irreversibly with age, leading to fibrosis and tumorigenesis in the NAFLD liver.

## Methods

### DNA methylation analysis

For the DNA methylation analysis, we used our recently published Japanese hepatic DNA methylation data (JGAS00000000059, http://trace.ddbj.nig.ac.jp/jga/index.html)^12^. Genome-wide DNA methylation levels were determined using the Illumina Infinium HumanMethylation450 BeadChip (San Diego, CA, USA). Raw DNA methylation data were preprocessed by normalization to appropriate internal control probes. Probes with intensities indistinguishable from that of the background (detection *P*-value > 0.05) in more than one sample, probes with a bead count <3 in at least 5% of the samples, SNP probes, and cross-reactive probes were excluded. The effects of two types of CpG probes on CpG methylation measurements were corrected using beta-mixture quantile normalization^34^ and batch effects were corrected using a ComBat normalization method^35^ in the R/Bioconductor package ChAMP^36^. After these procedures, 431,736 CpG sites were extracted. The DMRs between mild (fibrosis stages 0–2; n = 35) and advanced NAFLD (fibrosis stages 3–4; n = 25) were identified using the Probe Lasso method^14^. Hepatic DNA methylation data for NAFLD from an American population (NCBI Gene Expression Omnibus accession number GSE31803)^15^, were used for replication analysis. DMRs between mild (n = 33) and advanced (n = 23) NAFLD in GSE31803 were determined using the Probe Lasso method over 431,736 CpG sites.

### Co-methylation analysis of DMRs

A total of 610 DMRs containing 3,683 CpGs across the Japanese and American NAFLD cohorts were obtained as previously reported^12^. Hepatic DNA methylation data for control subjects (n = 34) were obtained from GSE48325 for the German population^16^. To assess the inter-correlation among these CpG sites, a co-methylation analysis was performed using WGCNA R package^37,38^. In this analysis, a network was represented by the adjacency matrix *A* = [*α*_*ij*_], where *α*_*i,j*_ was |*PCC*_*i,j*_|^*β*^, *PCC*_*i,j*_ was the Pearson correlation coefficient between the methylation levels of CpG probes *i* and *j* across samples, and *β* was a soft threshold. The *β* was set at 16 for the Japanese NAFLD, 14 for the American NAFLD, and 14 for the German control, which were the smallest values at which a co-methylation network exhibited scale-free properties with a model-fitting index of *R*^2^ > 0.70 (Supplementary Fig. 5). Topological overlap measures, a measure of co-methylation interconnectedness between probes^39^, were obtained in two NAFLD and control groups. To evaluate the co-methylation interconnectedness between DMRs, we calculated the average values of topological overlap measures of the CpG matrix combining two different DMRs, as described in Supplementary Fig. 1B. The cutoff value of the average value of the topological overlap measures was set to 0.15 and the networks were visualized using Cytoscape^40^.

### Correlation between the methylation levels of CpG and gene expression

The RNA sequencing data of our recently published Japanese NAFLD cohort (n = 56) (JGAS00000000059, http://trace.ddbj.nig.ac.jp/jga/index.html) was used^11^. Sequence reads from each sample were aligned to the reference human genome (UCSC hg19) in STAR 2-pass mapping (https://github.com/alexdobin/STAR)^41^ to align reads after the removal of adaptor sequences using Cutadapt 1.13 (https://cutadapt.readthedocs.io/en/stable/). Reads were counted using HTSeq (https://htseq.readthedocs.io/en/release_0.9.1/)^42^ and the counts per gene were normalized using DESeq 2R/Bioconductor package^43^.

The expression data in 44 NAFLD samples, which were used for DNA methylation analysis in the American cohort, were derived from GSE31803 (Affymetrix Human Genome U133 Plus 2.0 GeneChip arrays)^44^. Quality Assessment and normalization were performed using the R/Bioconductor package limma^45^.

### Clinical traits and other statistical analysis

This study was conducted in accordance with the Declaration of Helsinki and the Japanese ethical guidelines for human genome and gene analysis research. Written informed consent was obtained from each participant and the protocol was approved by the ethics committees at Osaka University (No. 682, Osaka, Japan), and Yokohama City University (A171200002, Yokohama, Japan). The clinical traits of the Japanese NAFLD are presented in Supplementary Table 1. Clinical data were compared using *t* tests between patients with mild or advanced NAFLD. Fisher’s exact test was used to analyze the male to female ratio, as well as the prevalence of type 2 diabetes. The meta-analysis of correlation tests was performed using the metacor R-package. Other data were analyzed in R (http://www.r-project.org/). The codes are indicated in the Supplementary Data.

## Electronic supplementary material

Dataset1
