# Supplementary material for: Identification of differentially methylated region (DMR) networks associated with progression of nonalcoholic fatty liver disease
Source: Sci Rep. 2018 Sep 11;8:13567. doi: 10.1038/s41598-018-31886-5 (PMC6134034; doi:10.1038/s41598-018-31886-5)
Supplement: Supplementary file 1 — Dataset1 [file 41598_2018_31886_MOESM1_ESM.pdf]

Identification of differentially methylated region (DMR) networks associated with progression of nonalcoholic fatty liver disease

Kikuko Hotta[1], Aya Kitamoto[2], Takuya Kitamoto[2], Yuji Ogawa[3], Yasushi Honda[3], Takaomi Kessoku[3], Masato Yoneda[3], Kento Imajo[3], Wataru Tomeno[3,4], Satoru Saito[3] and Atsushi Nakajima[3]

[1]Department of Medical Innovation, Osaka University Hospital, 2-2 Yamadaoka, Suita, Osaka 565-0871, Japan
[2]Advanced Research Facilities and Services, Hamamatsu University School of Medicine, 1-20-1 Handayama, Higashi-ku, Hamamatsu, Shizuoka 431-3192, Japan
[3]Department of Gastroenterology and Hepatology, Yokohama City University Graduate School of Medicine, 3-9 Fukuura, Kanazawa-ku, Yokohama, Kanagawa 236-0004, Japan
[4]Department of Gastroenterology, International University of Health and Welfare Atami Hospital. 13-1 Higashi Kaigancho, Atami, Shizuoka 413-0012, Japan

Supplementary Table 1
Supplementary Table 2
Supplementary Table 3
Supplementary Table 4
Supplementary Fig. 1
Supplementary Fig. 2
Supplementary Fig. 3
Supplementary Fig. 4
Supplementary Fig. 5
Supplementary Methods

Supplementary Table 1. Clinical characteristics of Japanese NAFLD cohort.

	Mild NAFLD (fibrosis stages 0-2)	Advanced NAFLD (fibrosis stages 3-4)	*P*
n	35	25	
Men / women	23 / 12	16 / 9	1.00*
age (year)	47.5 ± 18.0	56.9 ± 13.6	0.024
BMI (kg/m^2)	27.9 ± 4.7	29.1 ± 4.5	0.33
Fasting glucose (mg/dL)	104.4 ± 19.0	114.7 ± 18.2	0.048
HbA1c (%)	5.8 ± 0.8	6.2 ± 1.0	0.12
Fasting insulin (μU/ml)	16.9 ± 22.6	18.0 ± 7.7	0.79
Total cholesterol (mg/dL)	206.7 ± 33.4	189.2 ± 39.1	0.076
Triglycerides (mg/dL)	156.5 ± 79.7	146.8 ± 76.0	0.64
HDL-cholesterol (mg/dL)	51.0 ± 12.1	50.7 ± 18.6	0.94
LDL-cholesterol (mg/dL)	124.3 ± 30.0	109.1 ± 26.0	0.040
Systolic blood pressure (mmHg)	128.7 ± 18.4	131.0 ± 14.6	0.60
Diastolic blood pressure (mmHg)	78.7 ± 15.4	77.2 ± 12.1	0.69
Platelet (×10,000/μL)	22.6 ± 5.2	16.8 ± 5.0	6.5×10^{-5}
PT-INR	1.0 ± 0.2	1.1 ± 0.1	0.046
Total bilirubin (mg/dL)	0.8 ± 0.3	0.9 ± 0.3	0.092
Albumin (g/dL)	4.7 ± 0.3	4.3 ± 0.4	2.0×10^{-3}
AST (IU/L)	43.9 ± 20.8	60.6 ± 24.7	8.6×10^{-3}
ALT (IU/L)	75.1 ± 50.1	80.0 ± 43.4	0.69
ALP (IU/L)	239.2 ± 79.3	264.5 ± 92.5	0.28
GGT (IU/L)	69.1 ± 53.6	85.2 ± 70.5	0.34
ChE (IU/L)	370.8 ± 80.3	322.2 ± 90.8	0.039
LDH (IU/L)	198.8 ± 33.9	215.7 ± 40.6	0.11
Fe (μg/dL)	131.5 ± 41.7	143.5 ± 31.4	0.27
Ferritin (ng/mL)	235.4 ± 157.0	241.4 ± 156.5	0.90
Hyaluronic acid (ng/dL)	36.5 ± 40.5	110.1 ± 93.5	8.8×10^{-4}
Type IV collagen 7S (ng/dL)	4.3 ± 0.8	7.3 ± 2.1	1.5×10^{-7}
Steatosis grade (0-3)	1.6 ± 0.7	1.4 ± 0.7	0.15
Lobular inflammation (0-3)	1.1 ± 0.7	1.5 ± 0.6	0.013
Hepatocyte ballooning (0-2)	0.7 ± 0.7	0.6 ± 0.7	0.80
NAS (0-8)	3.4 ± 1.6	3.5 ± 1.4	0.79
Fibrosis stage (0-4)	1.1 ± 0.7	3.0 ± 0.2	5.2×10^{-18}
Type 2 diabetes (%)	9 (25.7)	12 (48.0)	0.10*

Values represent the means ± SD. Clinical data between mild and advanced NAFLD were compared using a *t*-test, whereas ratios (*) were analyzed using Fisher’s exact test. BMI, body mass index; HbA1c, hemoglobin A1c; HDL, high-density lipoprotein; LDL, low-density lipoprotein; PT-INR, international normalized ratio of prothrombin time; AST, aspartate aminotransferase; ALT, alanine aminotransferase; ALP, alkaline phosphatase; GGT, γ-glutamyl transpeptidase; ChE, cholinesterase; LDH, lactate dehydrogenase; NAS, NAFLD activity score.

Supplementary Table 2. CpG methylation levels (average β-values) in 62 DMRs associated with NAFLD progression.

DMR (node)	Probe	Chr	Position (hg19)	Annotated genes	CpG feature		Average β-value				
							Japanese NAFLD		American NAFLD		Germany
					Location	Island	Mild	Advanced	Mild	Advanced	Control
Network 1											
**ZBTB38**	cg06137072	3	141,087,187	*ZBTB38*	5'UTR	open sea	0.60	0.48	0.69	0.58	0.64
	cg13029400	3	141,087,190	*ZBTB38*	5'UTR	open sea	0.54	0.40	0.67	0.52	0.62
	cg08360599	3	141,087,261	*ZBTB38*	5'UTR	open sea	0.55	0.42	0.56	0.45	0.48
	cg21370924	3	141,087,313	*ZBTB38*	5'UTR	open sea	0.60	0.41	0.62	0.47	0.54
	cg21474062	3	141,087,363	*ZBTB38*	5'UTR	open sea	0.62	0.46	0.63	0.49	0.57
**C2CD4D**	cg15015892	1	151,810,887	*C2CD4D*	Body	island	0.21	0.37	0.16	0.34	0.22
	cg05021743	1	151,810,893	*C2CD4D*	Body	island	0.18	0.34	0.13	0.28	0.19
	cg10781408	1	151,810,899	*C2CD4D*	Body	island	0.19	0.36	0.16	0.33	0.21
	cg04296699	1	151,810,904	*C2CD4D*	Body	island	0.19	0.34	0.17	0.35	0.23
**GPR56**	cg01410801	16	57,653,669	*GPR56*	TSS1500	open sea	0.76	0.63	0.84	0.71	0.82
	cg25645462	16	57,653,702	*GPR56*	TSS1500	open sea	0.77	0.70	0.72	0.65	0.69
	cg00550797	16	57,653,904	*GPR56*	TSS200	open sea	0.80	0.71	0.79	0.69	0.76
	cg16630572	16	57,653,917	*GPR56*	5'UTR	open sea	0.82	0.73	0.80	0.70	0.78
**FMN1**	cg15175581	15	33,360,262	*FMN1*	TSS200	open sea	0.60	0.45	0.57	0.46	0.54
	cg09347959	15	33,360,271	*FMN1*	TSS200	open sea	0.64	0.46	0.60	0.46	0.57
	cg17454592	15	33,360,353	*FMN1*	TSS1500	open sea	0.51	0.38	0.49	0.39	0.46
**SLC22A20**	cg02675946	11	64,993,239	*SLC22A20*	Body	shore	0.15	0.29	0.14	0.23	0.15
	cg27428304	11	64,993,281	*SLC22A20*	Body	island	0.12	0.26	0.12	0.21	0.13
	cg23930334	11	64,993,335	*SLC22A20*	Body	island	0.22	0.42	0.18	0.36	0.21
**TLE3**	cg20820622	15	70,354,647	*TLE3*	Body	open sea	0.64	0.50	0.68	0.55	0.61
	cg22537334	15	70,354,691	*TLE3*	Body	open sea	0.80	0.69	0.78	0.68	0.75
	cg21245875	15	70,354,825	*TLE3*	Body	open sea	0.67	0.55	0.68	0.55	0.63

**AGAP3**	cg25788549	7	150,786,044	*AGAP3*	Body	shore	0.30	0.48	0.23	0.43	0.27
	cg22169990	7	150,786,051	*AGAP3*	Body	shore	0.36	0.57	0.30	0.54	0.33
	cg21887193	7	150,786,082	*AGAP3*	Body	shore	0.29	0.50	0.21	0.43	0.25
**SULT2B1**	cg03039843	19	49,055,390	*SULT2B1*	TSS200	open sea	0.74	0.64	0.72	0.64	0.67
	cg23097961	19	49,055,412	*SULT2B1*	TSS200	open sea	0.64	0.54	0.67	0.57	0.67
	cg00698688	19	49,055,432	*SULT2B1*	1stExon	open sea	0.60	0.52	0.67	0.59	0.66
	cg08151612	19	49,055,438	*SULT2B1*	1stExon	open sea	0.59	0.51	0.62	0.56	0.62
	cg07543967	19	49,055,443	*SULT2B1*	1stExon	open sea	0.65	0.55	0.70	0.62	0.67
**IGR8**	cg23143104	14	103,691,361	intergenic	IGR	shelf	0.67	0.60	0.65	0.56	0.61
	cg23142048	14	103,691,563	intergenic	IGR	open sea	0.67	0.60	0.65	0.56	0.58
	cg01024455	14	103,691,569	intergenic	IGR	open sea	0.74	0.67	0.71	0.63	0.65
	cg07026636	14	103,691,799	intergenic	IGR	open sea	0.42	0.29	0.45	0.36	0.38
	cg18810310	14	103,691,834	intergenic	IGR	open sea	0.53	0.43	0.53	0.43	0.47
	cg16696727	14	103,691,932	intergenic	IGR	open sea	0.72	0.61	0.71	0.61	0.65
	cg18574274	14	103,692,015	intergenic	IGR	open sea	0.68	0.55	0.72	0.58	0.61
	cg04247336	14	103,692,104	intergenic	IGR	open sea	0.78	0.68	0.79	0.70	0.70
**LINC01550**	cg00263248	14	98,444,151	*LINC01550*	Body	open sea	0.71	0.54	0.72	0.58	0.62
	cg16062483	14	98,444,417	*LINC01550*	Body	open sea	0.61	0.44	0.71	0.55	0.66
	cg16278496	14	98,444,476	*LINC01550*	TSS200	open sea	0.43	0.29	0.47	0.37	0.48
	cg11798182	14	98,444,513	*LINC01550*	TSS200	open sea	0.59	0.44	0.55	0.44	0.52
	cg00034769	14	98,444,533	*LINC01550*	TSS200	open sea	0.48	0.39	0.42	0.36	0.41
**ALDH3B2**	cg20420868	11	67,442,067	*ALDH3B2*	1stExon	open sea	0.79	0.72	0.76	0.69	0.70
	cg07891457	11	67,442,075	*ALDH3B2*	1stExon	open sea	0.90	0.83	0.87	0.82	0.82
	cg18492926	11	67,442,195	*ALDH3B2*	5'UTR	open sea	0.72	0.67	0.76	0.69	0.69
	cg27123351	11	67,442,249	*ALDH3B2*	5'UTR	open sea	0.69	0.63	0.66	0.59	0.59
	cg24563501	11	67,442,408	*ALDH3B2*	TSS1500	open sea	0.81	0.74	0.79	0.72	0.75
**RHOD**	cg18043888	11	66,839,187	*RHOD*	3'UTR	island	0.25	0.39	0.26	0.35	0.28

	cg00023919	11	66,839,191	*RHOD*	3'UTR	island	0.33	0.49	0.28	0.38	0.29
	cg18407752	11	66,839,363	*RHOD*	3'UTR	shore	0.08	0.15	0.10	0.15	0.11
**IGR4**	cg23365135	7	525,684	intergenic	IGR	open sea	0.80	0.68	0.79	0.68	0.73
	cg06888346	7	525,853	intergenic	IGR	open sea	0.86	0.81	0.82	0.77	0.77
	cg05963354	7	525,943	intergenic	IGR	open sea	0.70	0.66	0.69	0.64	0.64
**SLC6A19**	cg02389859	5	1,201,691	*SLC6A19*	TSS200	island	0.72	0.62	0.73	0.62	0.69
	cg26948274	5	1,201,698	*SLC6A19*	TSS200	island	0.65	0.58	0.64	0.56	0.60
	cg17650028	5	1,201,713	*SLC6A19*	5'UTR	island	0.64	0.55	0.80	0.67	0.72
PHF13	cg00958217	1	6,681,584	*PHF13*	Body	shelf	0.52	0.71	0.42	0.56	0.42
	cg05377512	1	6,681,647	*PHF13*	Body	shelf	0.58	0.78	0.46	0.62	0.46
	cg15158876	1	6,681,878	*PHF13*	3'UTR	shelf	0.48	0.66	0.40	0.51	0.42
**ARRDC2**	cg05845141	19	18,120,614	*ARRDC2*	Body	island	0.76	0.61	0.74	0.57	0.67
	cg12965095	19	18,120,626	*ARRDC2*	Body	island	0.82	0.66	0.89	0.74	0.84
	cg12218406	19	18,120,692	*ARRDC2*	Body	island	0.82	0.71	0.89	0.76	0.83
**IGR11**	cg14897838	17	79,339,158	intergenic	IGR	open sea	0.39	0.26	0.45	0.33	0.44
	cg11225357	17	79,339,278	intergenic	IGR	open sea	0.70	0.59	0.74	0.65	0.68
	cg26148904	17	79,339,342	intergenic	IGR	open sea	0.48	0.29	0.53	0.39	0.46
**TIMP2**	cg07865166	17	76,920,397	*TIMP2*	Body	shore	0.28	0.15	0.33	0.24	0.33
	cg11342615	17	76,920,454	*TIMP2*	Body	shore	0.47	0.32	0.50	0.40	0.47
	cg05376904	17	76,920,464	*TIMP2*	Body	shore	0.43	0.27	0.46	0.36	0.43
**ITGA3**	cg00798317	17	48,153,934	*ITGA3*	Body	open sea	0.66	0.78	0.58	0.66	0.56
	cg23602058	17	48,154,061	*ITGA3*	Body	open sea	0.46	0.68	0.41	0.55	0.41
	cg21767759	17	48,154,356	*ITGA3*	Body	open sea	0.39	0.55	0.33	0.44	0.33
**IGR9**	cg27665571	16	70,624,354	intergenic	IGR	open sea	0.52	0.44	0.51	0.43	0.45
	cg10485485	16	70,624,357	intergenic	IGR	open sea	0.51	0.44	0.49	0.41	0.43
	cg00636508	16	70,624,430	intergenic	IGR	open sea	0.82	0.66	0.84	0.73	0.77
**IGR7**	cg15644756	12	69,198,743	intergenic	IGR	shelf	0.36	0.50	0.33	0.42	0.38

	cg13322954	12	69,198,953	intergenic	IGR	shelf	0.61	0.74	0.51	0.60	0.50
	cg10635494	12	69,199,037	intergenic	IGR	shelf	0.44	0.65	0.41	0.58	0.43
**AGRN**	cg01150641	1	976,168	*AGRN*	Body	island	0.26	0.39	0.26	0.35	0.19
	cg23625715	1	976,172	*AGRN*	Body	island	0.14	0.26	0.12	0.22	0.13
	cg26222311	1	976,227	*AGRN*	Body	island	0.18	0.32	0.16	0.26	0.17
**ARHGEF25**	cg22610645	12	58,003,898	*ARHGEF25*	TSS1500	island	0.45	0.56	0.47	0.58	0.42
	cg11211563	12	58,003,905	*ARHGEF25*	TSS1500	island	0.49	0.61	0.50	0.63	0.47
	cg15409097	12	58,003,925	*ARHGEF25*	TSS1500	island	0.36	0.48	0.26	0.37	0.31
**TINAGL1**	cg15120085	1	32,041,898	*TINAGL1*	TSS200	open sea	0.55	0.45	0.58	0.47	0.51
	cg00541683	1	32,041,933	*TINAGL1*	TSS200	open sea	0.28	0.22	0.33	0.26	0.29
	cg15079885	1	32,041,940	*TINAGL1*	TSS200	open sea	0.58	0.48	0.59	0.49	0.53
	cg22855405	1	32,042,037	*TINAGL1*	TSS200	open sea	0.49	0.31	0.56	0.41	0.50
	cg24873592	1	32,042,092	*TINAGL1*	5'UTR	open sea	0.36	0.25	0.51	0.36	0.47
	cg18107144	1	32,042,157	*TINAGL1*	5'UTR	open sea	0.42	0.29	0.52	0.39	0.52
	cg14869028	1	32,042,161	*TINAGL1*	5'UTR	open sea	0.43	0.29	0.50	0.39	0.46
**CASZ1**	cg26522708	1	10,839,450	*CASZ1*	5'UTR	open sea	0.72	0.60	0.83	0.72	0.77
	cg26689934	1	10,839,502	*CASZ1*	5'UTR	open sea	0.88	0.82	0.86	0.78	0.81
	cg25463742	1	10,839,574	*CASZ1*	5'UTR	open sea	0.69	0.59	0.85	0.75	0.79
QPRT	cg00572323	16	29,706,151	*QPRT*	Body	shore	0.46	0.58	0.41	0.49	0.41
	cg03488456	16	29,706,275	*QPRT*	Body	island	0.51	0.67	0.51	0.65	0.53
	cg01468711	16	29,706,291	*QPRT*	Body	island	0.56	0.73	0.47	0.59	0.48
**PWWP2B**	cg11579421	10	134,211,857	*PWWP2B*	Body	shore	0.67	0.58	0.63	0.54	0.59
	cg25303150	10	134,211,874	*PWWP2B*	Body	shore	0.57	0.50	0.60	0.52	0.58
	cg24085039	10	134,211,908	*PWWP2B*	Body	shore	0.58	0.49	0.65	0.54	0.62
BACH2	cg10365984	6	91,004,430	*BACH2*	5'UTR	shore	0.24	0.33	0.19	0.29	0.26
	cg09745430	6	91,004,452	*BACH2*	5'UTR	shore	0.31	0.41	0.25	0.34	0.29
	cg24667115	6	91,004,482	*BACH2*	5'UTR	shore	0.29	0.37	0.23	0.34	0.27

Network 2											
PEMT	cg21605540	17	17,485,823	*PEMT*	Body	open sea	0.38	0.46	0.33	0.37	0.35
	cg02094018	17	17,485,934	*PEMT*	Body	open sea	0.44	0.54	0.39	0.44	0.41
	cg02295973	17	17,486,105	*PEMT*	Body	open sea	0.52	0.58	0.45	0.49	0.45
LBX2-AS1	cg02100410	2	74,731,354	*LBX2-AS1*	Body	shore	0.46	0.53	0.38	0.44	0.38
	cg25251459	2	74,731,371	*LBX2-AS1*	Body	shore	0.41	0.47	0.33	0.37	0.35
	cg13407169	2	74,731,413	*LBX2-AS1*	Body	shore	0.30	0.36	0.24	0.28	0.31
RBP5_2	cg14672128	12	7,280,912	*RBP5*	Body	open sea	0.42	0.49	0.35	0.39	0.37
	cg12074585	12	7,280,958	*RBP5*	Body	open sea	0.48	0.54	0.46	0.51	0.49
	cg20315995	12	7,280,971	*RBP5*	Body	open sea	0.49	0.54	0.47	0.53	0.51
	cg24441911	12	7,280,988	*RBP5*	Body	open sea	0.50	0.57	0.42	0.46	0.44
	cg24319651	12	7,281,343	*RBP5*	1stExon	open sea	0.50	0.57	0.39	0.46	0.39
FTCD	cg04413147	21	47,575,134	*FTCD*	Body	open sea	0.37	0.44	0.30	0.35	0.33
	cg10394047	21	47,575,416	*FTCD*	1stExon	open sea	0.44	0.50	0.35	0.40	0.37
	cg09436823	21	47,575,498	*FTCD*	TSS200	open sea	0.36	0.41	0.34	0.39	0.40
	cg18024037	21	47,575,504	*FTCD*	TSS200	open sea	0.49	0.54	0.50	0.54	0.52
	cg25322086	21	47,575,547	*FTCD*	TSS200	open sea	0.41	0.48	0.36	0.39	0.38
ABCG5;ABCG8	cg01186613	2	44,065,003	*ABCG5;ABCG8*	Body	open sea	0.48	0.57	0.39	0.45	0.40
	cg16451365	2	44,065,056	*ABCG5;ABCG8*	Body	open sea	0.46	0.52	0.39	0.43	0.39
	cg00705576	2	44,065,259	*ABCG5;ABCG8*	Body	open sea	0.48	0.52	0.39	0.42	0.40
	cg11467440	2	44,065,278	*ABCG5;ABCG8*	Body	open sea	0.46	0.49	0.45	0.48	0.48
	cg13341470	2	44,065,332	*ABCG5;ABCG8*	Body	open sea	0.43	0.48	0.37	0.40	0.38
	cg11113753	2	44,065,383	*ABCG5;ABCG8*	Body	open sea	0.41	0.47	0.35	0.38	0.37
	cg18281102	2	44,065,550	*ABCG5;ABCG8*	Body	open sea	0.44	0.51	0.37	0.41	0.38
	cg00009421	2	44,065,571	*ABCG5;ABCG8*	Body	open sea	0.42	0.48	0.35	0.38	0.37
	cg04680150	2	44,065,627	*ABCG5;ABCG8*	Body	open sea	0.55	0.61	0.49	0.53	0.51
	cg00459909	2	44,065,720	*ABCG5;ABCG8*	TSS1500	open sea	0.46	0.52	0.43	0.47	0.43

	cg20926720	2	44,065,725	*ABCG5;ABCG8*	TSS1500	open sea	0.42	0.48	0.44	0.50	0.46
	cg07681696	2	44,065,858	*ABCG5;ABCG8*	5'UTR	open sea	0.48	0.55	0.43	0.47	0.43
	cg08453096	2	44,065,893	*ABCG5;ABCG8*	5'UTR	open sea	0.53	0.60	0.49	0.52	0.48
	cg03157395	2	44,065,964	*ABCG5;ABCG8*	TSS200	open sea	0.50	0.54	0.46	0.48	0.45
	cg25781162	2	44,065,996	*ABCG5;ABCG8*	TSS200	open sea	0.49	0.55	0.42	0.46	0.43
	cg05864261	2	44,066,030	*ABCG5;ABCG8*	TSS200	open sea	0.31	0.36	0.29	0.30	0.29
SGK2	cg06796271	20	42,187,587	*SGK2*	TSS200	open sea	0.43	0.50	0.37	0.42	0.39
	cg01021952	20	42,187,632	*SGK2*	TSS200	open sea	0.44	0.51	0.37	0.41	0.37
	cg06600331	20	42,187,650	*SGK2*	TSS200	open sea	0.41	0.47	0.34	0.37	0.37
	cg17611262	20	42,187,750	*SGK2*	1stExon	open sea	0.46	0.52	0.38	0.42	0.40
	cg17463527	20	42,187,837	*SGK2*	5'UTR	open sea	0.40	0.47	0.35	0.39	0.37
RBP5_1	cg00294025	12	7,276,360	*RBP5*	3'UTR	open sea	0.42	0.53	0.34	0.42	0.35
	cg10993460	12	7,276,482	*RBP5*	3'UTR	open sea	0.29	0.37	0.23	0.28	0.24
	cg16959747	12	7,276,714	*RBP5*	3'UTR	open sea	0.30	0.38	0.25	0.31	0.28
APOC4	cg17769836	19	45,445,437	*APOC4*	TSS200	open sea	0.42	0.49	0.34	0.37	0.36
	cg04401876	19	45,445,449	*APOC4*	TSS200	open sea	0.41	0.48	0.35	0.39	0.37
	cg04347059	19	45,445,486	*APOC4*	TSS200	open sea	0.51	0.54	0.51	0.55	0.53
	cg02912790	19	45,445,491	*APOC4*	TSS200	open sea	0.46	0.50	0.36	0.39	0.37
	cg27353824	19	45,445,521	*APOC4*	5'UTR	open sea	0.42	0.47	0.36	0.39	0.36
	cg25017250	19	45,445,693	*APOC4*	Body	open sea	0.35	0.43	0.31	0.36	0.30
RGS12_3	cg09912079	4	3,409,828	*RGS12*	Body	open sea	0.38	0.45	0.30	0.34	0.31
	cg25447202	4	3,409,885	*RGS12*	Body	open sea	0.42	0.51	0.35	0.41	0.36
	cg06353485	4	3,409,927	*RGS12*	Body	open sea	0.43	0.52	0.38	0.41	0.39
	cg11463380	4	3,410,693	*RGS12*	Body	shelf	0.44	0.57	0.33	0.43	0.36
CHID1	cg07659663	11	914,329	*CHID1*	5'UTR	shelf	0.46	0.60	0.39	0.48	0.44
	cg23202388	11	914,849	*CHID1*	1stExon	shelf	0.31	0.42	0.26	0.31	0.29
	cg10668614	11	915,163	*CHID1*	TSS200	shelf	0.32	0.38	0.26	0.29	0.29

	cg06639440	11	915,170	*CHID1*	TSS200	shelf	0.34	0.42	0.28	0.32	0.31
	cg16402814	11	915,227	*CHID1*	TSS200	shelf	0.30	0.37	0.23	0.26	0.26
	cg18205787	11	915,239	*CHID1*	TSS200	shelf	0.34	0.41	0.30	0.36	0.36
	cg23449764	11	915,337	*CHID1*	TSS1500	shelf	0.34	0.43	0.29	0.34	0.31
	cg14166189	11	915,440	*CHID1*	TSS1500	shelf	0.36	0.47	0.30	0.36	0.34
HGFAC	cg23922755	4	3,443,656	*HGFAC*	TSS200	open sea	0.46	0.50	0.47	0.51	0.48
	cg26955579	4	3,443,683	*HGFAC*	TSS200	open sea	0.44	0.49	0.40	0.43	0.40
	cg20533530	4	3,443,694	*HGFAC*	TSS200	open sea	0.48	0.54	0.47	0.52	0.50
	cg17322505	4	3,443,735	*HGFAC*	1stExon	open sea	0.53	0.57	0.50	0.52	0.49
	cg07364841	4	3,444,147	*HGFAC*	Body	open sea	0.47	0.57	0.41	0.50	0.42
GCK	cg17650622	7	44,198,725	*GCK*	1stExon	open sea	0.48	0.53	0.46	0.51	0.50
	cg21504093	7	44,199,012	*GCK*	Body	open sea	0.52	0.59	0.45	0.49	0.45
	cg20035206	7	44,199,106	*GCK*	Body	open sea	0.45	0.51	0.37	0.40	0.40
	cg03345391	7	44,199,266	*GCK*	Body	open sea	0.85	0.83	0.82	0.81	0.76
	cg21987356	7	44,199,597	*GCK*	Body	open sea	0.52	0.59	0.42	0.47	0.42
PGLYRP2	cg17915429	19	15,590,069	*PGLYRP2*	Body	open sea	0.40	0.46	0.29	0.34	0.31
	cg17752089	19	15,590,308	*PGLYRP2*	5'UTR	open sea	0.49	0.54	0.40	0.44	0.40
	cg09054960	19	15,590,328	*PGLYRP2*	TSS200	open sea	0.43	0.47	0.34	0.37	0.35
	cg22310770	19	15,590,368	*PGLYRP2*	TSS200	open sea	0.49	0.55	0.42	0.47	0.42
	cg07408456	19	15,590,532	*PGLYRP2*	TSS1500	open sea	0.27	0.32	0.22	0.26	0.24
	cg17473673	19	15,590,570	*PGLYRP2*	TSS1500	open sea	0.27	0.33	0.23	0.27	0.25
TBCD	cg01771850	17	80,847,137	*TBCD*	Body	open sea	0.46	0.54	0.40	0.46	0.40
	cg05398905	17	80,847,209	*TBCD*	Body	open sea	0.43	0.51	0.38	0.42	0.40
	cg03535099	17	80,847,270	*TBCD*	Body	open sea	0.40	0.49	0.37	0.43	0.39
	cg09152949	17	80,847,497	*TBCD*	Body	open sea	0.58	0.67	0.59	0.61	0.52
	cg21156912	17	80,847,546	*TBCD*	Body	open sea	0.47	0.56	0.40	0.44	0.40
	cg19788754	17	80,847,662	*TBCD*	Body	open sea	0.60	0.66	0.54	0.57	0.50

SLC7A5	cg03553613	16	87,879,502	*SLC7A5*	Body	open sea	0.48	0.52	0.47	0.50	0.51
	cg04171052	16	87,879,581	*SLC7A5*	Body	open sea	0.49	0.56	0.43	0.47	0.44
	cg26637881	16	87,879,698	*SLC7A5*	Body	open sea	0.54	0.60	0.48	0.52	0.49
IRF7	cg18477816	11	612,588	*IRF7*	3'UTR	shore	0.58	0.68	0.50	0.57	0.50
	cg03755158	11	612,680	*IRF7*	Body	shore	0.49	0.56	0.42	0.47	0.43
	cg27271532	11	612,762	*IRF7*	Body	shore	0.46	0.53	0.38	0.42	0.39
	cg05309505	11	612,837	*IRF7*	Body	shore	0.43	0.50	0.36	0.42	0.43
HNF4A	cg08314996	20	42,984,099	*HNF4A*	TSS1500	open sea	0.35	0.41	0.32	0.36	0.35
	cg16121136	20	42,984,209	*HNF4A*	TSS1500	open sea	0.44	0.48	0.38	0.39	0.40
	cg24084358	20	42,984,276	*HNF4A*	TSS200	open sea	0.47	0.52	0.41	0.44	0.43
	cg06126829	20	42,984,320	*HNF4A*	TSS200	open sea	0.47	0.53	0.43	0.45	0.43
	cg06640637	20	42,984,324	*HNF4A*	TSS200	open sea	0.49	0.55	0.46	0.52	0.53
	cg20848979	20	42,984,338	*HNF4A*	TSS200	open sea	0.51	0.56	0.50	0.54	0.55
	cg22958104	20	42,984,347	*HNF4A*	TSS200	open sea	0.52	0.56	0.46	0.48	0.46
	cg16221969	20	42,984,394	*HNF4A*	TSS200	open sea	0.49	0.54	0.42	0.45	0.44
	cg23792485	20	42,984,453	*HNF4A*	1stExon	open sea	0.49	0.55	0.48	0.53	0.53
	cg21081369	20	42,984,579	*HNF4A*	Body	open sea	0.40	0.46	0.35	0.38	0.38
	cg19717150	20	42,984,878	*HNF4A*	Body	open sea	0.32	0.36	0.25	0.29	0.30
MIR192;MIR194-2	cg05560951	11	64,658,226	*MIR192;MIR194-2*	IGR	open sea	0.74	0.83	0.60	0.67	0.58
	cg02258444	11	64,658,622	*MIR192;MIR194-2*	Body	shelf	0.47	0.56	0.37	0.41	0.39
	cg27083891	11	64,658,726	*MIR192;MIR194-2*	TSS200	shelf	0.46	0.53	0.44	0.49	0.48
	cg09349409	11	64,658,765	*MIR192;MIR194-2*	TSS200	shelf	0.50	0.55	0.43	0.46	0.44
	cg18262830	11	64,658,819	*MIR192;MIR194-2*	TSS200	shelf	0.44	0.50	0.38	0.41	0.39
	cg24803202	11	64,658,903	*MIR192;MIR194-2*	Body	shelf	0.42	0.49	0.36	0.40	0.38
	cg08432452	11	64,658,936	*MIR192;MIR194-2*	TSS1500	shelf	0.54	0.58	0.50	0.52	0.49
	cg00589493	11	64,658,940	*MIR192;MIR194-2*	TSS1500	shelf	0.45	0.50	0.41	0.43	0.42
	cg13092487	11	64,658,946	*MIR192;MIR194-2*	TSS1500	shelf	0.43	0.49	0.39	0.42	0.40

	cg24154336	11	64,659,044	*MIR192;MIR194-2*	TSS1500	shelf	0.45	0.49	0.44	0.48	0.47
	cg00400165	11	64,659,060	*MIR192;MIR194-2*	TSS1500	shelf	0.44	0.51	0.41	0.45	0.41
	cg00376448	11	64,659,065	*MIR192;MIR194-2*	TSS1500	shelf	0.44	0.48	0.39	0.43	0.39
	cg02494703	11	64,659,387	*MIR192;MIR194-2*	TSS1500	shelf	0.24	0.28	0.23	0.25	0.24
MIR629;TLE3	cg05185738	15	70,371,992	*MIR629;TLE3*	TSS200	open sea	0.52	0.59	0.44	0.49	0.44
	cg17972789	15	70,372,105	*MIR629;TLE3*	Body	open sea	0.45	0.52	0.39	0.44	0.39
	cg13912196	15	70,372,127	*MIR629;TLE3*	Body	open sea	0.52	0.60	0.45	0.50	0.42
	cg02852421	15	70,372,614	*MIR629;TLE3*	Body	open sea	0.41	0.48	0.33	0.38	0.34
NCOA4	cg06098215	10	51,575,702	*NCOA4*	5'UTR	shelf	0.23	0.32	0.16	0.21	0.17
	cg16814786	10	51,575,763	*NCOA4*	5'UTR	shelf	0.28	0.35	0.19	0.24	0.21
	cg01315067	10	51,576,232	*NCOA4*	TSS200	shelf	0.42	0.48	0.32	0.37	0.31
	cg00302587	10	51,576,241	*NCOA4*	TSS200	shelf	0.46	0.55	0.36	0.42	0.37
	cg20166027	10	51,576,452	*NCOA4*	1stExon	shelf	0.32	0.42	0.22	0.28	0.24
LIMS2	cg13099839	2	128,422,113	*LIMS2*	Body	island	0.18	0.22	0.17	0.19	0.17
	cg16944093	2	128,422,120	*LIMS2*	Body	island	0.20	0.24	0.19	0.22	0.21
	cg10661054	2	128,422,179	*LIMS2*	Body	island	0.16	0.20	0.11	0.16	0.16
	cg19426955	2	128,422,224	*LIMS2*	Body	shore	0.33	0.38	0.29	0.34	0.33
	cg11535366	2	128,422,284	*LIMS2*	Body	shore	0.43	0.51	0.37	0.43	0.37
	cg18044111	2	128,422,307	*LIMS2*	Body	shore	0.45	0.53	0.39	0.44	0.40
	cg21339084	2	128,422,433	*LIMS2*	Body	shore	0.42	0.49	0.36	0.40	0.36
	cg14489933	2	128,422,619	*LIMS2*	Body	shore	0.46	0.55	0.37	0.44	0.39
	cg19326543	2	128,422,715	*LIMS2*	Body	shore	0.46	0.55	0.39	0.45	0.41
	cg15781838	2	128,422,717	*LIMS2*	Body	shore	0.49	0.58	0.39	0.46	0.41
FABP1	cg19910382	2	88,427,561	*FABP1*	1stExon	open sea	0.47	0.53	0.40	0.44	0.40
	cg19217130	2	88,427,581	*FABP1*	TSS200	open sea	0.36	0.41	0.33	0.38	0.37
	cg24046616	2	88,427,630	*FABP1*	TSS200	open sea	0.44	0.48	0.36	0.40	0.36
	cg24933157	2	88,427,637	*FABP1*	TSS200	open sea	0.42	0.48	0.35	0.39	0.36

WWP2	cg16439003	16	69,975,149	*WWP2*	3'UTR	open sea	0.88	0.90	0.78	0.82	0.77
	cg03840678	16	69,975,320	*WWP2*	3'UTR	open sea	0.46	0.53	0.39	0.43	0.39
	cg26481896	16	69,975,405	*WWP2*	3'UTR	open sea	0.41	0.51	0.34	0.40	0.35
	cg27186420	16	69,975,590	*WWP2*	3'UTR	open sea	0.49	0.61	0.42	0.50	0.43
	cg26821137	16	69,976,040	*WWP2*	IGR	open sea	0.36	0.45	0.32	0.36	0.33
	cg00549064	16	69,976,228	*WWP2*	IGR	open sea	0.42	0.51	0.35	0.42	0.36
NUPR1	cg08818284	16	28,548,873	*NUPR1*	3'UTR	open sea	0.44	0.51	0.37	0.39	0.37
	cg06723057	16	28,549,923	*NUPR1*	Body	open sea	0.40	0.44	0.35	0.37	0.37
	cg05590982	16	28,550,171	*NUPR1*	1stExon	open sea	0.26	0.32	0.22	0.25	0.23
	cg04492847	16	28,550,525	*NUPR1*	TSS200	open sea	0.40	0.44	0.36	0.40	0.38
	cg06288570	16	28,550,567	*NUPR1*	TSS200	open sea	0.23	0.27	0.24	0.25	0.24
	cg15149645	16	28,550,619	*NUPR1*	TSS200	open sea	0.42	0.49	0.37	0.42	0.35
	cg01542023	16	28,550,637	*NUPR1*	TSS200	open sea	0.36	0.41	0.34	0.38	0.34
CUX1	cg19346623	7	101,901,663	*CUX1*	Body	open sea	0.43	0.50	0.41	0.48	0.45
	cg02611466	7	101,901,707	*CUX1*	Body	open sea	0.48	0.56	0.40	0.45	0.40
	cg04151469	7	101,901,764	*CUX1*	Body	open sea	0.45	0.57	0.38	0.46	0.39
PROC	cg25457027	2	128,175,179	*PROC*	TSS1500	shore	0.79	0.83	0.72	0.72	0.68
	cg10021288	2	128,175,891	*PROC*	TSS200	shelf	0.54	0.59	0.44	0.51	0.44
	cg22856114	2	128,175,905	*PROC*	TSS200	shelf	0.52	0.58	0.44	0.49	0.45
	cg06038358	2	128,176,007	*PROC*	5'UTR	shelf	0.47	0.50	0.45	0.49	0.49
	cg26718585	2	128,176,454	*PROC*	5'UTR	shelf	0.54	0.60	0.45	0.48	0.45
	cg11143063	2	128,177,475	*PROC*	5'UTR	shelf	0.47	0.53	0.39	0.43	0.40
IGR2	cg11847956	5	173,198,447	intergenic	IGR	open sea	0.39	0.48	0.30	0.36	0.33
	cg11212451	5	173,198,508	intergenic	IGR	open sea	0.31	0.39	0.27	0.35	0.32
	cg10818423	5	173,198,602	intergenic	IGR	open sea	0.41	0.48	0.33	0.37	0.35
C12orf74	cg04614923	12	93,096,622	*C12orf74*	TSS1500	open sea	0.26	0.26	0.23	0.24	0.25
	cg12755471	12	93,096,780	*C12orf74*	TSS200	open sea	0.47	0.54	0.37	0.41	0.38

	cg27576271	12	93,096,811	*C12orf74*	TSS200	open sea	0.54	0.62	0.46	0.51	0.46
	cg26383838	12	93,096,819	*C12orf74*	TSS200	open sea	0.51	0.58	0.42	0.47	0.43
	cg25382573	12	93,096,844	*C12orf74*	5'UTR	open sea	0.35	0.42	0.34	0.39	0.38
IGR6	cg19299755	11	116,706,051	intergenic	IGR	shore	0.42	0.46	0.38	0.44	0.41
	cg23193059	11	116,706,090	intergenic	IGR	shore	0.31	0.41	0.24	0.31	0.25
	cg03044513	11	116,706,153	intergenic	IGR	shore	0.32	0.38	0.25	0.29	0.26
RTKN	cg03295928	2	74,669,349	*RTKN*	TSS1500	island	0.28	0.33	0.27	0.31	0.32
	cg14481208	2	74,669,375	*RTKN*	TSS1500	island	0.30	0.38	0.28	0.35	0.33
	cg00658082	2	74,669,380	*RTKN*	TSS1500	island	0.41	0.47	0.35	0.38	0.36
	cg26090072	2	74,669,387	*RTKN*	TSS1500	island	0.36	0.43	0.29	0.34	0.31
FOXP2	cg01306563	7	114,055,074	*FOXP2*	1stExon	open sea	0.14	0.16	0.13	0.15	0.13
	cg18546840	7	114,055,123	*FOXP2*	1stExon	open sea	0.35	0.40	0.30	0.35	0.26
	cg24786986	7	114,055,133	*FOXP2*	1stExon	open sea	0.34	0.42	0.29	0.35	0.26
	cg18871253	7	114,055,137	*FOXP2*	1stExon	open sea	0.31	0.37	0.27	0.33	0.26
	cg19655952	7	114,055,204	*FOXP2*	1stExon	open sea	0.24	0.30	0.21	0.25	0.21
	cg02211646	7	114,055,210	*FOXP2*	1stExon	open sea	0.33	0.41	0.26	0.32	0.27
IGR3	cg08614082	6	158,375,684	intergenic	IGR	open sea	0.26	0.32	0.23	0.28	0.23
	cg27359374	6	158,375,774	intergenic	IGR	open sea	0.32	0.37	0.27	0.32	0.33
	cg11051752	6	158,375,814	intergenic	IGR	open sea	0.41	0.48	0.35	0.39	0.36
CLU	cg08594681	8	27,468,684	*CLU*	1stExon	shelf	0.28	0.33	0.24	0.28	0.26
	cg22313574	8	27,468,981	*CLU*	TSS200	shelf	0.27	0.33	0.25	0.27	0.25
	cg14917244	8	27,469,001	*CLU*	TSS200	shelf	0.22	0.27	0.20	0.23	0.20
	cg12729838	8	27,469,186	*CLU*	1stExon	shelf	0.36	0.41	0.30	0.32	0.33
	cg11783834	8	27,469,331	*CLU*	Body	shelf	0.27	0.31	0.22	0.28	0.27
	cg13488078	8	27,469,338	*CLU*	Body	shelf	0.37	0.43	0.31	0.36	0.33
	cg00359590	8	27,469,673	*CLU*	TSS1500	shelf	0.38	0.44	0.28	0.32	0.31
	cg19442470	8	27,470,225	*CLU*	TSS1500	shore	0.45	0.52	0.38	0.43	0.38

DYNC1I1	cg09136052	7	95,546,508	*DYNC1I1*	Body	open sea	0.50	0.61	0.41	0.48	0.41
	cg18450582	7	95,546,539	*DYNC1I1*	Body	open sea	0.23	0.31	0.17	0.25	0.20
	cg23975973	7	95,546,556	*DYNC1I1*	Body	open sea	0.30	0.38	0.24	0.31	0.23

DMRs in networks 1 and 2 observed in both the Japanese and American NAFLD cohorts. DMRs were sorted according to the number of edges connected to DMRs in the Japanese NAFLD cohort. In network 1, 23 DMRs were observed in the control group and are represented in bold.

Supplementary Table 3. Characteristics of 62 DMRs associated with NAFLD progression.

DMR (node)	Probe	Chr	Position (hg19)	Annotated genes	CpG feature		Correlation									
							Fibrosis stage		Lobular inflammation		Type IV collagen 7S		Age		Fasting plasma glucose	
					Location	Island	r	*p*	r	*p*	r	p	r	p	r	p
Network 1																
**ZBTB38**	cg06137072	3	141,087,187	*ZBTB38*	5'UTR	open sea	-0.49	8.0E-05	-0.31	1.5E-02	-0.52	2.5E-05	-0.54	7.1E-06	-0.47	1.3E-04
	cg13029400	3	141,087,190	*ZBTB38*	5'UTR	open sea	-0.46	2.3E-04	-0.29	2.5E-02	-0.48	9.8E-05	-0.56	3.2E-06	-0.48	1.1E-04
	cg08360599	3	141,087,261	*ZBTB38*	5'UTR	open sea	-0.45	2.8E-04	-0.29	2.3E-02	-0.49	6.7E-05	-0.55	4.5E-06	-0.46	2.3E-04
	cg21370924	3	141,087,313	*ZBTB38*	5'UTR	open sea	-0.50	5.8E-05	-0.33	1.1E-02	-0.53	1.1E-05	-0.56	3.8E-06	-0.49	7.6E-05
	cg21474062	3	141,087,363	*ZBTB38*	5'UTR	open sea	-0.46	2.5E-04	-0.31	1.6E-02	-0.50	5.7E-05	-0.57	1.6E-06	-0.48	8.7E-05
**C2CD4D**	cg15015892	1	151,810,887	*C2CD4D*	Body	island	0.48	1.1E-04	0.27	3.5E-02	0.50	4.2E-05	0.55	5.9E-06	0.47	1.6E-04
	cg05021743	1	151,810,893	*C2CD4D*	Body	island	0.48	1.1E-04	0.31	1.8E-02	0.50	4.5E-05	0.56	3.3E-06	0.48	1.0E-04
	cg10781408	1	151,810,899	*C2CD4D*	Body	island	0.48	1.0E-04	0.30	1.9E-02	0.50	4.9E-05	0.55	4.5E-06	0.46	2.2E-04
	cg04296699	1	151,810,904	*C2CD4D*	Body	island	0.47	1.7E-04	0.31	1.6E-02	0.49	6.2E-05	0.55	4.2E-06	0.46	2.3E-04
**GPR56**	cg01410801	16	57,653,669	*GPR56*	TSS1500	open sea	-0.46	2.0E-04	-0.35	5.5E-03	-0.50	4.9E-05	-0.57	2.0E-06	-0.42	7.6E-04
	cg25645462	16	57,653,702	*GPR56*	TSS1500	open sea	-0.44	4.9E-04	-0.32	1.3E-02	-0.47	1.5E-04	-0.55	6.1E-06	-0.36	4.3E-03
	cg00550797	16	57,653,904	*GPR56*	TSS200	open sea	-0.41	1.3E-03	-0.31	1.8E-02	-0.44	5.1E-04	-0.58	1.2E-06	-0.39	2.4E-03
	cg16630572	16	57,653,917	*GPR56*	5'UTR	open sea	-0.41	1.2E-03	-0.31	1.6E-02	-0.44	4.6E-04	-0.58	1.2E-06	-0.42	8.4E-04
**FMN1**	cg15175581	15	33,360,262	*FMN1*	TSS200	open sea	-0.52	1.8E-05	-0.30	2.1E-02	-0.50	5.4E-05	-0.64	2.7E-08	-0.46	2.5E-04
	cg09347959	15	33,360,271	*FMN1*	TSS200	open sea	-0.53	1.2E-05	-0.33	1.0E-02	-0.52	2.3E-05	-0.64	4.0E-08	-0.50	4.2E-05
	cg17454592	15	33,360,353	*FMN1*	TSS1500	open sea	-0.46	2.3E-04	-0.24	6.5E-02	-0.46	2.2E-04	-0.50	4.0E-05	-0.51	2.7E-05
**SLC22A20**	cg02675946	11	64,993,239	*SLC22A20*	Body	shore	0.47	1.7E-04	0.26	4.7E-02	0.44	4.7E-04	0.54	9.4E-06	0.52	2.4E-05
	cg27428304	11	64,993,281	*SLC22A20*	Body	island	0.47	1.8E-04	0.25	5.1E-02	0.44	3.8E-04	0.51	2.8E-05	0.50	5.1E-05
	cg23930334	11	64,993,335	*SLC22A20*	Body	island	0.51	3.4E-05	0.32	1.3E-02	0.50	4.9E-05	0.60	4.0E-07	0.51	2.6E-05
**TLE3**	cg20820622	15	70,354,647	*TLE3*	Body	open sea	-0.53	1.5E-05	-0.35	6.4E-03	-0.54	1.0E-05	-0.53	1.6E-05	-0.53	1.7E-05
	cg22537334	15	70,354,691	*TLE3*	Body	open sea	-0.51	3.7E-05	-0.32	1.2E-02	-0.50	5.5E-05	-0.50	4.7E-05	-0.45	3.1E-04

	cg21245875	15	70,354,825	*TLE3*	Body	open sea	-0.51	3.1E-05	-0.34	7.5E-03	-0.52	2.1E-05	-0.54	7.9E-06	-0.49	6.2E-05
**AGAP3**	cg25788549	7	150,786,044	*AGAP3*	Body	shore	0.52	1.9E-05	0.30	1.9E-02	0.52	2.0E-05	0.55	6.5E-06	0.47	1.6E-04
	cg22169990	7	150,786,051	*AGAP3*	Body	shore	0.54	8.7E-06	0.35	5.6E-03	0.57	1.9E-06	0.58	9.6E-07	0.49	6.6E-05
	cg21887193	7	150,786,082	*AGAP3*	Body	shore	0.56	2.6E-06	0.38	3.1E-03	0.57	2.3E-06	0.58	1.0E-06	0.51	3.9E-05
**SULT2B1**	cg03039843	19	49,055,390	*SULT2B1*	TSS200	open sea	-0.42	8.8E-04	-0.25	5.4E-02	-0.37	3.2E-03	-0.58	1.5E-06	-0.46	1.9E-04
	cg23097961	19	49,055,412	*SULT2B1*	TSS200	open sea	-0.49	7.0E-05	-0.30	2.1E-02	-0.47	1.5E-04	-0.58	1.0E-06	-0.49	8.3E-05
	cg00698688	19	49,055,432	*SULT2B1*	1stExon	open sea	-0.44	4.7E-04	-0.24	6.6E-02	-0.38	2.7E-03	-0.54	1.1E-05	-0.43	6.7E-04
	cg08151612	19	49,055,438	*SULT2B1*	1stExon	open sea	-0.51	2.6E-05	-0.30	2.1E-02	-0.48	9.4E-05	-0.56	3.4E-06	-0.48	1.2E-04
	cg07543967	19	49,055,443	*SULT2B1*	1stExon	open sea	-0.52	2.0E-05	-0.33	8.9E-03	-0.47	1.5E-04	-0.60	3.7E-07	-0.49	8.1E-05
**IGR8**	cg23143104	14	103,691,361	*intergenic*	IGR	shelf	-0.34	7.2E-03	-0.21	1.1E-01	-0.36	4.7E-03	-0.51	2.9E-05	-0.48	1.1E-04
	cg23142048	14	103,691,563	*intergenic*	IGR	open sea	-0.34	7.5E-03	-0.21	1.1E-01	-0.35	5.9E-03	-0.58	1.5E-06	-0.37	3.6E-03
	cg01024455	14	103,691,569	*intergenic*	IGR	open sea	-0.37	3.2E-03	-0.22	9.2E-02	-0.36	4.5E-03	-0.55	4.9E-06	-0.35	5.9E-03
	cg07026636	14	103,691,799	*intergenic*	IGR	open sea	-0.46	2.4E-04	-0.31	1.7E-02	-0.44	3.8E-04	-0.54	7.1E-06	-0.49	8.2E-05
	cg18810310	14	103,691,834	*intergenic*	IGR	open sea	-0.42	8.5E-04	-0.29	2.2E-02	-0.42	7.3E-04	-0.52	2.0E-05	-0.41	1.1E-03
	cg16696727	14	103,691,932	*intergenic*	IGR	open sea	-0.44	4.4E-04	-0.30	2.1E-02	-0.45	2.6E-04	-0.57	1.7E-06	-0.38	2.9E-03
	cg18574274	14	103,692,015	*intergenic*	IGR	open sea	-0.39	2.2E-03	-0.29	2.6E-02	-0.44	3.8E-04	-0.55	5.7E-06	-0.46	2.4E-04
	cg04247336	14	103,692,104	*intergenic*	IGR	open sea	-0.43	5.3E-04	-0.28	3.0E-02	-0.45	2.6E-04	-0.54	1.0E-05	-0.36	4.9E-03
**LINC01550**	cg00263248	14	98,444,151	*LINC01550*	Body	open sea	-0.51	3.4E-05	-0.26	4.3E-02	-0.51	3.7E-05	-0.58	9.3E-07	-0.54	9.9E-06
	cg16062483	14	98,444,417	*LINC01550*	Body	open sea	-0.52	1.9E-05	-0.29	2.4E-02	-0.52	1.7E-05	-0.59	8.6E-07	-0.52	1.9E-05
	cg16278496	14	98,444,476	*LINC01550*	TSS200	open sea	-0.54	9.9E-06	-0.35	5.6E-03	-0.48	1.3E-04	-0.63	7.9E-08	-0.50	4.3E-05
	cg11798182	14	98,444,513	*LINC01550*	TSS200	open sea	-0.52	2.4E-05	-0.32	1.1E-02	-0.50	5.3E-05	-0.59	5.6E-07	-0.46	2.5E-04
	cg00034769	14	98,444,533	*LINC01550*	TSS200	open sea	-0.51	3.8E-05	-0.27	3.5E-02	-0.46	2.0E-04	-0.59	5.6E-07	-0.45	3.4E-04
**ALDH3B2**	cg20420868	11	67,442,067	*ALDH3B2*	1stExon	open sea	-0.36	4.9E-03	-0.18	1.7E-01	-0.33	1.1E-02	-0.46	2.2E-04	-0.42	7.7E-04
	cg07891457	11	67,442,075	*ALDH3B2*	1stExon	open sea	-0.44	4.7E-04	-0.19	1.5E-01	-0.40	1.6E-03	-0.47	1.5E-04	-0.41	1.2E-03
	cg18492926	11	67,442,195	*ALDH3B2*	5'UTR	open sea	-0.37	3.7E-03	-0.23	7.2E-02	-0.37	3.5E-03	-0.47	1.4E-04	-0.38	2.9E-03
	cg27123351	11	67,442,249	*ALDH3B2*	5'UTR	open sea	-0.33	9.0E-03	-0.15	2.5E-01	-0.33	9.7E-03	-0.35	6.3E-03	-0.33	9.5E-03
	cg24563501	11	67,442,408	*ALDH3B2*	TSS1500	open sea	-0.47	1.4E-04	-0.30	2.0E-02	-0.48	1.2E-04	-0.52	2.3E-05	-0.42	9.1E-04

**RHOD**	cg18043888	11	66,839,187	*RHOD*	3'UTR	island	0.54	9.2E-06	0.30	1.9E-02	0.55	4.3E-06	0.60	4.8E-07	0.52	1.9E-05
	cg00023919	11	66,839,191	*RHOD*	3'UTR	island	0.53	1.6E-05	0.33	8.9E-03	0.56	2.8E-06	0.58	9.5E-07	0.49	8.4E-05
	cg18407752	11	66,839,363	*RHOD*	3'UTR	shore	0.43	6.9E-04	0.22	9.3E-02	0.36	4.9E-03	0.46	2.0E-04	0.43	6.8E-04
**IGR4**	cg23365135	7	525,684	*intergenic*	IGR	open sea	-0.39	2.0E-03	-0.22	8.7E-02	-0.39	1.9E-03	-0.54	9.5E-06	-0.37	3.8E-03
	cg06888346	7	525,853	*intergenic*	IGR	open sea	-0.38	2.5E-03	-0.23	8.0E-02	-0.40	1.6E-03	-0.50	4.4E-05	-0.43	7.0E-04
	cg05963354	7	525,943	*intergenic*	IGR	open sea	-0.37	4.0E-03	-0.18	1.8E-01	-0.39	1.8E-03	-0.48	1.2E-04	-0.33	9.9E-03
**SLC6A19**	cg02389859	5	1,201,691	*SLC6A19*	TSS200	island	-0.39	1.9E-03	-0.32	1.3E-02	-0.36	5.4E-03	-0.53	1.3E-05	-0.36	4.8E-03
	cg26948274	5	1,201,698	*SLC6A19*	TSS200	island	-0.35	5.7E-03	-0.32	1.3E-02	-0.32	1.4E-02	-0.52	2.0E-05	-0.29	2.6E-02
	cg17650028	5	1,201,713	*SLC6A19*	5'UTR	island	-0.44	5.0E-04	-0.34	7.0E-03	-0.42	7.2E-04	-0.58	1.4E-06	-0.38	2.6E-03
PHF13	cg00958217	1	6,681,584	*PHF13*	Body	shelf	0.54	1.0E-05	0.42	8.4E-04	0.58	1.2E-06	0.50	4.2E-05	0.48	9.3E-05
	cg05377512	1	6,681,647	*PHF13*	Body	shelf	0.54	9.0E-06	0.39	2.3E-03	0.58	1.4E-06	0.47	1.5E-04	0.52	2.3E-05
	cg15158876	1	6,681,878	*PHF13*	3'UTR	shelf	0.51	2.6E-05	0.38	3.1E-03	0.56	3.5E-06	0.43	6.8E-04	0.51	2.6E-05
**ARRDC2**	cg05845141	19	18,120,614	*ARRDC2*	Body	island	-0.53	1.3E-05	-0.28	2.7E-02	-0.49	6.2E-05	-0.55	5.3E-06	-0.46	2.0E-04
	cg12965095	19	18,120,626	*ARRDC2*	Body	island	-0.54	9.8E-06	-0.32	1.2E-02	-0.51	2.6E-05	-0.54	9.1E-06	-0.47	1.6E-04
	cg12218406	19	18,120,692	*ARRDC2*	Body	island	-0.54	7.3E-06	-0.30	1.9E-02	-0.50	4.4E-05	-0.37	3.6E-03	-0.36	4.5E-03
**IGR11**	cg14897838	17	79,339,158	*intergenic*	IGR	open sea	-0.49	5.9E-05	-0.35	5.7E-03	-0.52	2.1E-05	-0.52	2.4E-05	-0.51	3.7E-05
	cg11225357	17	79,339,278	*intergenic*	IGR	open sea	-0.50	5.4E-05	-0.28	3.3E-02	-0.54	9.1E-06	-0.59	8.8E-07	-0.42	7.9E-04
	cg26148904	17	79,339,342	*intergenic*	IGR	open sea	-0.52	1.8E-05	-0.35	5.8E-03	-0.53	1.1E-05	-0.57	2.3E-06	-0.50	4.2E-05
**TIMP2**	cg07865166	17	76,920,397	*TIMP2*	Body	shore	-0.53	1.6E-05	-0.35	5.4E-03	-0.54	1.0E-05	-0.61	1.7E-07	-0.48	1.2E-04
	cg11342615	17	76,920,454	*TIMP2*	Body	shore	-0.53	1.6E-05	-0.36	4.5E-03	-0.61	2.6E-07	-0.55	4.2E-06	-0.54	1.0E-05
	cg05376904	17	76,920,464	*TIMP2*	Body	shore	-0.51	2.6E-05	-0.35	5.5E-03	-0.54	7.3E-06	-0.57	2.0E-06	-0.49	6.3E-05
**ITGA3**	cg00798317	17	48,153,934	*ITGA3*	Body	open sea	0.45	3.5E-04	0.37	3.8E-03	0.48	1.1E-04	0.67	6.4E-09	0.51	3.4E-05
	cg23602058	17	48,154,061	*ITGA3*	Body	open sea	0.56	2.6E-06	0.35	5.4E-03	0.60	3.7E-07	0.65	1.6E-08	0.55	6.2E-06
	cg21767759	17	48,154,356	*ITGA3*	Body	open sea	0.52	1.7E-05	0.35	6.9E-03	0.56	4.1E-06	0.61	2.8E-07	0.57	1.9E-06
**IGR9**	cg27665571	16	70,624,354	*intergenic*	IGR	open sea	-0.41	1.3E-03	-0.31	1.7E-02	-0.34	8.5E-03	-0.41	1.3E-03	-0.29	2.7E-02
	cg10485485	16	70,624,357	*intergenic*	IGR	open sea	-0.39	2.2E-03	-0.27	3.9E-02	-0.38	2.7E-03	-0.45	3.0E-04	-0.28	2.9E-02
	cg00636508	16	70,624,430	*intergenic*	IGR	open sea	-0.52	2.5E-05	-0.27	3.8E-02	-0.54	1.0E-05	-0.58	1.3E-06	-0.46	2.3E-04

**IGR7**	cg15644756	12	69,198,743	*intergenic*	IGR	shelf	0.51	3.4E-05	0.34	7.5E-03	0.54	7.5E-06	0.60	3.4E-07	0.52	2.3E-05
	cg13322954	12	69,198,953	*intergenic*	IGR	shelf	0.52	2.2E-05	0.38	2.9E-03	0.58	1.0E-06	0.61	2.4E-07	0.55	5.6E-06
	cg10635494	12	69,199,037	*intergenic*	IGR	shelf	0.54	7.7E-06	0.36	4.7E-03	0.57	1.7E-06	0.61	2.0E-07	0.50	4.1E-05
**AGRN**	cg01150641	1	976,168	*AGRN*	Body	island	0.43	6.0E-04	0.28	2.9E-02	0.43	6.6E-04	0.57	2.5E-06	0.52	2.2E-05
	cg23625715	1	976,172	*AGRN*	Body	island	0.44	3.7E-04	0.28	3.1E-02	0.46	2.1E-04	0.57	2.0E-06	0.49	8.2E-05
	cg26222311	1	976,227	*AGRN*	Body	island	0.49	8.1E-05	0.30	1.9E-02	0.51	3.5E-05	0.60	3.9E-07	0.46	1.9E-04
**ARHGEF25**	cg22610645	12	58,003,898	*ARHGEF25*	TSS1500	island	0.49	8.1E-05	0.34	7.3E-03	0.54	8.6E-06	0.63	9.1E-08	0.56	3.2E-06
	cg11211563	12	58,003,905	*ARHGEF25*	TSS1500	island	0.49	7.8E-05	0.34	8.5E-03	0.55	5.5E-06	0.62	1.2E-07	0.51	3.8E-05
	cg15409097	12	58,003,925	*ARHGEF25*	TSS1500	island	0.53	1.5E-05	0.30	2.1E-02	0.63	6.6E-08	0.60	3.5E-07	0.51	3.1E-05
**TINAGL1**	cg15120085	1	32,041,898	*TINAGL1*	TSS200	open sea	-0.42	9.6E-04	-0.30	1.9E-02	-0.46	2.0E-04	-0.49	6.0E-05	-0.43	6.8E-04
	cg00541683	1	32,041,933	*TINAGL1*	TSS200	open sea	-0.36	5.3E-03	-0.26	4.1E-02	-0.37	4.0E-03	-0.46	2.4E-04	-0.25	5.2E-02
	cg15079885	1	32,041,940	*TINAGL1*	TSS200	open sea	-0.47	1.3E-04	-0.36	4.9E-03	-0.53	1.4E-05	-0.56	3.5E-06	-0.39	2.2E-03
	cg22855405	1	32,042,037	*TINAGL1*	TSS200	open sea	-0.48	1.0E-04	-0.33	9.0E-03	-0.54	1.0E-05	-0.61	2.1E-07	-0.44	4.7E-04
	cg24873592	1	32,042,092	*TINAGL1*	5'UTR	open sea	-0.41	1.2E-03	-0.26	4.2E-02	-0.46	2.1E-04	-0.48	1.2E-04	-0.38	2.5E-03
	cg18107144	1	32,042,157	*TINAGL1*	5'UTR	open sea	-0.48	9.0E-05	-0.33	8.9E-03	-0.53	1.1E-05	-0.61	2.4E-07	-0.48	9.1E-05
	cg14869028	1	32,042,161	*TINAGL1*	5'UTR	open sea	-0.49	7.4E-05	-0.35	6.3E-03	-0.53	1.5E-05	-0.57	1.7E-06	-0.45	3.6E-04
**CASZ1**	cg26522708	1	10,839,450	*CASZ1*	5'UTR	open sea	-0.50	5.7E-05	-0.29	2.4E-02	-0.51	3.1E-05	-0.62	1.7E-07	-0.55	6.6E-06
	cg26689934	1	10,839,502	*CASZ1*	5'UTR	open sea	-0.42	8.5E-04	-0.22	8.8E-02	-0.39	2.1E-03	-0.52	1.8E-05	-0.56	3.3E-06
	cg25463742	1	10,839,574	*CASZ1*	5'UTR	open sea	-0.42	8.6E-04	-0.20	1.2E-01	-0.39	1.9E-03	-0.53	1.4E-05	-0.46	2.4E-04
QPRT	cg00572323	16	29,706,151	*QPRT*	Body	shore	0.52	2.2E-05	0.37	3.2E-03	0.63	5.6E-08	0.45	3.6E-04	0.51	3.8E-05
	cg03488456	16	29,706,275	*QPRT*	Body	island	0.56	2.6E-06	0.42	9.8E-04	0.64	3.9E-08	0.50	5.4E-05	0.53	1.2E-05
	cg01468711	16	29,706,291	*QPRT*	Body	island	0.56	3.2E-06	0.38	2.4E-03	0.63	7.5E-08	0.48	8.8E-05	0.52	2.1E-05
**PWWP2B**	cg11579421	10	134,211,857	*PWWP2B*	Body	shore	-0.51	2.8E-05	-0.31	1.6E-02	-0.58	9.5E-07	-0.51	2.9E-05	-0.54	9.2E-06
	cg25303150	10	134,211,874	*PWWP2B*	Body	shore	-0.49	7.7E-05	-0.32	1.3E-02	-0.58	1.4E-06	-0.51	3.3E-05	-0.54	7.8E-06
	cg24085039	10	134,211,908	*PWWP2B*	Body	shore	-0.50	4.1E-05	-0.36	4.6E-03	-0.56	3.8E-06	-0.52	1.7E-05	-0.52	2.5E-05
BACH2	cg10365984	6	91,004,430	*BACH2*	5'UTR	shore	0.41	1.0E-03	0.25	5.5E-02	0.45	3.2E-04	0.48	1.2E-04	0.43	5.6E-04
	cg09745430	6	91,004,452	*BACH2*	5'UTR	shore	0.36	5.1E-03	0.24	6.5E-02	0.43	5.7E-04	0.49	6.4E-05	0.47	1.6E-04

	cg24667115	6	91,004,482	*BACH2*	5'UTR	shore	0.36	4.9E-03	0.13	3.1E-01	0.36	4.7E-03	0.47	1.6E-04	0.40	1.8E-03
Network 2																
PEMT	cg21605540	17	17,485,823	*PEMT*	Body	open sea	0.45	3.0E-04	0.32	1.2E-02	0.57	2.3E-06	0.39	2.0E-03	0.37	3.3E-03
	cg02094018	17	17,485,934	*PEMT*	Body	open sea	0.43	5.9E-04	0.36	4.2E-03	0.58	1.2E-06	0.32	1.2E-02	0.35	5.5E-03
	cg02295973	17	17,486,105	*PEMT*	Body	open sea	0.34	7.9E-03	0.30	2.2E-02	0.45	3.1E-04	0.29	2.6E-02	0.33	9.7E-03
LBX2-AS1	cg02100410	2	74,731,354	*LBX2-AS1*	Body	shore	0.40	1.8E-03	0.36	5.4E-03	0.52	1.9E-05	0.33	1.1E-02	0.36	5.3E-03
	cg25251459	2	74,731,371	*LBX2-AS1*	Body	shore	0.34	7.0E-03	0.32	1.2E-02	0.53	1.4E-05	0.30	1.9E-02	0.29	2.5E-02
	cg13407169	2	74,731,413	*LBX2-AS1*	Body	shore	0.43	5.9E-04	0.32	1.2E-02	0.57	2.3E-06	0.36	4.7E-03	0.30	2.0E-02
RBP5_2	cg14672128	12	7,280,912	*RBP5*	Body	open sea	0.33	9.5E-03	0.33	9.7E-03	0.49	7.8E-05	0.27	3.9E-02	0.34	8.7E-03
	cg12074585	12	7,280,958	*RBP5*	Body	open sea	0.37	3.2E-03	0.33	9.4E-03	0.53	1.1E-05	0.26	4.5E-02	0.31	1.6E-02
	cg20315995	12	7,280,971	*RBP5*	Body	open sea	0.37	3.2E-03	0.33	9.2E-03	0.50	4.1E-05	0.26	4.5E-02	0.30	1.9E-02
	cg24441911	12	7,280,988	*RBP5*	Body	open sea	0.36	4.5E-03	0.34	8.0E-03	0.51	3.0E-05	0.29	2.7E-02	0.37	3.4E-03
	cg24319651	12	7,281,343	*RBP5*	1stExon	open sea	0.30	1.9E-02	0.34	8.6E-03	0.47	1.7E-04	0.24	6.4E-02	0.35	6.8E-03
FTCD	cg04413147	21	47,575,134	*FTCD*	Body	open sea	0.34	8.8E-03	0.26	4.6E-02	0.48	9.6E-05	0.25	5.2E-02	0.32	1.3E-02
	cg10394047	21	47,575,416	*FTCD*	1stExon	open sea	0.35	5.6E-03	0.33	1.1E-02	0.46	2.4E-04	0.28	2.8E-02	0.37	3.3E-03
	cg09436823	21	47,575,498	*FTCD*	TSS200	open sea	0.35	6.4E-03	0.33	1.1E-02	0.49	6.4E-05	0.33	8.9E-03	0.29	2.6E-02
	cg18024037	21	47,575,504	*FTCD*	TSS200	open sea	0.42	9.2E-04	0.33	1.0E-02	0.54	9.4E-06	0.32	1.2E-02	0.33	9.5E-03
	cg25322086	21	47,575,547	*FTCD*	TSS200	open sea	0.41	1.1E-03	0.31	1.6E-02	0.51	2.6E-05	0.32	1.4E-02	0.35	6.6E-03
ABCG5;AB	cg01186613	2	44,065,003	*ABCG5;ABCG8*	Body	open sea	0.40	1.4E-03	0.38	3.1E-03	0.50	5.7E-05	0.27	3.8E-02	0.33	9.4E-03
CG8	cg16451365	2	44,065,056	*ABCG5;ABCG8*	Body	open sea	0.30	2.1E-02	0.28	2.8E-02	0.44	5.0E-04	0.31	1.5E-02	0.30	2.1E-02
	cg00705576	2	44,065,259	*ABCG5;ABCG8*	Body	open sea	0.27	3.8E-02	0.27	3.9E-02	0.41	1.0E-03	0.29	2.2E-02	0.28	3.1E-02
	cg11467440	2	44,065,278	*ABCG5;ABCG8*	Body	open sea	0.30	1.9E-02	0.27	4.0E-02	0.42	8.7E-04	0.18	1.6E-01	0.21	1.1E-01
	cg13341470	2	44,065,332	*ABCG5;ABCG8*	Body	open sea	0.34	7.2E-03	0.33	9.0E-03	0.47	1.6E-04	0.27	3.6E-02	0.33	9.1E-03
	cg11113753	2	44,065,383	*ABCG5;ABCG8*	Body	open sea	0.38	3.0E-03	0.33	9.3E-03	0.50	3.9E-05	0.27	3.8E-02	0.33	9.3E-03
	cg18281102	2	44,065,550	*ABCG5;ABCG8*	Body	open sea	0.38	2.7E-03	0.34	8.9E-03	0.52	2.5E-05	0.32	1.2E-02	0.31	1.4E-02
	cg00009421	2	44,065,571	*ABCG5;ABCG8*	Body	open sea	0.33	9.4E-03	0.38	3.1E-03	0.47	1.5E-04	0.30	1.9E-02	0.33	1.1E-02
	cg04680150	2	44,065,627	*ABCG5;ABCG8*	Body	open sea	0.43	6.7E-04	0.33	1.1E-02	0.53	1.3E-05	0.29	2.4E-02	0.37	3.6E-03

	cg00459909	2	44,065,720	*ABCG5;ABCG8*	TSS1500	open sea	0.33	9.5E-03	0.32	1.3E-02	0.47	1.5E-04	0.31	1.5E-02	0.35	6.0E-03
	cg20926720	2	44,065,725	*ABCG5;ABCG8*	TSS1500	open sea	0.35	5.5E-03	0.31	1.6E-02	0.46	2.4E-04	0.29	2.7E-02	0.29	2.3E-02
	cg07681696	2	44,065,858	*ABCG5;ABCG8*	5'UTR	open sea	0.38	2.8E-03	0.32	1.4E-02	0.49	6.6E-05	0.27	4.0E-02	0.37	3.6E-03
	cg08453096	2	44,065,893	*ABCG5;ABCG8*	5'UTR	open sea	0.40	1.3E-03	0.28	3.1E-02	0.47	1.6E-04	0.26	4.6E-02	0.33	1.0E-02
	cg03157395	2	44,065,964	*ABCG5;ABCG8*	TSS200	open sea	0.39	2.1E-03	0.24	6.8E-02	0.51	3.2E-05	0.29	2.7E-02	0.40	1.5E-03
	cg25781162	2	44,065,996	*ABCG5;ABCG8*	TSS200	open sea	0.35	6.3E-03	0.25	5.4E-02	0.50	4.4E-05	0.33	9.8E-03	0.33	1.1E-02
	cg05864261	2	44,066,030	*ABCG5;ABCG8*	TSS200	open sea	0.40	1.4E-03	0.26	4.6E-02	0.55	5.8E-06	0.28	3.2E-02	0.21	1.0E-01
SGK2	cg06796271	20	42,187,587	*SGK2*	TSS200	open sea	0.36	4.9E-03	0.24	6.1E-02	0.51	3.2E-05	0.27	4.0E-02	0.28	3.3E-02
	cg01021952	20	42,187,632	*SGK2*	TSS200	open sea	0.35	5.5E-03	0.29	2.3E-02	0.44	3.9E-04	0.25	5.4E-02	0.34	8.1E-03
	cg06600331	20	42,187,650	*SGK2*	TSS200	open sea	0.36	4.9E-03	0.29	2.3E-02	0.49	7.3E-05	0.27	3.9E-02	0.28	2.9E-02
	cg17611262	20	42,187,750	*SGK2*	1stExon	open sea	0.37	3.7E-03	0.30	2.0E-02	0.53	1.4E-05	0.30	2.1E-02	0.37	4.0E-03
	cg17463527	20	42,187,837	*SGK2*	5'UTR	open sea	0.40	1.7E-03	0.27	3.5E-02	0.51	3.1E-05	0.26	4.2E-02	0.25	5.5E-02
RBP5_1	cg00294025	12	7,276,360	*RBP5*	3'UTR	open sea	0.47	1.7E-04	0.36	5.3E-03	0.58	1.3E-06	0.46	2.3E-04	0.43	5.4E-04
	cg10993460	12	7,276,482	*RBP5*	3'UTR	open sea	0.37	3.6E-03	0.30	1.9E-02	0.49	8.6E-05	0.35	6.6E-03	0.36	5.0E-03
	cg16959747	12	7,276,714	*RBP5*	3'UTR	open sea	0.40	1.6E-03	0.31	1.8E-02	0.51	2.6E-05	0.41	1.2E-03	0.26	4.1E-02
APOC4	cg17769836	19	45,445,437	*APOC4*	TSS200	open sea	0.36	4.5E-03	0.27	3.7E-02	0.50	5.1E-05	0.24	6.8E-02	0.28	2.9E-02
	cg04401876	19	45,445,449	*APOC4*	TSS200	open sea	0.39	2.2E-03	0.29	2.5E-02	0.54	6.9E-06	0.28	2.7E-02	0.25	5.3E-02
	cg04347059	19	45,445,486	*APOC4*	TSS200	open sea	0.36	4.7E-03	0.26	4.8E-02	0.49	7.8E-05	0.27	3.7E-02	0.26	4.1E-02
	cg02912790	19	45,445,491	*APOC4*	TSS200	open sea	0.32	1.3E-02	0.24	6.6E-02	0.50	5.5E-05	0.33	9.5E-03	0.26	4.8E-02
	cg27353824	19	45,445,521	*APOC4*	5'UTR	open sea	0.36	5.3E-03	0.29	2.5E-02	0.47	1.5E-04	0.30	1.9E-02	0.37	4.0E-03
	cg25017250	19	45,445,693	*APOC4*	Body	open sea	0.39	2.3E-03	0.28	2.8E-02	0.46	2.0E-04	0.22	8.6E-02	0.22	9.0E-02
RGS12_3	cg09912079	4	3,409,828	*RGS12*	Body	open sea	0.42	8.8E-04	0.34	7.1E-03	0.59	6.1E-07	0.29	2.6E-02	0.35	6.4E-03
	cg25447202	4	3,409,885	*RGS12*	Body	open sea	0.40	1.7E-03	0.31	1.5E-02	0.50	5.6E-05	0.28	3.1E-02	0.36	4.6E-03
	cg06353485	4	3,409,927	*RGS12*	Body	open sea	0.43	5.8E-04	0.37	3.2E-03	0.57	2.5E-06	0.35	5.7E-03	0.38	2.8E-03
	cg11463380	4	3,410,693	*RGS12*	Body	shelf	0.50	5.1E-05	0.40	1.6E-03	0.61	1.9E-07	0.41	1.2E-03	0.47	1.3E-04
CHID1	cg07659663	11	914,329	*CHID1*	5'UTR	shelf	0.54	9.2E-06	0.27	3.8E-02	0.64	5.0E-08	0.50	4.0E-05	0.33	9.1E-03
	cg23202388	11	914,849	*CHID1*	1stExon	shelf	0.51	3.8E-05	0.35	6.4E-03	0.58	1.0E-06	0.38	3.1E-03	0.38	2.9E-03

	cg10668614	11	915,163	*CHID1*	TSS200	shelf	0.45	3.5E-04	0.32	1.2E-02	0.57	1.6E-06	0.34	8.3E-03	0.34	8.0E-03
	cg06639440	11	915,170	*CHID1*	TSS200	shelf	0.39	2.1E-03	0.32	1.2E-02	0.53	1.2E-05	0.39	2.3E-03	0.31	1.7E-02
	cg16402814	11	915,227	*CHID1*	TSS200	shelf	0.47	1.3E-04	0.38	3.0E-03	0.58	1.0E-06	0.31	1.7E-02	0.31	1.6E-02
	cg18205787	11	915,239	*CHID1*	TSS200	shelf	0.44	4.0E-04	0.31	1.6E-02	0.58	1.4E-06	0.34	7.2E-03	0.33	1.0E-02
	cg23449764	11	915,337	*CHID1*	TSS1500	shelf	0.42	9.1E-04	0.25	5.9E-02	0.54	1.0E-05	0.29	2.7E-02	0.29	2.6E-02
	cg14166189	11	915,440	*CHID1*	TSS1500	shelf	0.43	6.2E-04	0.29	2.5E-02	0.58	1.2E-06	0.39	2.0E-03	0.28	3.2E-02
HGFAC	cg23922755	4	3,443,656	*HGFAC*	TSS200	open sea	0.33	1.0E-02	0.22	9.5E-02	0.45	3.4E-04	0.27	3.5E-02	0.20	1.2E-01
	cg26955579	4	3,443,683	*HGFAC*	TSS200	open sea	0.36	4.7E-03	0.26	4.2E-02	0.47	1.7E-04	0.30	1.8E-02	0.31	1.4E-02
	cg20533530	4	3,443,694	*HGFAC*	TSS200	open sea	0.39	1.8E-03	0.32	1.3E-02	0.55	6.7E-06	0.29	2.4E-02	0.31	1.7E-02
	cg17322505	4	3,443,735	*HGFAC*	1stExon	open sea	0.29	2.2E-02	0.24	6.4E-02	0.48	1.2E-04	0.18	1.7E-01	0.34	7.9E-03
	cg07364841	4	3,444,147	*HGFAC*	Body	open sea	0.40	1.4E-03	0.36	4.8E-03	0.56	3.7E-06	0.28	2.8E-02	0.30	1.9E-02
GCK	cg17650622	7	44,198,725	*GCK*	1stExon	open sea	0.37	3.3E-03	0.31	1.4E-02	0.53	1.7E-05	0.32	1.3E-02	0.26	4.5E-02
	cg21504093	7	44,199,012	*GCK*	Body	open sea	0.37	3.5E-03	0.32	1.2E-02	0.51	2.9E-05	0.24	6.7E-02	0.27	3.5E-02
	cg20035206	7	44,199,106	*GCK*	Body	open sea	0.33	1.0E-02	0.26	4.3E-02	0.52	1.9E-05	0.29	2.3E-02	0.29	2.3E-02
	cg03345391	7	44,199,266	*GCK*	Body	open sea	-0.08	5.3E-01	-0.22	9.4E-02	-0.18	1.7E-01	-0.38	3.0E-03	-0.02	9.0E-01
	cg21987356	7	44,199,597	*GCK*	Body	open sea	0.32	1.4E-02	0.28	2.8E-02	0.46	2.1E-04	0.25	5.5E-02	0.29	2.4E-02
PGLYRP2	cg17915429	19	15,590,069	*PGLYRP2*	Body	open sea	0.32	1.1E-02	0.32	1.3E-02	0.45	3.1E-04	0.28	2.9E-02	0.31	1.6E-02
	cg17752089	19	15,590,308	*PGLYRP2*	5'UTR	open sea	0.34	8.7E-03	0.27	3.5E-02	0.46	2.5E-04	0.30	1.8E-02	0.30	2.1E-02
	cg09054960	19	15,590,328	*PGLYRP2*	TSS200	open sea	0.30	2.0E-02	0.32	1.3E-02	0.43	5.6E-04	0.32	1.2E-02	0.29	2.3E-02
	cg22310770	19	15,590,368	*PGLYRP2*	TSS200	open sea	0.33	1.1E-02	0.26	4.3E-02	0.50	5.6E-05	0.26	4.8E-02	0.29	2.2E-02
	cg07408456	19	15,590,532	*PGLYRP2*	TSS1500	open sea	0.34	7.7E-03	0.26	4.4E-02	0.48	1.3E-04	0.29	2.5E-02	0.31	1.8E-02
	cg17473673	19	15,590,570	*PGLYRP2*	TSS1500	open sea	0.48	9.3E-05	0.36	4.7E-03	0.53	1.2E-05	0.43	6.3E-04	0.40	1.4E-03
TBCD	cg01771850	17	80,847,137	*TBCD*	Body	open sea	0.35	6.6E-03	0.29	2.5E-02	0.47	1.6E-04	0.28	3.0E-02	0.35	6.1E-03
	cg05398905	17	80,847,209	*TBCD*	Body	open sea	0.41	1.0E-03	0.30	1.9E-02	0.53	1.2E-05	0.34	7.1E-03	0.32	1.3E-02
	cg03535099	17	80,847,270	*TBCD*	Body	open sea	0.37	3.8E-03	0.31	1.5E-02	0.48	9.5E-05	0.28	3.3E-02	0.28	3.2E-02
	cg09152949	17	80,847,497	*TBCD*	Body	open sea	0.54	8.1E-06	0.42	9.2E-04	0.58	1.2E-06	0.33	9.1E-03	0.40	1.5E-03
	cg21156912	17	80,847,546	*TBCD*	Body	open sea	0.43	5.8E-04	0.29	2.4E-02	0.52	2.0E-05	0.27	3.3E-02	0.36	4.5E-03

	cg19788754	17	80,847,662	*TBCD*	Body	open sea	0.42	7.4E-04	0.27	3.8E-02	0.50	4.3E-05	0.27	3.7E-02	0.27	3.9E-02
SLC7A5	cg03553613	16	87,879,502	*SLC7A5*	Body	open sea	0.40	1.7E-03	0.41	1.1E-03	0.49	6.5E-05	0.27	3.9E-02	0.16	2.1E-01
	cg04171052	16	87,879,581	*SLC7A5*	Body	open sea	0.41	1.1E-03	0.34	8.0E-03	0.53	1.5E-05	0.33	9.3E-03	0.36	4.9E-03
	cg26637881	16	87,879,698	*SLC7A5*	Body	open sea	0.38	3.0E-03	0.28	3.3E-02	0.48	8.7E-05	0.30	1.8E-02	0.34	7.8E-03
IRF7	cg18477816	11	612,588	*IRF7*	3'UTR	shore	0.46	2.2E-04	0.35	6.2E-03	0.59	7.5E-07	0.46	2.2E-04	0.37	4.1E-03
	cg03755158	11	612,680	*IRF7*	Body	shore	0.34	7.3E-03	0.25	5.2E-02	0.55	6.2E-06	0.32	1.3E-02	0.25	4.9E-02
	cg27271532	11	612,762	*IRF7*	Body	shore	0.39	1.8E-03	0.30	2.1E-02	0.56	2.8E-06	0.35	6.0E-03	0.32	1.3E-02
	cg05309505	11	612,837	*IRF7*	Body	shore	0.43	6.0E-04	0.35	6.2E-03	0.58	1.0E-06	0.34	7.0E-03	0.29	2.6E-02
HNF4A	cg08314996	20	42,984,099	*HNF4A*	TSS1500	open sea	0.34	7.3E-03	0.27	3.7E-02	0.52	2.4E-05	0.23	7.4E-02	0.25	5.4E-02
	cg16121136	20	42,984,209	*HNF4A*	TSS1500	open sea	0.33	1.1E-02	0.27	3.8E-02	0.44	4.6E-04	0.24	6.3E-02	0.34	8.0E-03
	cg24084358	20	42,984,276	*HNF4A*	TSS200	open sea	0.29	2.7E-02	0.30	2.1E-02	0.47	1.4E-04	0.28	3.3E-02	0.29	2.3E-02
	cg06126829	20	42,984,320	*HNF4A*	TSS200	open sea	0.39	2.3E-03	0.26	4.8E-02	0.51	2.6E-05	0.31	1.5E-02	0.38	2.9E-03
	cg06640637	20	42,984,324	*HNF4A*	TSS200	open sea	0.40	1.7E-03	0.27	3.6E-02	0.51	3.8E-05	0.34	8.5E-03	0.31	1.7E-02
	cg20848979	20	42,984,338	*HNF4A*	TSS200	open sea	0.40	1.6E-03	0.30	2.1E-02	0.52	2.2E-05	0.29	2.4E-02	0.34	8.4E-03
	cg22958104	20	42,984,347	*HNF4A*	TSS200	open sea	0.35	6.3E-03	0.27	3.6E-02	0.47	1.5E-04	0.29	2.7E-02	0.35	5.8E-03
	cg16221969	20	42,984,394	*HNF4A*	TSS200	open sea	0.29	2.6E-02	0.24	6.9E-02	0.42	7.2E-04	0.23	8.1E-02	0.31	1.7E-02
	cg23792485	20	42,984,453	*HNF4A*	1stExon	open sea	0.39	2.2E-03	0.34	8.7E-03	0.52	1.7E-05	0.34	7.6E-03	0.32	1.3E-02
	cg21081369	20	42,984,579	*HNF4A*	Body	open sea	0.41	1.1E-03	0.31	1.7E-02	0.53	1.1E-05	0.31	1.7E-02	0.34	7.0E-03
	cg19717150	20	42,984,878	*HNF4A*	Body	open sea	0.28	2.8E-02	0.27	3.5E-02	0.47	1.6E-04	0.27	3.8E-02	0.29	2.4E-02
MIR192;MIR194-2	cg05560951	11	64,658,226	*MIR192;MIR194-2*	IGR	open sea	0.54	9.7E-06	0.36	5.0E-03	0.58	1.3E-06	0.51	3.6E-05	0.47	1.5E-04
	cg02258444	11	64,658,622	*MIR192;MIR194-2*	Body	shelf	0.41	1.3E-03	0.28	3.1E-02	0.55	4.6E-06	0.32	1.3E-02	0.31	1.7E-02
	cg27083891	11	64,658,726	*MIR192;MIR194-2*	TSS200	shelf	0.41	1.2E-03	0.31	1.6E-02	0.55	4.9E-06	0.35	6.2E-03	0.32	1.3E-02
	cg09349409	11	64,658,765	*MIR192;MIR194-2*	TSS200	shelf	0.36	4.2E-03	0.27	3.6E-02	0.51	3.8E-05	0.29	2.7E-02	0.33	1.1E-02
	cg18262830	11	64,658,819	*MIR192;MIR194-2*	TSS200	shelf	0.37	3.3E-03	0.27	3.8E-02	0.52	1.7E-05	0.36	4.6E-03	0.27	3.8E-02
	cg24803202	11	64,658,903	*MIR192;MIR194-2*	Body	shelf	0.37	3.8E-03	0.30	2.0E-02	0.50	4.3E-05	0.31	1.7E-02	0.33	1.1E-02
	cg08432452	11	64,658,936	*MIR192;MIR194-2*	TSS1500	shelf	0.32	1.3E-02	0.24	6.9E-02	0.47	1.4E-04	0.29	2.4E-02	0.34	7.6E-03
	cg00589493	11	64,658,940	*MIR192;MIR194-2*	TSS1500	shelf	0.35	5.8E-03	0.28	3.0E-02	0.49	7.2E-05	0.29	2.5E-02	0.33	1.1E-02

	cg13092487	11	64,658,946	*MIR192;MIR194-2*	TSS1500	shelf	0.34	8.4E-03	0.28	3.2E-02	0.48	1.1E-04	0.31	1.6E-02	0.34	7.6E-03
	cg24154336	11	64,659,044	*MIR192;MIR194-2*	TSS1500	shelf	0.32	1.2E-02	0.31	1.5E-02	0.43	5.7E-04	0.27	3.5E-02	0.23	7.2E-02
	cg00400165	11	64,659,060	*MIR192;MIR194-2*	TSS1500	shelf	0.37	3.6E-03	0.25	5.5E-02	0.46	2.2E-04	0.27	3.8E-02	0.33	1.0E-02
	cg00376448	11	64,659,065	*MIR192;MIR194-2*	TSS1500	shelf	0.30	2.0E-02	0.28	3.0E-02	0.46	2.6E-04	0.28	3.3E-02	0.35	6.8E-03
	cg02494703	11	64,659,387	*MIR192;MIR194-2*	TSS1500	shelf	0.42	8.7E-04	0.30	1.8E-02	0.55	5.2E-06	0.28	2.8E-02	0.29	2.7E-02
MIR629;TLE3	cg05185738	15	70,371,992	*MIR629;TLE3*	TSS200	open sea	0.34	7.7E-03	0.34	8.5E-03	0.48	1.2E-04	0.37	3.2E-03	0.32	1.1E-02
	cg17972789	15	70,372,105	*MIR629;TLE3*	Body	open sea	0.31	1.6E-02	0.37	3.7E-03	0.49	7.2E-05	0.39	2.2E-03	0.32	1.3E-02
	cg13912196	15	70,372,127	*MIR629;TLE3*	Body	open sea	0.38	2.8E-03	0.34	7.2E-03	0.49	6.2E-05	0.39	1.9E-03	0.34	8.5E-03
	cg02852421	15	70,372,614	*MIR629;TLE3*	Body	open sea	0.33	1.0E-02	0.34	8.2E-03	0.50	5.3E-05	0.37	3.8E-03	0.35	6.4E-03
NCOA4	cg06098215	10	51,575,702	*NCOA4*	5'UTR	shelf	0.36	5.2E-03	0.35	6.6E-03	0.48	1.2E-04	0.35	5.8E-03	0.24	7.0E-02
	cg16814786	10	51,575,763	*NCOA4*	5'UTR	shelf	0.32	1.2E-02	0.34	7.0E-03	0.42	7.2E-04	0.39	2.1E-03	0.25	5.3E-02
	cg01315067	10	51,576,232	*NCOA4*	TSS200	shelf	0.30	2.0E-02	0.30	2.2E-02	0.49	7.2E-05	0.26	4.5E-02	0.24	6.7E-02
	cg00302587	10	51,576,241	*NCOA4*	TSS200	shelf	0.35	6.5E-03	0.32	1.3E-02	0.46	2.1E-04	0.29	2.7E-02	0.26	4.1E-02
	cg20166027	10	51,576,452	*NCOA4*	1stExon	shelf	0.40	1.8E-03	0.31	1.5E-02	0.48	1.2E-04	0.34	7.3E-03	0.34	8.6E-03
LIMS2	cg13099839	2	128,422,113	*LIMS2*	Body	island	0.41	9.8E-04	0.35	5.6E-03	0.48	1.2E-04	0.38	2.6E-03	0.45	3.6E-04
	cg16944093	2	128,422,120	*LIMS2*	Body	island	0.41	1.3E-03	0.36	4.5E-03	0.48	1.0E-04	0.50	5.2E-05	0.37	3.8E-03
	cg10661054	2	128,422,179	*LIMS2*	Body	island	0.48	1.2E-04	0.23	8.3E-02	0.52	1.9E-05	0.55	4.4E-06	0.46	2.5E-04
	cg19426955	2	128,422,224	*LIMS2*	Body	shore	0.49	8.4E-05	0.29	2.4E-02	0.55	5.8E-06	0.31	1.7E-02	0.38	3.0E-03
	cg11535366	2	128,422,284	*LIMS2*	Body	shore	0.48	1.2E-04	0.33	9.3E-03	0.55	5.5E-06	0.30	2.2E-02	0.36	4.8E-03
	cg18044111	2	128,422,307	*LIMS2*	Body	shore	0.46	2.2E-04	0.34	8.7E-03	0.56	3.6E-06	0.33	1.1E-02	0.34	8.6E-03
	cg21339084	2	128,422,433	*LIMS2*	Body	shore	0.44	4.1E-04	0.34	8.7E-03	0.53	1.2E-05	0.32	1.4E-02	0.39	2.3E-03
	cg14489933	2	128,422,619	*LIMS2*	Body	shore	0.38	2.8E-03	0.31	1.5E-02	0.52	1.8E-05	0.30	1.8E-02	0.32	1.3E-02
	cg19326543	2	128,422,715	*LIMS2*	Body	shore	0.43	5.6E-04	0.37	3.3E-03	0.55	5.0E-06	0.33	9.6E-03	0.29	2.3E-02
	cg15781838	2	128,422,717	*LIMS2*	Body	shore	0.35	5.6E-03	0.34	7.9E-03	0.48	9.5E-05	0.30	2.1E-02	0.34	8.0E-03
FABP1	cg19910382	2	88,427,561	*FABP1*	1stExon	open sea	0.36	5.1E-03	0.29	2.2E-02	0.48	1.0E-04	0.31	1.6E-02	0.27	3.6E-02
	cg19217130	2	88,427,581	*FABP1*	TSS200	open sea	0.37	3.7E-03	0.33	9.6E-03	0.49	5.9E-05	0.33	1.0E-02	0.24	6.7E-02
	cg24046616	2	88,427,630	*FABP1*	TSS200	open sea	0.28	3.0E-02	0.29	2.7E-02	0.37	3.7E-03	0.28	2.9E-02	0.23	7.4E-02

	cg24933157	2	88,427,637	*FABP1*	TSS200	open sea	0.38	2.6E-03	0.33	9.8E-03	0.47	1.4E-04	0.27	3.4E-02	0.29	2.6E-02
WWP2	cg16439003	16	69,975,149	*WWP2*	3'UTR	open sea	0.31	1.6E-02	0.21	1.0E-01	0.33	1.1E-02	0.14	2.8E-01	0.32	1.3E-02
	cg03840678	16	69,975,320	*WWP2*	3'UTR	open sea	0.39	2.4E-03	0.31	1.7E-02	0.56	3.1E-06	0.30	1.9E-02	0.33	1.1E-02
	cg26481896	16	69,975,405	*WWP2*	3'UTR	open sea	0.42	7.2E-04	0.33	1.1E-02	0.57	2.6E-06	0.31	1.7E-02	0.35	6.9E-03
	cg27186420	16	69,975,590	*WWP2*	3'UTR	open sea	0.44	4.8E-04	0.30	1.8E-02	0.54	7.1E-06	0.26	4.6E-02	0.31	1.6E-02
	cg26821137	16	69,976,040	*WWP2*	IGR	open sea	0.40	1.6E-03	0.30	2.0E-02	0.53	1.2E-05	0.31	1.5E-02	0.30	1.9E-02
	cg00549064	16	69,976,228	*WWP2*	IGR	open sea	0.45	2.6E-04	0.33	1.0E-02	0.58	1.3E-06	0.43	5.9E-04	0.34	8.4E-03
NUPR1	cg08818284	16	28,548,873	*NUPR1*	3'UTR	open sea	0.31	1.5E-02	0.30	2.2E-02	0.47	1.7E-04	0.24	6.2E-02	0.19	1.4E-01
	cg06723057	16	28,549,923	*NUPR1*	Body	open sea	0.34	7.4E-03	0.28	3.0E-02	0.42	8.4E-04	0.27	4.0E-02	0.23	7.6E-02
	cg05590982	16	28,550,171	*NUPR1*	1stExon	open sea	0.36	4.4E-03	0.29	2.4E-02	0.53	1.2E-05	0.33	1.0E-02	0.28	3.3E-02
	cg04492847	16	28,550,525	*NUPR1*	TSS200	open sea	0.37	3.9E-03	0.30	2.1E-02	0.50	4.2E-05	0.43	5.7E-04	0.25	5.5E-02
	cg06288570	16	28,550,567	*NUPR1*	TSS200	open sea	0.36	4.6E-03	0.26	4.5E-02	0.54	9.6E-06	0.23	7.8E-02	0.28	3.2E-02
	cg15149645	16	28,550,619	*NUPR1*	TSS200	open sea	0.42	7.5E-04	0.29	2.5E-02	0.53	1.5E-05	0.35	6.5E-03	0.32	1.4E-02
	cg01542023	16	28,550,637	*NUPR1*	TSS200	open sea	0.34	7.3E-03	0.31	1.5E-02	0.49	6.4E-05	0.44	4.0E-04	0.21	1.1E-01
CUX1	cg19346623	7	101,901,663	*CUX1*	Body	open sea	0.39	1.9E-03	0.28	2.9E-02	0.50	5.1E-05	0.32	1.3E-02	0.30	1.9E-02
	cg02611466	7	101,901,707	*CUX1*	Body	open sea	0.40	1.5E-03	0.30	1.9E-02	0.53	1.2E-05	0.35	6.6E-03	0.39	2.0E-03
	cg04151469	7	101,901,764	*CUX1*	Body	open sea	0.47	1.4E-04	0.36	4.4E-03	0.61	1.8E-07	0.43	6.2E-04	0.38	2.7E-03
PROC	cg25457027	2	128,175,179	*PROC*	TSS1500	shore	0.40	1.6E-03	0.32	1.2E-02	0.46	1.9E-04	0.39	1.9E-03	0.32	1.2E-02
	cg10021288	2	128,175,891	*PROC*	TSS200	shelf	0.29	2.4E-02	0.29	2.4E-02	0.46	2.3E-04	0.26	4.7E-02	0.24	6.7E-02
	cg22856114	2	128,175,905	*PROC*	TSS200	shelf	0.28	3.1E-02	0.31	1.8E-02	0.47	1.5E-04	0.26	4.1E-02	0.27	3.5E-02
	cg06038358	2	128,176,007	*PROC*	5'UTR	shelf	0.34	7.7E-03	0.28	2.8E-02	0.50	4.5E-05	0.29	2.2E-02	0.26	4.9E-02
	cg26718585	2	128,176,454	*PROC*	5'UTR	shelf	0.30	2.1E-02	0.27	3.6E-02	0.43	6.2E-04	0.21	1.1E-01	0.33	1.1E-02
	cg11143063	2	128,177,475	*PROC*	5'UTR	shelf	0.32	1.4E-02	0.27	4.1E-02	0.46	1.9E-04	0.24	6.3E-02	0.29	2.7E-02
IGR2	cg11847956	5	173,198,447	*intergenic*	IGR	open sea	0.38	2.7E-03	0.31	1.8E-02	0.50	5.0E-05	0.37	3.4E-03	0.31	1.6E-02
	cg11212451	5	173,198,508	*intergenic*	IGR	open sea	0.38	3.0E-03	0.29	2.4E-02	0.53	1.6E-05	0.42	9.0E-04	0.27	3.5E-02
	cg10818423	5	173,198,602	*intergenic*	IGR	open sea	0.40	1.5E-03	0.33	1.1E-02	0.56	3.5E-06	0.42	8.1E-04	0.34	7.1E-03
C12orf74	cg04614923	12	93,096,622	*C12orf74*	TSS1500	open sea	0.06	6.5E-01	0.14	3.0E-01	0.17	1.9E-01	0.00	9.8E-01	0.05	6.8E-01

	cg12755471	12	93,096,780	*C12orf74*	TSS200	open sea	0.42	9.3E-04	0.35	6.4E-03	0.55	4.3E-06	0.28	2.7E-02	0.34	7.6E-03
	cg27576271	12	93,096,811	*C12orf74*	TSS200	open sea	0.44	4.1E-04	0.34	7.5E-03	0.54	8.6E-06	0.32	1.2E-02	0.36	5.2E-03
	cg26383838	12	93,096,819	*C12orf74*	TSS200	open sea	0.39	1.8E-03	0.27	3.4E-02	0.48	1.0E-04	0.28	2.8E-02	0.31	1.7E-02
	cg25382573	12	93,096,844	*C12orf74*	5'UTR	open sea	0.43	6.0E-04	0.27	3.8E-02	0.55	6.5E-06	0.32	1.4E-02	0.29	2.5E-02
IGR6	cg19299755	11	116,706,051	*intergenic*	IGR	shore	0.39	2.1E-03	0.39	1.9E-03	0.54	7.3E-06	0.38	2.5E-03	0.29	2.2E-02
	cg23193059	11	116,706,090	*intergenic*	IGR	shore	0.46	2.2E-04	0.36	4.7E-03	0.55	4.3E-06	0.40	1.7E-03	0.36	4.2E-03
	cg03044513	11	116,706,153	*intergenic*	IGR	shore	0.37	3.3E-03	0.36	4.5E-03	0.50	5.2E-05	0.31	1.5E-02	0.33	9.7E-03
RTKN	cg03295928	2	74,669,349	*RTKN*	TSS1500	island	0.40	1.4E-03	0.24	7.0E-02	0.44	4.7E-04	0.33	1.1E-02	0.32	1.2E-02
	cg14481208	2	74,669,375	*RTKN*	TSS1500	island	0.44	4.1E-04	0.39	2.3E-03	0.56	2.7E-06	0.37	3.3E-03	0.36	5.2E-03
	cg00658082	2	74,669,380	*RTKN*	TSS1500	island	0.37	3.4E-03	0.32	1.2E-02	0.48	1.2E-04	0.34	7.4E-03	0.35	6.1E-03
	cg26090072	2	74,669,387	*RTKN*	TSS1500	island	0.37	3.9E-03	0.31	1.5E-02	0.45	2.9E-04	0.30	1.8E-02	0.35	5.7E-03
FOXP2	cg01306563	7	114,055,074	*FOXP2*	1stExon	open sea	0.30	1.8E-02	0.27	3.7E-02	0.48	1.1E-04	0.31	1.6E-02	0.19	1.5E-01
	cg18546840	7	114,055,123	*FOXP2*	1stExon	open sea	0.26	4.6E-02	0.30	1.8E-02	0.37	3.3E-03	0.35	6.8E-03	0.20	1.2E-01
	cg24786986	7	114,055,133	*FOXP2*	1stExon	open sea	0.38	2.6E-03	0.28	2.8E-02	0.47	1.7E-04	0.34	7.5E-03	0.22	9.8E-02
	cg18871253	7	114,055,137	*FOXP2*	1stExon	open sea	0.34	8.6E-03	0.26	4.1E-02	0.51	2.8E-05	0.43	6.9E-04	0.33	1.0E-02
	cg19655952	7	114,055,204	*FOXP2*	1stExon	open sea	0.37	4.0E-03	0.26	4.3E-02	0.45	2.7E-04	0.40	1.7E-03	0.37	4.1E-03
	cg02211646	7	114,055,210	*FOXP2*	1stExon	open sea	0.41	1.0E-03	0.30	1.9E-02	0.49	8.4E-05	0.36	5.2E-03	0.32	1.2E-02
IGR3	cg08614082	6	158,375,684	*intergenic*	IGR	open sea	0.37	3.3E-03	0.30	2.2E-02	0.49	6.8E-05	0.41	1.2E-03	0.26	4.6E-02
	cg27359374	6		*intergenic*	IGR	open sea	0.45	3.5E-04	0.29	2.3E-02	0.49	7.4E-05	0.40	1.6E-03	0.31	1.8E-02
	cg11051752	6	158,375,814	*intergenic*	IGR	open sea	0.42	8.8E-04	0.33	1.1E-02	0.54	8.4E-06	0.34	8.1E-03	0.33	9.6E-03
CLU	cg08594681	8	27,468,684	*CLU*	1stExon	shelf	0.32	1.2E-02	0.20	1.4E-01	0.39	2.0E-03	0.28	3.2E-02	0.26	4.5E-02
	cg22313574	8	27,468,981	*CLU*	TSS200	shelf	0.43	6.6E-04	0.31	1.6E-02	0.50	5.7E-05	0.29	2.4E-02	0.36	4.9E-03
	cg14917244	8	27,469,001	*CLU*	TSS200	shelf	0.43	5.8E-04	0.29	2.4E-02	0.47	1.4E-04	0.39	2.3E-03	0.40	1.6E-03
	cg12729838	8	27,469,186	*CLU*	1stExon	shelf	0.35	6.4E-03	0.26	4.6E-02	0.42	9.2E-04	0.30	1.9E-02	0.27	3.8E-02
	cg11783834	8	27,469,331	*CLU*	Body	shelf	0.38	2.7E-03	0.29	2.3E-02	0.50	5.3E-05	0.29	2.3E-02	0.36	4.2E-03
	cg13488078	8	27,469,338	*CLU*	Body	shelf	0.39	1.8E-03	0.28	3.0E-02	0.49	6.5E-05	0.29	2.4E-02	0.34	7.3E-03
	cg00359590	8	27,469,673	*CLU*	TSS1500	shelf	0.34	8.3E-03	0.31	1.7E-02	0.46	2.2E-04	0.33	9.8E-03	0.34	8.6E-03

	cg19442470	8	27,470,225	*CLU*	TSS1500	shore	0.38	3.1E-03	0.32	1.3E-02	0.49	7.2E-05	0.33	1.1E-02	0.23	7.1E-02
DYNC1I1	cg09136052	7	95,546,508	*DYNC1I1*	Body	open sea	0.49	7.6E-05	0.35	6.9E-03	0.59	9.0E-07	0.38	2.7E-03	0.34	7.2E-03
	cg18450582	7	95,546,539	*DYNC1I1*	Body	open sea	0.44	4.2E-04	0.30	1.8E-02	0.65	1.4E-08	0.43	5.4E-04	0.27	3.4E-02
	cg23975973	7	95,546,556	*DYNC1I1*	Body	open sea	0.41	1.0E-03	0.39	2.3E-03	0.60	5.1E-07	0.45	2.8E-04	0.33	1.0E-02

DMRs in networks 1 and 2 observed in both the Japanese and American NAFLD cohorts. DMRs were sorted according to the number of edges connected to DMRs in the Japanese NAFLD cohort. In network 1, 23 DMRs were observed in the control group and are represented in bold.

Supplementary Table 4. Correlation between gene expression and CpG methylation levels in 62 DMRs associated with NAFLD progression.

DMR		Annotated	CpG feature		Correlation efficiency		Meta-analysis	
(node)	Probe	genes	Location	Island	Japanese (n = 56)	American (n = 44)	p.DSL	p.OP
Network 1								
**ZBTB38**	cg06137072	*ZBTB38*	5'UTR	open sea	-0.23	-0.23	**1.1E-02**	**7.5E-03**
	cg13029400	*ZBTB38*	5'UTR	open sea	-0.18	-0.27	**1.4E-02**	**9.4E-03**
	cg08360599	*ZBTB38*	5'UTR	open sea	-0.23	-0.24	**1.0E-02**	**6.6E-03**
	cg21370924	*ZBTB38*	5'UTR	open sea	-0.25	-0.26	**5.7E-03**	**3.1E-03**
	cg21474062	*ZBTB38*	5'UTR	open sea	-0.20	-0.30	**8.3E-03**	**5.0E-03**
**C2CD4D**	cg15015892	*C2CD4D*	Body	island	Low	NA	NA	NA
	cg05021743	*C2CD4D*	Body	island	Low	NA	NA	NA
	cg10781408	*C2CD4D*	Body	island	Low	NA	NA	NA
	cg04296699	*C2CD4D*	Body	island	Low	NA	NA	NA
**GPR56**	cg01410801	*GPR56*	TSS1500	open sea	-0.78	-0.73	**4.1E-22**	**4.5E-71**
	cg25645462	*GPR56*	TSS1500	open sea	-0.77	-0.68	**6.6E-20**	**7.2E-56**
	cg00550797	*GPR56*	TSS200	open sea	-0.79	-0.71	**6.0E-22**	**1.1E-68**
	cg16630572	*GPR56*	5'UTR	open sea	-0.79	-0.72	**8.4E-23**	**4.7E-75**
**FMN1**	cg15175581	*FMN1*	TSS200	open sea	-0.38	-0.09	**4.9E-02**	**3.9E-03**
	cg09347959	*FMN1*	TSS200	open sea	-0.37	-0.09	**4.7E-02**	**4.0E-03**
	cg17454592	*FMN1*	TSS1500	open sea	-0.37	-0.19	**1.8E-03**	**7.9E-04**
**SLC22A20**	cg02675946	*SLC22A20*	Body	shore	Low	NA	NA	NA
	cg27428304	*SLC22A20*	Body	island	Low	NA	NA	NA
	cg23930334	*SLC22A20*	Body	island	Low	NA	NA	NA
**TLE3**	cg20820622	*TLE3*	Body	open sea	-0.37	-0.31	**2.8E-04**	**5.6E-05**
	cg22537334	*TLE3*	Body	open sea	-0.34	-0.23	**1.7E-03**	**7.1E-04**
	cg21245875	*TLE3*	Body	open sea	-0.38	-0.29	**2.8E-04**	**5.8E-05**

**AGAP3**	cg25788549	*AGAP3*	Body	shore	-0.37	-0.23	**8.7E-04**	**3.0E-04**
	cg22169990	*AGAP3*	Body	shore	-0.40	-0.21	**6.6E-04**	**2.2E-04**
	cg21887193	*AGAP3*	Body	shore	-0.41	-0.24	**3.5E-04**	**8.9E-05**
**SULT2B1**	cg03039843	*SULT2B1*	TSS200	open sea	Low	Low	NA	NA
	cg23097961	*SULT2B1*	TSS200	open sea	Low	Low	NA	NA
	cg00698688	*SULT2B1*	1stExon	open sea	Low	Low	NA	NA
	cg08151612	*SULT2B1*	1stExon	open sea	Low	Low	NA	NA
	cg07543967	*SULT2B1*	1stExon	open sea	Low	Low	NA	NA
**LINC01550**	cg00263248	*LINC01550*	Body	open sea	-0.55	-0.23	**1.2E-02**	**4.5E-07**
	cg16062483	*LINC01550*	Body	open sea	-0.59	-0.18	**3.8E-02**	**3.5E-07**
	cg16278496	*LINC01550*	TSS200	open sea	-0.56	-0.16	**4.4E-02**	**3.8E-06**
	cg11798182	*LINC01550*	TSS200	open sea	-0.58	-0.15	5.3E-02	**2.5E-06**
	cg00034769	*LINC01550*	TSS200	open sea	-0.55	-0.05	1.1E-01	**8.9E-05**
**ALDH3B2**	cg20420868	*ALDH3B2*	1stExon	open sea	Low	Low	NA	NA
	cg07891457	*ALDH3B2*	1stExon	open sea	Low	Low	NA	NA
	cg18492926	*ALDH3B2*	5'UTR	open sea	Low	Low	NA	NA
	cg27123351	*ALDH3B2*	5'UTR	open sea	Low	Low	NA	NA
	cg24563501	*ALDH3B2*	TSS1500	open sea	Low	Low	NA	NA
**RHOD**	cg18043888	*RHOD*	3'UTR	island	0.02	0.08	3.3E-01	3.3E-01
	cg00023919	*RHOD*	3'UTR	island	0.04	0.15	2.0E-01	1.9E-01
	cg18407752	*RHOD*	3'UTR	shore	0.03	0.16	2.0E-01	2.0E-01
**SLC6A19**	cg02389859	*SLC6A19*	TSS200	island	-0.32	-0.47	**3.7E-05**	**2.8E-06**
	cg26948274	*SLC6A19*	TSS200	island	-0.35	-0.45	**2.7E-05**	**1.6E-06**
	cg17650028	*SLC6A19*	5'UTR	island	-0.37	-0.50	**3.8E-06**	**6.3E-08**
PHF13	cg00958217	*PHF13*	Body	shelf	-0.11	-0.04	2.3E-01	2.2E-01
	cg05377512	*PHF13*	Body	shelf	-0.12	-0.01	2.4E-01	2.4E-01
	cg15158876	*PHF13*	3'UTR	shelf	-0.11	-0.04	2.3E-01	2.2E-01

**ARRDC2**	cg05845141	*ARRDC2*	Body	island	-0.58	-0.40	**4.2E-06**	**1.0E-11**
	cg12965095	*ARRDC2*	Body	island	-0.58	-0.40	**2.3E-06**	**7.7E-12**
	cg12218406	*ARRDC2*	Body	island	-0.63	-0.47	**1.7E-08**	**3.3E-16**
**TIMP2**	cg07865166	*TIMP2*	Body	shore	-0.31	-0.03	1.1E-01	**2.9E-02**
	cg11342615	*TIMP2*	Body	shore	-0.38	-0.05	8.9E-02	**6.6E-03**
	cg05376904	*TIMP2*	Body	shore	-0.33	-0.04	9.7E-02	**1.7E-02**
**ITGA3**	cg00798317	*ITGA3*	Body	open sea	0.34	0.18	**3.2E-03**	**1.7E-03**
	cg23602058	*ITGA3*	Body	open sea	0.43	0.15	**2.3E-02**	**3.7E-04**
	cg21767759	*ITGA3*	Body	open sea	0.44	0.12	**4.4E-02**	**6.1E-04**
**AGRN**	cg01150641	*AGRN*	Body	island	0.44	0.27	**8.5E-05**	**1.2E-05**
	cg23625715	*AGRN*	Body	island	0.42	0.20	**2.7E-03**	**1.7E-04**
	cg26222311	*AGRN*	Body	island	0.44	0.23	**1.2E-03**	**4.6E-05**
**ARHGEF25**	cg22610645	*ARHGEF25*	TSS1500	island	0.42	-0.17	3.2E-01	5.1E-02
	cg11211563	*ARHGEF25*	TSS1500	island	0.42	-0.20	3.5E-01	7.1E-02
	cg15409097	*ARHGEF25*	TSS1500	island	0.49	-0.24	3.5E-01	**3.9E-02**
**TINAGL1**	cg15120085	*TINAGL1*	TSS200	open sea	-0.07	-0.03	3.0E-01	2.9E-01
	cg00541683	*TINAGL1*	TSS200	open sea	0.01	0.04	4.2E-01	4.2E-01
	cg15079885	*TINAGL1*	TSS200	open sea	-0.07	0.05	4.3E-01	4.3E-01
	cg22855405	*TINAGL1*	TSS200	open sea	-0.06	-0.03	3.2E-01	3.1E-01
	cg24873592	*TINAGL1*	5'UTR	open sea	-0.06	-0.11	2.2E-01	2.1E-01
	cg18107144	*TINAGL1*	5'UTR	open sea	-0.06	-0.08	2.5E-01	2.4E-01
	cg14869028	*TINAGL1*	5'UTR	open sea	-0.07	0.01	3.6E-01	3.5E-01
**CASZ1**	cg26522708	*CASZ1*	5'UTR	open sea	-0.12	-0.37	**3.1E-02**	**7.1E-03**
	cg26689934	*CASZ1*	5'UTR	open sea	-0.11	-0.29	**3.1E-02**	**2.4E-02**
	cg25463742	*CASZ1*	5'UTR	open sea	-0.09	-0.35	5.6E-02	**1.7E-02**
QPRT	cg00572323	*QPRT*	Body	shore	-0.33	-0.22	**2.5E-03**	**1.1E-03**
	cg03488456	*QPRT*	Body	island	-0.29	-0.31	**1.6E-03**	**5.8E-04**

	cg01468711	*QPRT*	Body	island	-0.29	-0.31	**1.6E-03**	**6.0E-04**
**PWWP2B**	cg11579421	*PWWP2B*	Body	shore	-0.27	-0.33	**1.6E-03**	**5.9E-04**
	cg25303150	*PWWP2B*	Body	shore	-0.34	-0.33	**3.6E-04**	**7.7E-05**
	cg24085039	*PWWP2B*	Body	shore	-0.33	-0.41	**9.6E-05**	**1.1E-05**
BACH2	cg10365984	*BACH2*	5'UTR	shore	0.08	0.00	3.3E-01	3.2E-01
	cg09745430	*BACH2*	5'UTR	shore	0.13	0.04	1.8E-01	1.8E-01
	cg24667115	*BACH2*	5'UTR	shore	0.00	-0.07	3.9E-01	3.9E-01
Network2								
PEMT	cg21605540	*PEMT*	Body	open sea	-0.20	0.03	2.0E-01	1.6E-01
	cg02094018	*PEMT*	Body	open sea	-0.25	0.10	3.1E-01	1.7E-01
	cg02295973	*PEMT*	Body	open sea	-0.20	0.03	2.1E-01	1.7E-01
LBX2-AS1	cg02100410	*LBX2-AS1*	Body	shore	-0.21	0.11	3.5E-01	2.4E-01
	cg25251459	*LBX2-AS1*	Body	shore	-0.30	0.03	2.0E-01	5.8E-02
	cg13407169	*LBX2-AS1*	Body	shore	-0.32	-0.01	1.3E-01	**2.8E-02**
RBP5_2	cg14672128	*RBP5*	Body	open sea	-0.41	-0.19	**2.7E-03**	**2.8E-04**
	cg12074585	*RBP5*	Body	open sea	-0.38	-0.04	9.9E-02	**7.4E-03**
	cg20315995	*RBP5*	Body	open sea	-0.41	-0.12	**3.4E-02**	**1.1E-03**
	cg24441911	*RBP5*	Body	open sea	-0.45	-0.19	**8.1E-03**	**8.1E-05**
	cg24319651	*RBP5*	1stExon	open sea	-0.34	-0.23	**1.7E-03**	**7.1E-04**
FTCD	cg04413147	*FTCD*	Body	open sea	-0.44	-0.19	**7.5E-03**	**1.1E-04**
	cg10394047	*FTCD*	1stExon	open sea	-0.45	-0.23	**1.3E-03**	**2.3E-05**
	cg09436823	*FTCD*	TSS200	open sea	-0.45	-0.14	**3.3E-02**	**2.4E-04**
	cg18024037	*FTCD*	TSS200	open sea	-0.51	-0.22	**8.3E-03**	**3.6E-06**
	cg25322086	*FTCD*	TSS200	open sea	-0.52	-0.14	**4.4E-02**	**2.6E-05**
ABCG5;ABCG8	cg01186613	*ABCG5;ABCG8*	Body	open sea	-0.06;-0.23	-0.20;-0.14	1.2E-01;**3.1E-02**	1.1E-01;**2.5E-02**
	cg16451365	*ABCG5;ABCG8*	Body	open sea	-0.22;-0.31	-0.23;-0.11	**1.2E-02**;**1.4E-02**	**8.0E-03**;**1.0E-02**
	cg00705576	*ABCG5;ABCG8*	Body	open sea	-0.16;-0.25	-0.16;-0.23	6.0E-02;**8.0E-03**	5.1E-02;**4.8E-03**

	cg11467440	*ABCG5;ABCG8*	Body	open sea	-0.16;-0.26	-0.26;-0.02	**2.2E-02**;9.9E-02	**1.6E-02;**5.3E-02
	cg13341470	*ABCG5;ABCG8*	Body	open sea	-0.16;-0.30	-0.09;-0.14	1.1E-01;**1.2E-02**	9.7E-02;**8.7E-03**
	cg11113753	*ABCG5;ABCG8*	Body	open sea	-0.16;-0.29	-0.18;-0.07	**4.9E-02**;**4.3E-02**	**4.0E-02**;**2.2E-02**
	cg18281102	*ABCG5;ABCG8*	Body	open sea	-0.19;-0.30	-0.20;-0.16	**3.0E-02**;**9.7E-03**	**2.3E-02**;**6.5E-03**
	cg00009421	*ABCG5;ABCG8*	Body	open sea	-0.13;-0.27	-0.20;-0.10	5.8E-02;**2.8E-02**	**4.9E-02**;**2.3E-02**
	cg04680150	*ABCG5;ABCG8*	Body	open sea	-0.15;-0.27	-0.04;-0.15	1.6E-01;**1.6E-02**	1.5E-01;**1.1E-02**
	cg00459909	*ABCG5;ABCG8*	TSS1500	open sea	-0.17;-0.21	-0.15;-0.05	5.7E-02;8.5E-02	**4.9E-02;**7.8E-02
	cg20926720	*ABCG5;ABCG8*	TSS1500	open sea	-0.15;-0.30	-0.28;-0.06	**2.2E-02**;6.2E-02	**1.6E-02**;**2.4E-02**
	cg07681696	*ABCG5;ABCG8*	5'UTR	open sea	-0.18;-0.27	-0.21;-0.10	**2.9E-02**;**2.7E-02**	**2.2E-02**;**2.2E-02**
	cg08453096	*ABCG5;ABCG8*	5'UTR	open sea	-0.16;-0.25	-0.12;0.01	8.5E-02;1.6E-01	7.6E-02;8.3E-02
	cg03157395	*ABCG5;ABCG8*	TSS200	open sea	-0.10;-0.19	-0.06;-0.07	2.0E-01;8.5E-02	2.0E-01;7.7E-02
	cg25781162	*ABCG5;ABCG8*	TSS200	open sea	-0.22;-0.27	-0.13;-0.09	**4.1E-02**;**3.2E-02**	**3.4E-02**;**2.6E-02**
	cg05864261	*ABCG5;ABCG8*	TSS200	open sea	-0.09;-0.23	-0.10;-0.16	1.8E-01;**2.5E-02**	1.7E-01;**1.9E-02**
SGK2	cg06796271	*SGK2*	TSS200	open sea	0.20	0.12	5.3E-02	**4.6E-02**
	cg01021952	*SGK2*	TSS200	open sea	0.20	0.17	**3.4E-02**	**2.7E-02**
	cg06600331	*SGK2*	TSS200	open sea	0.22	0.20	**2.0E-02**	**1.4E-02**
	cg17611262	*SGK2*	1stExon	open sea	0.21	0.19	**2.4E-02**	**1.8E-02**
	cg17463527	*SGK2*	5'UTR	open sea	0.22	0.14	**3.3E-02**	**2.6E-02**
RBP5_1	cg00294025	*RBP5*	3'UTR	open sea	-0.54	-0.38	**3.0E-07**	**7.6E-10**
	cg10993460	*RBP5*	3'UTR	open sea	-0.45	-0.25	**4.7E-04**	**1.6E-05**
	cg16959747	*RBP5*	3'UTR	open sea	-0.45	-0.17	**1.5E-02**	**1.2E-04**
APOC4	cg17769836	*APOC4*	TSS200	open sea	-0.32	-0.46	**4.2E-05**	**3.4E-06**
	cg04401876	*APOC4*	TSS200	open sea	-0.37	-0.46	**1.0E-05**	**3.1E-07**
	cg04347059	*APOC4*	TSS200	open sea	-0.37	-0.50	**4.2E-06**	**7.5E-08**
	cg02912790	*APOC4*	TSS200	open sea	-0.37	-0.46	**1.3E-05**	**4.7E-07**
	cg27353824	*APOC4*	5'UTR	open sea	-0.33	-0.45	**4.7E-05**	**3.9E-06**
	cg25017250	*APOC4*	Body	open sea	-0.33	-0.36	**2.6E-04**	**4.8E-05**

RGS12_3	cg09912079	*RGS12*	Body	open sea	-0.30	-0.36	**4.5E-04**	**1.1E-04**
	cg25447202	*RGS12*	Body	open sea	-0.31	-0.36	**4.0E-04**	**8.8E-05**
	cg06353485	*RGS12*	Body	open sea	-0.34	-0.22	**1.7E-03**	**7.1E-04**
	cg11463380	*RGS12*	Body	shelf	-0.45	-0.30	**4.4E-05**	**4.1E-06**
CHID1	cg07659663	*CHID1*	5'UTR	shelf	-0.54	-0.27	**3.3E-03**	**1.5E-07**
	cg23202388	*CHID1*	1stExon	shelf	-0.39	-0.07	7.4E-02	**3.8E-03**
	cg10668614	*CHID1*	TSS200	shelf	-0.37	-0.12	**2.3E-02**	**2.3E-03**
	cg06639440	*CHID1*	TSS200	shelf	-0.36	-0.10	**3.8E-02**	**5.0E-03**
	cg16402814	*CHID1*	TSS200	shelf	-0.36	-0.06	7.9E-02	**7.7E-03**
	cg18205787	*CHID1*	TSS200	shelf	-0.36	0.00	1.5E-01	**1.8E-02**
	cg23449764	*CHID1*	TSS1500	shelf	-0.34	-0.09	**3.9E-02**	**7.1E-03**
	cg14166189	*CHID1*	TSS1500	shelf	-0.38	-0.19	**2.0E-03**	**5.5E-04**
HGFAC	cg23922755	*HGFAC*	TSS200	open sea	-0.32	0.00	1.5E-01	**3.3E-02**
	cg26955579	*HGFAC*	TSS200	open sea	-0.35	0.02	1.7E-01	**2.5E-02**
	cg20533530	*HGFAC*	TSS200	open sea	-0.39	0.09	2.5E-01	**3.2E-02**
	cg17322505	*HGFAC*	1stExon	open sea	-0.35	-0.01	1.3E-01	**1.8E-02**
	cg07364841	*HGFAC*	Body	open sea	-0.42	-0.26	**1.8E-04**	**3.5E-05**
GCK	cg17650622	*GCK*	1stExon	open sea	-0.20	0.07	2.8E-01	2.0E-01
	cg21504093	*GCK*	Body	open sea	-0.16	0.13	4.4E-01	3.8E-01
	cg20035206	*GCK*	Body	open sea	-0.24	-0.02	9.0E-02	6.8E-02
	cg03345391	*GCK*	Body	open sea	0.05	0.05	3.1E-01	3.0E-01
	cg21987356	*GCK*	Body	open sea	-0.13	0.15	4.9E-01	4.6E-01
PGLYRP2	cg17915429	*PGLYRP2*	Body	open sea	-0.15	-0.10	1.0E-01	9.5E-02
	cg17752089	*PGLYRP2*	5'UTR	open sea	-0.27	0.01	1.6E-01	6.8E-02
	cg09054960	*PGLYRP2*	TSS200	open sea	-0.21	-0.16	**3.3E-02**	**2.7E-02**
	cg22310770	*PGLYRP2*	TSS200	open sea	-0.19	-0.07	8.9E-02	8.1E-02
	cg07408456	*PGLYRP2*	TSS1500	open sea	-0.12	-0.21	6.1E-02	5.2E-02

	cg17473673	*PGLYRP2*	TSS1500	open sea	-0.21	-0.04	8.9E-02	8.2E-02
TBCD	cg01771850	*TBCD*	Body	open sea	-0.23	-0.15	**2.8E-02**	**2.2E-02**
	cg05398905	*TBCD*	Body	open sea	-0.29	-0.17	**9.0E-03**	**5.8E-03**
	cg03535099	*TBCD*	Body	open sea	-0.29	-0.11	**1.6E-02**	**1.2E-02**
	cg09152949	*TBCD*	Body	open sea	-0.31	0.08	2.6E-01	7.6E-02
	cg21156912	*TBCD*	Body	open sea	-0.32	-0.12	**1.2E-02**	**7.5E-03**
	cg19788754	*TBCD*	Body	open sea	-0.29	-0.03	9.4E-02	**3.6E-02**
SLC7A5	cg03553613	*SLC7A5*	Body	open sea	-0.21	-0.22	**1.6E-02**	**1.1E-02**
	cg04171052	*SLC7A5*	Body	open sea	-0.26	0.03	1.9E-01	8.5E-02
	cg26637881	*SLC7A5*	Body	open sea	-0.24	0.04	2.2E-01	1.2E-01
IRF7	cg18477816	*IRF7*	3'UTR	shore	0.02	-0.14	3.2E-01	3.2E-01
	cg03755158	*IRF7*	Body	shore	0.18	-0.10	3.6E-01	2.8E-01
	cg27271532	*IRF7*	Body	shore	0.13	-0.15	5.0E-01	4.8E-01
	cg05309505	*IRF7*	Body	shore	0.20	-0.18	4.7E-01	3.7E-01
HNF4A	cg08314996	*HNF4A*	TSS1500	open sea	-0.26	-0.13	**2.4E-02**	**1.9E-02**
	cg16121136	*HNF4A*	TSS1500	open sea	-0.19	-0.14	**5.0E-02**	**4.2E-02**
	cg24084358	*HNF4A*	TSS200	open sea	-0.21	-0.15	**3.6E-02**	**2.9E-02**
	cg06126829	*HNF4A*	TSS200	open sea	-0.24	-0.20	**1.3E-02**	**8.9E-03**
	cg06640637	*HNF4A*	TSS200	open sea	-0.30	-0.21	**4.5E-03**	**2.4E-03**
	cg20848979	*HNF4A*	TSS200	open sea	-0.27	-0.18	**1.2E-02**	**8.1E-03**
	cg22958104	*HNF4A*	TSS200	open sea	-0.27	-0.14	**1.8E-02**	**1.3E-02**
	cg16221969	*HNF4A*	TSS200	open sea	-0.19	-0.16	**4.1E-02**	**3.3E-02**
	cg23792485	*HNF4A*	1stExon	open sea	-0.28	-0.28	**2.5E-03**	**1.1E-03**
	cg21081369	*HNF4A*	Body	open sea	-0.28	-0.24	**4.2E-03**	**2.1E-03**
	cg19717150	*HNF4A*	Body	open sea	-0.26	-0.30	**2.9E-03**	**1.3E-03**
MIR192;MIR194-2	cg05560951	*MIR192;MIR194-2*	IGR	open sea	NA	NA	NA	NA
	cg02258444	*MIR192;MIR194-2*	Body	shelf	NA	NA	NA	NA

	cg27083891	*MIR192;MIR194-2*	TSS200	shelf	NA	NA	NA	NA	
	cg09349409	*MIR192;MIR194-2*	TSS200	shelf	NA	NA	NA	NA	
	cg18262830	*MIR192;MIR194-2*	TSS200	shelf	NA	NA	NA	NA	
	cg24803202	*MIR192;MIR194-2*	Body	shelf	NA	NA	NA	NA	
	cg08432452	*MIR192;MIR194-2*	TSS1500	shelf	NA	NA	NA	NA	
	cg00589493	*MIR192;MIR194-2*	TSS1500	shelf	NA	NA	NA	NA	
	cg13092487	*MIR192;MIR194-2*	TSS1500	shelf	NA	NA	NA	NA	
	cg24154336	*MIR192;MIR194-2*	TSS1500	shelf	NA	NA	NA	NA	
	cg00400165	*MIR192;MIR194-2*	TSS1500	shelf	NA	NA	NA	NA	
	cg00376448	*MIR192;MIR194-2*	TSS1500	shelf	NA	NA	NA	NA	
	cg02494703	*MIR192;MIR194-2*	TSS1500	shelf	NA	NA	NA	NA	
MIR629;TLE3	cg05185738	*MIR629;TLE3*	TSS200	open sea	NA;0.31	NA;0.45	NA;**8.0E-05**	NA;**9.3E-06**	
	cg17972789	*MIR629;TLE3*	Body	open sea	NA;0.24	NA;0.45	NA;**6.8E-04**	NA;**2.1E-04**	
	cg13912196	*MIR629;TLE3*	Body	open sea	NA;0.31	NA;0.33	NA;**7.3E-04**	NA;**2.1E-04**	
	cg02852421	*MIR629;TLE3*	Body	open sea	NA;0.29	NA;0.44	NA;**1.7E-04**	NA;**2.8E-05**	
NCOA4	cg06098215	*NCOA4*	5'UTR	shelf	-0.09	-0.13	1.5E-01	1.4E-01	
	cg16814786	*NCOA4*	5'UTR	shelf	-0.03	-0.17	1.8E-01	1.7E-01	
	cg01315067	*NCOA4*	TSS200	shelf	-0.05	-0.07	2.8E-01	2.7E-01	
	cg00302587	*NCOA4*	TSS200	shelf	-0.04	-0.11	2.5E-01	2.4E-01	
	cg20166027	*NCOA4*	1stExon	shelf	-0.01	-0.16	2.3E-01	2.2E-01	
LIMS2	cg13099839	*LIMS2*	Body	island	-0.32	0.00	1.5E-01	**3.0E-02**	
	cg16944093	*LIMS2*	Body	island	-0.41	0.07	2.2E-01	**1.8E-02**	
	cg10661054	*LIMS2*	Body	island	-0.41	0.00	1.6E-01	**7.6E-03**	
	cg19426955	*LIMS2*	Body	shore	-0.38	0.04	2.0E-01	**2.0E-02**	
	cg11535366	*LIMS2*	Body	shore	-0.44	0.18	3.3E-01	**4.4E-02**	
	cg18044111	*LIMS2*	Body	shore	-0.45	0.14	2.9E-01	**2.3E-02**	
	cg21339084	*LIMS2*	Body	shore	-0.47	0.05	2.0E-01	**5.9E-03**	

	cg14489933	*LIMS2*	Body	shore	-0.43	0.09	2.4E-01	**1.8E-02**
	cg19326543	*LIMS2*	Body	shore	-0.46	0.13	2.8E-01	**1.9E-02**
	cg15781838	*LIMS2*	Body	shore	-0.40	0.04	2.0E-01	**1.5E-02**
FABP1	cg19910382	*FABP1*	1stExon	open sea	0.18	-0.34	3.8E-01	3.0E-01
	cg19217130	*FABP1*	TSS200	open sea	0.21	-0.37	3.9E-01	3.2E-01
	cg24046616	*FABP1*	TSS200	open sea	0.14	-0.29	3.7E-01	3.2E-01
	cg24933157	*FABP1*	TSS200	open sea	0.20	-0.25	4.7E-01	4.9E-01
WWP2	cg16439003	*WWP2*	3'UTR	open sea	0.06	0.04	3.1E-01	3.1E-01
	cg03840678	*WWP2*	3'UTR	open sea	0.01	0.07	3.5E-01	3.5E-01
	cg26481896	*WWP2*	3'UTR	open sea	0.06	-0.01	4.0E-01	4.0E-01
	cg27186420	*WWP2*	3'UTR	open sea	0.04	-0.04	4.6E-01	4.6E-01
	cg26821137	*WWP2*	IGR	open sea	-0.08	0.08	4.6E-01	4.6E-01
	cg00549064	*WWP2*	IGR	open sea	-0.09	0.07	4.4E-01	4.4E-01
NUPR1	cg08818284	*NUPR1*	3'UTR	open sea	-0.12	-0.15	9.8E-02	8.8E-02
	cg06723057	*NUPR1*	Body	open sea	-0.04	0.07	4.7E-01	4.7E-01
	cg05590982	*NUPR1*	1stExon	open sea	-0.19	-0.09	7.1E-02	6.3E-02
	cg04492847	*NUPR1*	TSS200	open sea	-0.11	-0.06	1.9E-01	1.8E-01
	cg06288570	*NUPR1*	TSS200	open sea	-0.17	-0.04	1.4E-01	1.3E-01
	cg15149645	*NUPR1*	TSS200	open sea	-0.13	0.00	2.4E-01	2.4E-01
	cg01542023	*NUPR1*	TSS200	open sea	-0.10	-0.08	1.9E-01	1.8E-01
CUX1	cg19346623	*CUX1*	Body	open sea	-0.32	0.28	4.6E-01	2.8E-01
	cg02611466	*CUX1*	Body	open sea	-0.29	0.35	4.6E-01	4.7E-01
	cg04151469	*CUX1*	Body	open sea	-0.38	0.32	4.6E-01	2.3E-01
PROC	cg25457027	*PROC*	TSS1500	shore	-0.48	0.01	1.6E-01	**2.5E-03**
	cg10021288	*PROC*	TSS200	shelf	-0.47	-0.24	**1.6E-03**	**8.7E-06**
	cg22856114	*PROC*	TSS200	shelf	-0.43	-0.20	**2.8E-03**	**1.2E-04**
	cg06038358	*PROC*	5'UTR	shelf	-0.40	-0.28	**2.0E-04**	**3.8E-05**

	cg26718585	*PROC*	5'UTR	shelf	-0.41	-0.03	1.2E-01	**5.3E-03**
	cg11143063	*PROC*	5'UTR	shelf	-0.43	-0.21	**2.6E-03**	**7.8E-05**
C12orf74	cg04614923	*C12orf74*	TSS1500	open sea	Low	Low	NA	NA
	cg12755471	*C12orf74*	TSS200	open sea	Low	Low	NA	NA
	cg27576271	*C12orf74*	TSS200	open sea	Low	Low	NA	NA
	cg26383838	*C12orf74*	TSS200	open sea	Low	Low	NA	NA
	cg25382573	*C12orf74*	5'UTR	open sea	Low	Low	NA	NA
RTKN	cg03295928	*RTKN*	TSS1500	island	-0.44	-0.23	**1.2E-03**	**3.6E-05**
	cg14481208	*RTKN*	TSS1500	island	-0.48	-0.23	**3.6E-03**	**7.4E-06**
	cg00658082	*RTKN*	TSS1500	island	-0.38	-0.04	1.0E-01	**7.4E-03**
	cg26090072	*RTKN*	TSS1500	island	-0.38	-0.02	1.3E-01	**1.0E-02**
FOXP2	cg01306563	*FOXP2*	1stExon	open sea	-0.35	-0.04	9.1E-02	**1.2E-02**
	cg18546840	*FOXP2*	1stExon	open sea	-0.23	-0.34	**3.0E-03**	**1.4E-03**
	cg24786986	*FOXP2*	1stExon	open sea	-0.26	-0.29	**3.4E-03**	**1.6E-03**
	cg18871253	*FOXP2*	1stExon	open sea	-0.27	-0.17	**1.3E-02**	**8.5E-03**
	cg19655952	*FOXP2*	1stExon	open sea	-0.36	-0.24	**1.1E-03**	**3.8E-04**
	cg02211646	*FOXP2*	1stExon	open sea	-0.33	-0.26	**1.3E-03**	**4.9E-04**
CLU	cg08594681	*CLU*	1stExon	shelf	-0.26	-0.17	**1.4E-02**	**1.0E-02**
	cg22313574	*CLU*	TSS200	shelf	-0.35	0.00	1.5E-01	**2.1E-02**
	cg14917244	*CLU*	TSS200	shelf	-0.26	-0.13	**2.1E-02**	**1.6E-02**
	cg12729838	*CLU*	1stExon	shelf	-0.18	0.00	1.5E-01	1.4E-01
	cg11783834	*CLU*	Body	shelf	-0.29	-0.08	**2.6E-02**	**2.0E-02**
	cg13488078	*CLU*	Body	shelf	-0.28	-0.12	**1.9E-02**	**1.4E-02**
	cg00359590	*CLU*	TSS1500	shelf	-0.30	-0.02	1.1E-01	**3.2E-02**
	cg19442470	*CLU*	TSS1500	shore	-0.30	-0.02	1.1E-01	**3.2E-02**
DYNC1I1	cg09136052	*DYNC1I1*	Body	open sea	-0.05	-0.11	2.3E-01	2.2E-01
	cg18450582	*DYNC1I1*	Body	open sea	-0.03	-0.08	3.1E-01	3.0E-01

	cg23975973	*DYNC1I1*	Body	open sea	-0.01	-0.05	4.1E-01	4.1E-01

DMRs in networks 1 and 2 observed in both the Japanese and American NAFLD cohorts. DMRs were sorted according to the number of edges connected to DMRs in the Japanese NAFLD cohort. In network 1, DMRs were observed in the control group and are represented in bold. p.DSL; *p*-values using the DerSimonian-Laird (DSL) random-effect meta-analytical approach with correlation coefficients as effect sizes, p.OP; p-values calculated using the Olkin-Pratt (OP) fixed-effect meta-analytical approach with correlation coefficients as effect sizes, NA; not available. Low; the expression levels were too low to analyze. p.DSL and p.OP <0.05 are represented in bold.

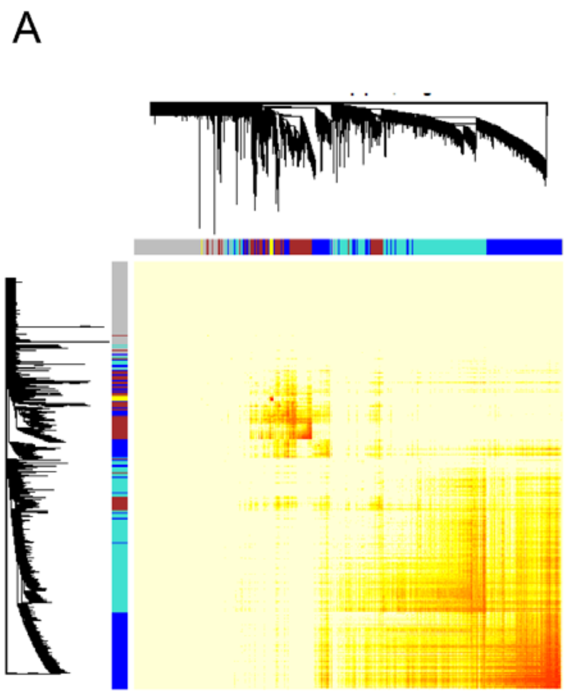

B

Topological overlap measure

		ZBTB38					C2CD4D				GPR56			
		cg06137072	cg13029400	cg08360599	cg21370924	cg21474062	cg15015892	cg05021743	cg10781408	cg04296699	cg01410801	cg25645462	cg00550797	cg16630572
ZBTB38	cg06137072						0.22	0.22	0.22	0.23	0.22	0.24	0.24	0.24
	cg13029400						0.20	0.20	0.20	0.20	0.20	0.21	0.21	0.21
	cg08360599						0.23	0.22	0.23	0.23	0.23	0.24	0.25	0.25
	cg21370924						0.26	0.25	0.26	0.26	0.26	0.26	0.27	0.27
	cg21474062						0.24	0.24	0.24	0.24	0.24	0.25	0.26	0.26
C2CD4D	cg15015892	0.22	0.20	0.23	0.26	0.24					0.22	0.21	0.22	0.22
	cg05021743	0.22	0.20	0.22	0.25	0.24					0.22	0.22	0.23	0.24
	cg10781408	0.22	0.20	0.23	0.26	0.24					0.22	0.20	0.21	0.22
	cg04296699	0.23	0.20	0.23	0.26	0.24					0.23	0.24	0.25	0.25
GPR56	cg01410801	0.22	0.20	0.23	0.26	0.24	0.22	0.22	0.22	0.23				
	cg25645462	0.24	0.21	0.24	0.26	0.25	0.21	0.22	0.20	0.24				
	cg00550797	0.24	0.21	0.25	0.27	0.26	0.22	0.23	0.21	0.25				
	cg16630572	0.24	0.21	0.25	0.27	0.26	0.22	0.24	0.22	0.25				

Average values of topological overlap measure in each DMR

	ZBTB38	C2CD4D	GPR56
ZBTB38	-	0.23	0.24
C2CD4D	0.23	-	0.22
GPR567	0.24	0.22	-

Supplementary Fig. 1. Co-methylation analysis of CpGs in the Japanese NAFLD cohort (A) and method for calculating the average values of topological overlap measures for the CpG matrix combining two different DMRs (B).

A. Heatmap of 3,683 CpGs observed in 610 DMRs (plotted in rows and columns). CpGs were clustered based on topological overlap measures, a measure of co-methylation interconnectedness, and were assigned to a module or group of densely co-methylated CpGs using Dynamic Tree Cut. Light colors represent low topological overlap; a progressively darker red color indicates increasing overlap. Blocks color-coded in turquoise, blue, brown, yellow, and grey along the diagonal correspond to modules. The dendrogram and module assignments are indicated on the left and on top. B. Method used for calculating the average values of topological overlap measures for the CpG matrix combining two different DMRs. For example, the average of 20 topological overlap measures in the matrix surrounded by the red lines is regarded as a representative measure of co-methylation interconnectedness between the ZBTB38 and C2CD4D DMRs.

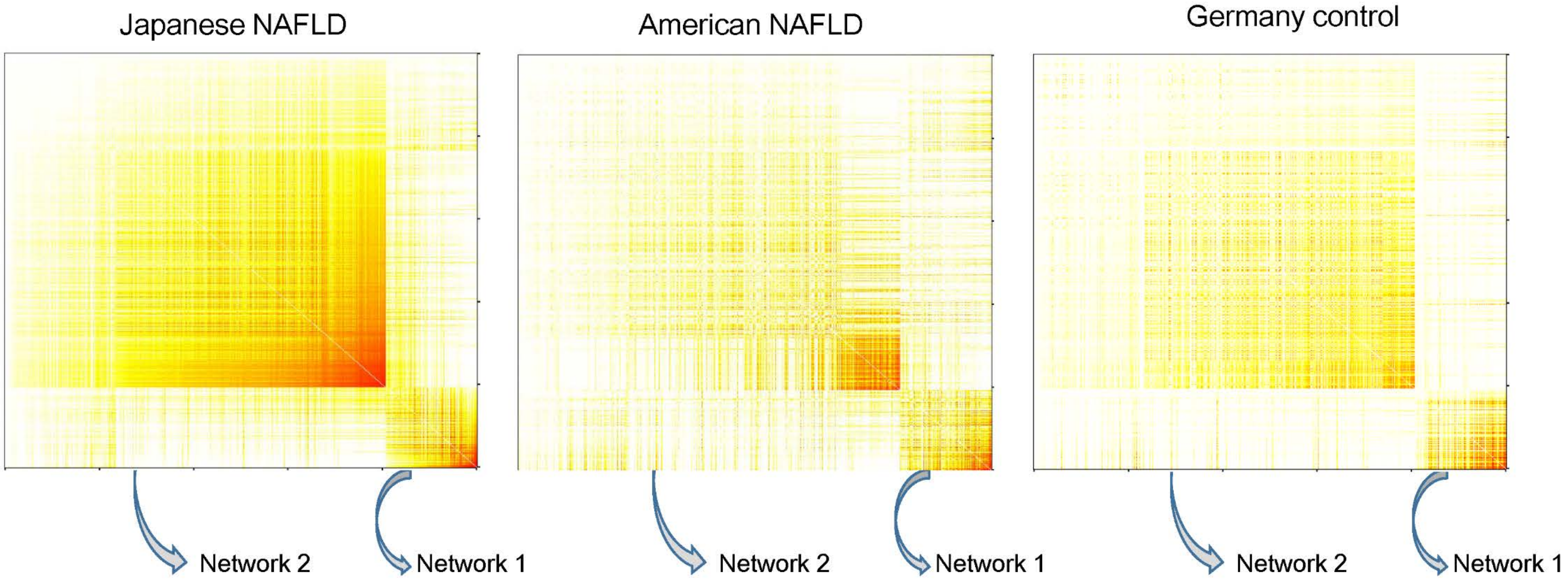

Supplementary Fig. 2. Heatmaps of 610 DMRs in the Japanese NAFLD, the American NAFLD, and German control groups.

The average values of topological overlap measures for the CpG matrix combining two different DMRs were calculated as described in the Methods section and Supplementary Fig. 1. Heatmaps of 610 DMRs were constructed using the Japanese and American NAFLD data and the German control group. The order of DMRs is the same in the three heatmaps. These indicated the existence of two putative networks (1 and 2).

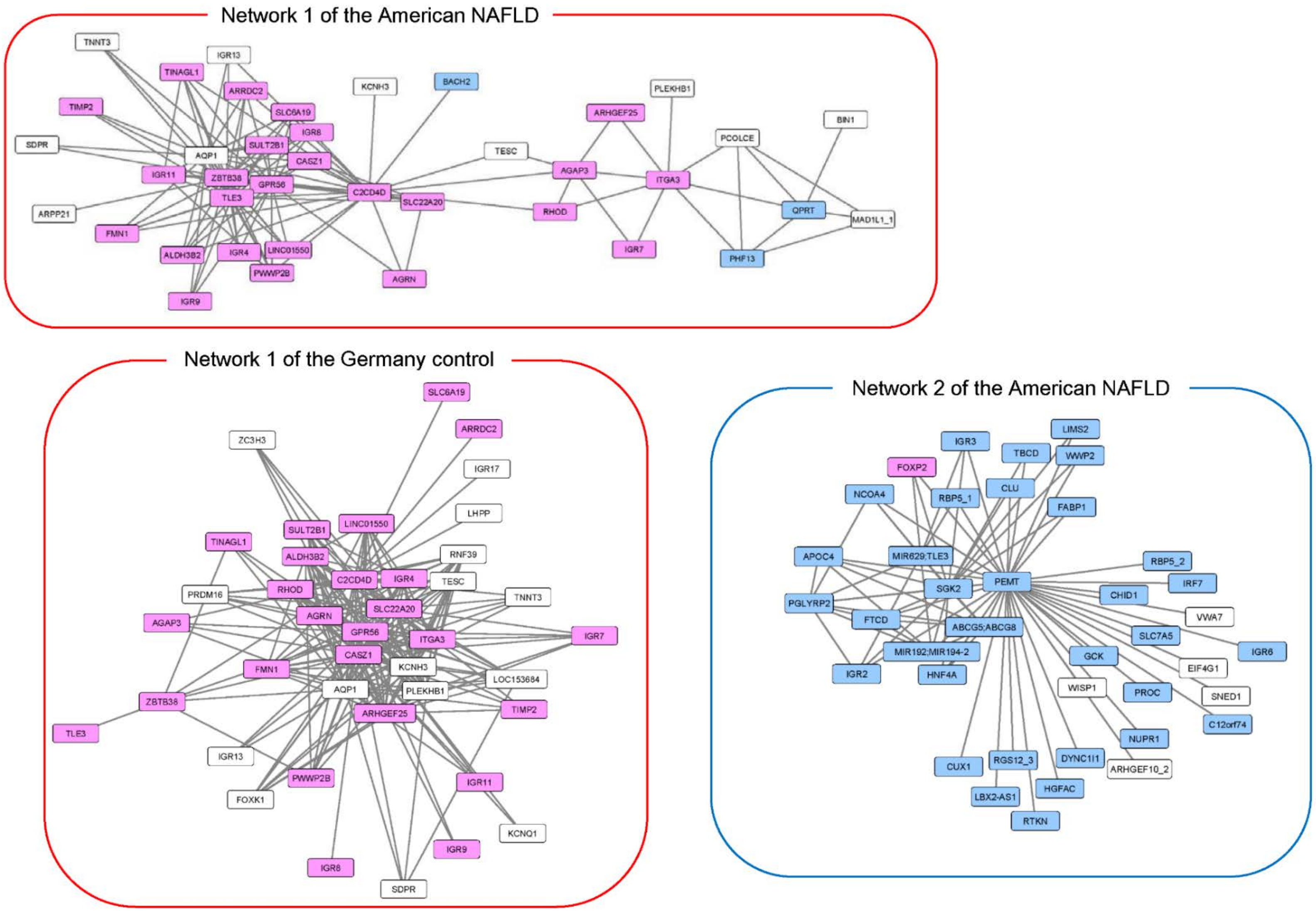

Supplementary Fig. 3. DMR networks in the American NAFLD and German control livers.

DMR networks in the American NAFLD and German control group were extracted as described in the Methods. Nodes (DMRs) identified in the Japanese and American NAFLD, and German control livers are indicated in pink. Nodes (DMRs) identified in the Japanese and American NAFLD cohorts, but not in German control livers, are indicated in blue.

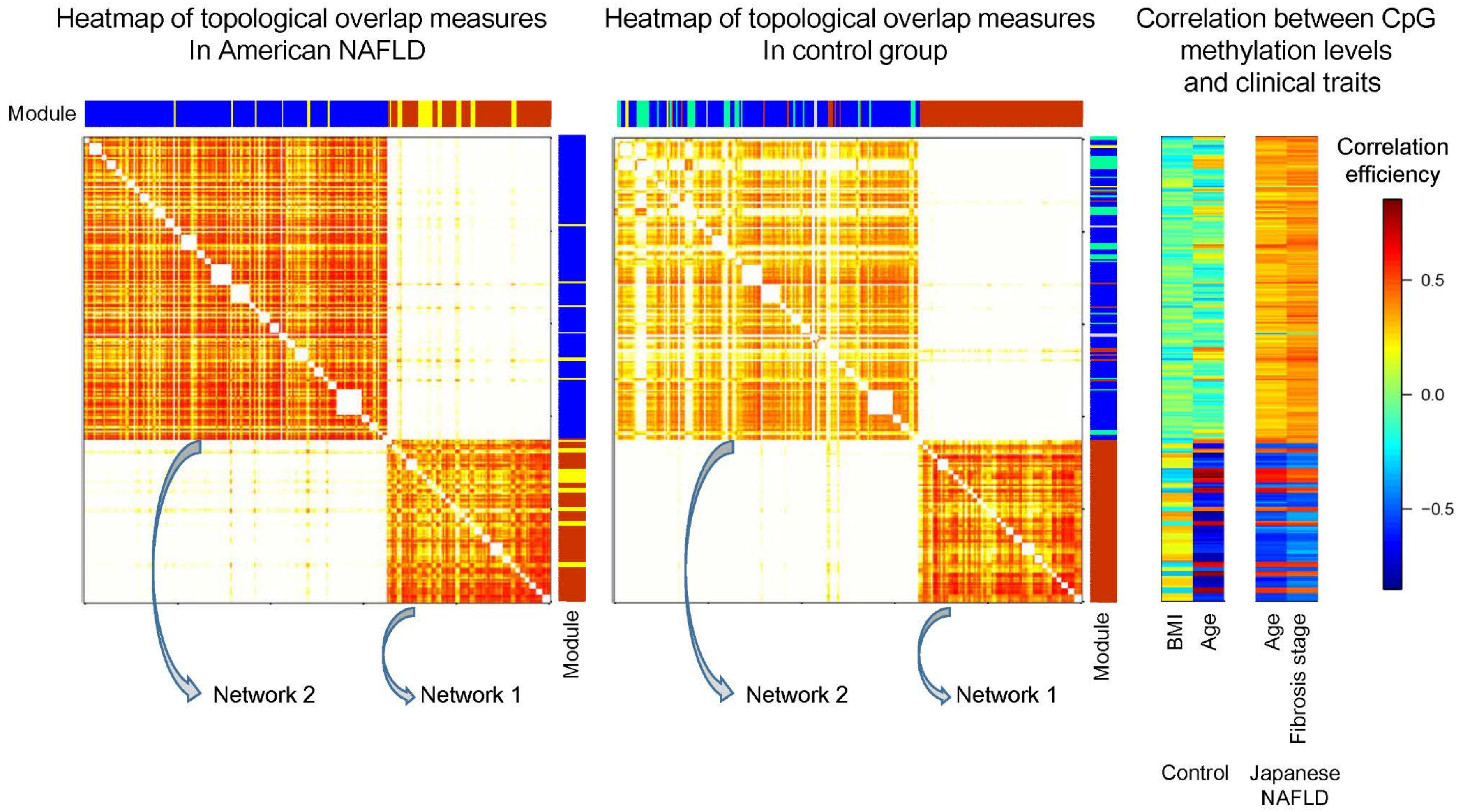

Supplementary Fig. 4. CpG heatmaps and correlations between CpG methylation levels and clinical traits.

The left panel (American NAFLD) and middle panel (German control group) are heatmaps of 294 CpGs in two DMR networks plotted in rows and columns. The heatmaps were generated using topological overlap measures, a measure of co-methylation interconnectedness between CpGs in the American NAFLD and control data. The order of CpGs was sorted by DMRs as shown in Supplementary Table 2. The topological overlap measures of CpGs present in the same DMRs were not used for generating the heatmap (white blocks). Light colors represent low topological overlap; a progressively darker red color indicates increasing overlap. Blocks color-coded in turquoise, blue, brown, yellow, and grey along the diagonal correspond to modules. In the right panel, the correlation efficiencies between the methylation levels of CpG sites and clinical traits are indicated. Each line corresponds to one CpG site in the heatmaps in the left and middle panels.

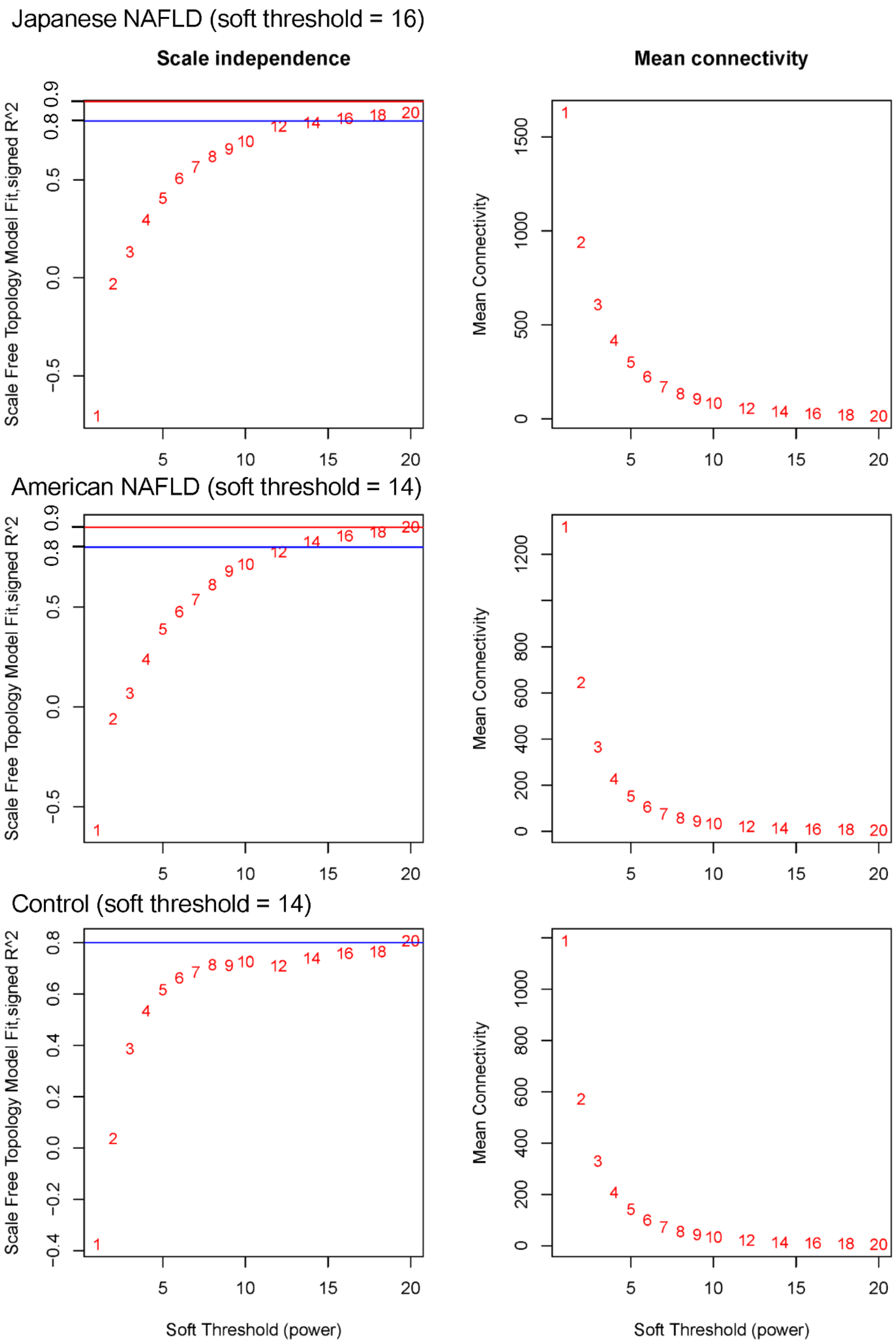

Supplementary Fig. 5. Network topology for various soft-thresholding powers.

The left panel shows the scale-free index and the right panel shows the mean connectivity at the various soft thresholding powers. The soft threshold was set at 16 for the Japanese NAFLD, 14 for the American NAFLD, and 14 for the German control.

Supplementary Methods

Codes for R packages "ChAMP"

#"myNorm.txt" file (β-values)

	GSM1201507	GSM1201509	GSM1201510	GSM1201512
cg00000029	0.38218471	0.469850605	0.420376462	0.342855286
cg00000108	0.438140651	0.472919312	0.512731986	0.539765061
cg00000109	0.887822366	0.882059515	0.906882642	0.956712582
cg00000165	0.193866048	0.234920635	0.264519109	0.117647059
cg00000236	0.844585868	0.881003963	0.864631623	0.900353898
cg00000289	0.754032958	0.739182058	0.752078944	0.717719016
cg00000292	0.72130195	0.700451109	0.772288718	0.797540322
cg00000321	0.274068702	0.287697374	0.30452099	0.279831533

#"myLoad.txt" file (C: mild NAFLD, T: advanced NAFLD)

	Sample_Name	Sample_Well	Sample_Plate	Sample_Group	Pool_ID	Array	Slide	Basename	filenames
GSM1201507	GSM1201507	NA	NA	C	NA	NA	NA	NA	NA
GSM1201509	GSM1201509	NA	NA	C	NA	NA	NA	NA	NA
GSM1201510	GSM1201510	NA	NA	C	NA	NA	NA	NA	NA
GSM1201512	GSM1201512	NA	NA	C	NA	NA	NA	NA	NA
GSM1201517	GSM1201517	NA	NA	C	NA	NA	NA	NA	NA
GSM1201519	GSM1201519	NA	NA	C	NA	NA	NA	NA	NA

```
library(ChAMP)
#Definition of differentially methylated CpG sites

in_f1<-"myNorm.txt"
myNorm<-read.table(in_f1,header=T,row.names=1,stringsAsFactors=F)
in_f2<-"myLoad.txt"
myLoad<-read.table(in_f2,header=TRUE,stringsAsFactors=F)

myDMP<-champ.DMP(beta = myNorm,
        pheno = myLoad$Sample_Group,
        compare.group = NULL,
        adjPVal = 0.05,
        adjust.method = "BH",
        arraytype = "450K")

out_f1<-"mydmp.txt"
write.table(myDMP,out_f1,sep="¥t",append=T,quote=F,row.names=T,col.names=T)

#Definition of DMR

myNorm2<-as.matrix(myNorm)
myLoad2<-as.factor(myLoad$Sample_Group)
```

```
myDMR<- champ.DMR (beta=myNorm2,
            pheno=myLoad2,
            arraytype="450K",
            method = "ProbeLasso",
            minProbes=3,
            adjPvalDmr=0.05,
            cores=3,
            meanLassoRadius=375,
            minDmrSep=1000,
            minDmrSize=50,
            adjPvalProbe=1.00,
            Rplot=T,
            PDFplot=T,
            resultsDir="./CHAMP_ProbeLasso/")

out_f1<-"mydmr_lasso.txt"
write.table(myDMR,out_f1,sep="¥t",append=T,quote=F,row.names=T,col.names=T)
```

Codes for R packages “WGCNA”

#"q2.csv" file (beta-value)

gene_id	GSM1201507	GSM1201509	GSM1201510	GSM1201512
cg01150641	0.169874445	0.243686681	0.273324938	0.671474323
cg23625715	0.021898017	0.046765682	0.067384652	0.490362293
cg26222311	0.049922559	0.132077001	0.118777852	0.51693061
cg07280242	0.743413083	0.755339667	0.757254123	0.701964285
cg13798621	0.766227265	0.76734701	0.808188523	0.710919032
cg13487474	0.817945273	0.834653591	0.831979373	0.794618823

#“ClinicalTraits.csv” file

sample	Fibrosis	sex
GSM1201506	1	1
GSM1201507	0	1
GSM1201508	1	1
GSM1201509	0	1

#“GeneAnnotation.csv” file

gene_id	Approved_Symbol_1	Entrez_Gene_ID	Approved_Symbol
cg01150641	AGRN	375790	cg01150641_AGRN_Body
cg23625715	AGRN	375790	cg23625715_AGRN_Body
cg26222311	AGRN	375790	cg26222311_AGRN_Body
cg07280242	ATAD3C	219293	cg07280242_ATAD3C_Body
cg13798621	ATAD3C	219293	cg13798621_ATAD3C_Body
cg13487474	ATAD3C	219293	cg13487474_ATAD3C_Body
cg23016632	ATAD3C	219293	cg23016632_ATAD3C_Body
cg26342907	NADK	65220	cg26342907_NADK_Body
cg11725331	NADK	65220	cg11725331_NADK_Body
cg10543797	NADK	65220	cg10543797_NADK_Body

```
library(WGCNA);
options(stringsAsFactors = FALSE);

femData = read.csv("q2.csv");

dim(femData);
names(femData);

datExpr0 = as.data.frame(t(femData[, -c(1:1)]));
names(datExpr0) = femData$gene_id;
rownames(datExpr0) = names(femData)[-c(1:1)];

gsg = goodSamplesGenes(datExpr0, verbose = 3);
gsg$allOK

#Plot the sample tree
sampleTree = hclust(dist(datExpr0), method = "average");
sizeGrWindow(12,9)
pdf(file = "sampleClustering_risk.pdf", width = 12, height = 9);
par(cex = 0.6);
par(mar = c(0,4,2,0))
plot(sampleTree, main = "Sample clustering to detect outliers", sub="", xlab="", cex.lab = 1.5, cex.axis = 1.5, cex.main = 2)
dev.off();

datExpr = datExpr0
nGenes = ncol(datExpr)
nSamples = nrow(datExpr)

traitData = read.csv("ClinicalTraits.csv");
dim(traitData)
names(traitData)
```

```
allTraits = traitData;
allTraits = allTraits;
dim(allTraits)
names(allTraits)

femaleSamples = rownames(datExpr);
traitRows = match(femaleSamples, allTraits$sample);
datTraits = allTraits[traitRows, -1];
rownames(datTraits) = allTraits[traitRows, 1];

collectGarbage();

#Re-cluster samples
sampleTree2 = hclust(dist(datExpr), method = "average")
traitColors = numbers2colors(datTraits, signed = FALSE);
plotDendroAndColors(sampleTree2, traitColors,
        groupLabels = names(datTraits),
        main = "Sample dendrogram and trait heatmap")

save(datExpr, datTraits, file = "FemaleLiver-01-dataInput.RData")

#Choose a set of soft-thresholding powers
powers = c(c(1:10), seq(from = 12, to=20, by=2))
sft = pickSoftThreshold(datExpr, powerVector = powers, verbose = 5)
sizeGrWindow(9, 5)
par(mfrow = c(1,2));
cex1 = 0.9;
plot(sft$fitIndices[,1], -sign(sft$fitIndices[,3])*sft$fitIndices[,2],
        xlab="Soft Threshold (power)",ylab="Scale Free Topology Model Fit,signed R^2",type="n",
        main = paste("Scale independence"));
text(sft$fitIndices[,1], -sign(sft$fitIndices[,3])*sft$fitIndices[,2],
        labels=powers,cex=cex1,col="red");
abline(h=0.90,col="red")
abline(h=0.80,col="blue")
plot(sft$fitIndices[,1], sft$fitIndices[,5],
        xlab="Soft Threshold (power)",ylab="Mean Connectivity", type="n",
        main = paste("Mean connectivity"))
text(sft$fitIndices[,1], sft$fitIndices[,5], labels=powers, cex=cex1,col="red")

#Module colors
```

```
net = blockwiseModules(datExpr, power = 16, maxBlockSize=5000,
        TOMType = "unsigned", minModuleSize = 30,
        reassignThreshold = 0, mergeCutHeight = 0.25,
        numericLabels = TRUE, pamRespectsDendro = FALSE,
        saveTOMs = TRUE,
        saveTOMFileBase = "femaleMouseTOM",
        verbose = 3)

sizeGrWindow(12, 9)
mergedColors = labels2colors(net$colors)
plotDendroAndColors(net$dendrograms[[1]], mergedColors[net$blockGenes[[1]]],
        "Module colors",
        dendroLabels = FALSE, hang = 0.03,
        addGuide = TRUE, guideHang = 0.05)

moduleLabels = net$colors
moduleColors = labels2colors(net$colors)
MEs = net$MEs;
geneTree = net$dendrograms[[1]];
save(MEs, moduleLabels, moduleColors, geneTree,
        file = "FemaleLiver-02-networkConstruction-auto.RData")

#Table of the correlation between CpG methylation levels and clinical traits, the module colors, and gene information
weight = as.data.frame(datTraits$Fibrosis);
names(weight) = "weight"
modNames = substring(names(MEs), 3)

geneModuleMembership = as.data.frame(cor(datExpr, MEs, use = "p"));
MMPvalue = as.data.frame(corPvalueStudent(as.matrix(geneModuleMembership), nSamples));

names(geneModuleMembership) = paste("MM", modNames, sep="");
names(MMPvalue) = paste("p.MM", modNames, sep="");

geneTraitSignificance = as.data.frame(cor(datExpr, weight, use = "p"));
GSPvalue = as.data.frame(corPvalueStudent(as.matrix(geneTraitSignificance), nSamples));

names(geneTraitSignificance) = paste("GS.", names(weight), sep="");
names(GSPvalue) = paste("p.GS.", names(weight), sep="");

names(datExpr)
```

```
annot = read.csv(file = "GeneAnnotation.csv");
dim(annot)
names(annot)
probes = names(datExpr)
probes2annot = match(probes, annot$gene_id)
sum(is.na(probes2annot))

geneInfo0 = data.frame(substanceBXH = probes,
        geneSymbol = annot$Approved_Symbol_1[probes2annot],
        LocusLinkID = annot$Entrez_Gene_ID[probes2annot],
        moduleColor = moduleColors,
        geneTraitSignificance,
        GSPvalue)
modOrder = order(-abs(cor(MEs, weight, use = "p")));
for (mod in 1:ncol(geneModuleMembership))
{
        oldNames = names(geneInfo0)
        geneInfo0 = data.frame(geneInfo0, geneModuleMembership[, modOrder[mod]],
        MMPvalue[, modOrder[mod]]);
        names(geneInfo0) = c(oldNames, paste("MM.", modNames[modOrder[mod]], sep=""),
        paste("p.MM.", modNames[modOrder[mod]], sep=""))
}
geneOrder = order(geneInfo0$moduleColor, -abs(geneInfo0$GS.weight));
geneInfo = geneInfo0[geneOrder, ]

write.csv(geneInfo, file = "geneInfo.csv")

#A heatmap with module colors and dendrograms
dissTOM = 1-TOMsimilarityFromExpr(datExpr, power =16);
plotTOM = dissTOM^7;
diag(plotTOM) = NA;
sizeGrWindow(9,9)
TOMplot(plotTOM, geneTree, moduleColors, main = "Network heatmap plot, all genes")

#Calculate topological overlap measures
TOM = TOMsimilarityFromExpr(datExpr, power = 16);
annot = read.csv(file = "GeneAnnotation.csv");

modules = c("blue", "grey","turquoise","brown","yellow");

probes = names(datExpr)
```

```
inModule = is.finite(match(moduleColors, modules));
modProbes = probes[inModule];
modGenes = annot$Approved_Symbol[match(modProbes, annot$gene_id)];
modTOM = TOM[inModule, inModule];
dimnames(modTOM) = list(modProbes, modProbes)

val.modTOM1<-as.matrix(modTOM)

write.table(val.modTOM1,"modTOM1.txt",sep="¥t",quote=F)
```

Codes for STAR alignments

```
#Removal of adapter sequences
cutadapt -a AGATCGGAAGAGCACACGTCTGAACTCCAGTCAC -A
AGATCGGAAGAGCGTCGTGTAGGGAAAGAGTGTAGATCTCGGTGGTCGCCGTATCATT -o RNA_R1_trimmed.fastq.gz -
p RNA_R2_trimmed.fastq.gz RNA_R1_001.fastq.gz RNA_R2_001.fastq.gz --minimum-length=20;

#1st RNA alignment
./STAR-master/bin/Linux_x86_64/STAR
        --runThreadN 6
        --readFilesCommand zcat
        --genomeDir ./genomeDir
        --readFilesIn RNA_R1_trimmed.fastq.gz RNA_R2_trimmed.fastq.gz
        --outFileNamePrefix ./RNA;

samtools view -bS RNA.Aligned.out.sam > RNA_aligned.out.bam;
samtools sort RNA_aligned.out.bam RNA_aligned.out_sorted;
samtools index RNA_aligned.out_sorted.bam;

#Generating genome indexes
./STAR-master/bin/Linux_x86_64/STAR
        -runThreadN 6
        --runMode genomeGenerate
        --genomeDir ./genomeDir
        --genomeFastaFiles ./reference/genome.fa
        --sjdbFileChrStartEnd ./SJ.out.tab
        --sjdbOverhang 99;

#2nd RNA alignment
./STAR-master/bin/Linux_x86_64/STAR
        --runThreadN 6
        --readFilesCommand zcat
```

```
    --genomeDir ./genomeDir
    --readFilesIn RNA_R1_trimmed.fastq.gz RNA_R2_trimmed.fastq.gz --outFileNamePrefix ./RNA;

samtools view -bS RNA.Aligned.out.sam > RNA_aligned.out.bam;
samtools sort RNA_aligned.out.bam RNA_aligned.out_sorted;
samtools index RNA_aligned.out_sorted.bam;
```

Codes for HTseq

```
python –m HTSeq.scripts.count –s no -f bam RNA_aligned.out_sorted.bam ./reference/UCSC_hg19_genes.gtf >
RNA_count_gene.txt
```

Codes for DESeq2 (R package)

```
library(DESeq2)

count <- read.csv("RNA_count_gene.csv", sep = ",", header = T, row.names = 1)

count <- as.matrix(count)

dim(count)

group <- data.frame(con =factor(c(rep("G1",29),rep("G3",21))))

dds1 <- DESeqDataSetFromMatrix(countData = count, colData = group, design = ~ con)
dds1 <- estimateSizeFactors(dds1)
dds1 <- estimateDispersions(dds1)
dds1 <- nbinomWaldTest(dds1)
res1 <- results(dds13, contrast = c("con", "G1","G3"))

write.table(res1, file = "2group_result.txt", row.names = T, col.names = T, sep = "¥t")

hoge<-counts(dds1, normalized=T)

write.table(hoge, file = "count_RNA.txt", row.names = T, col.names = T, sep = "¥t")
```

Codes for GSE31803

```
library(affy)
hoge<-ReadAffy()
eset<-mas5(hoge)
dim(exprs(eset))
colnames(exprs(eset))
out_f<-“hoge1.txt”
```

```
write.exprs(eset,file=out_f)
in_f<-"hoge1.txt"
out_f<-"Moylan_RNA_values.txt"
param_M<-40
param_A<-32

data<-read.table(in_f,header=TRUE,row.names=1,sep="¥t",quote="")
data<-log2(data)
colnames(data)<-c(paste("M_",1:40,sep=""),paste("A_",1:32,sep=""))
data.cl<-c(rep(1,param_M),rep(2,param_A))

library(limma)
design<-model.matrix(~as.factor(data.cl))
fit<-lmFit(data,design)
out<-eBayes(fit)
p.value<-out$p.value[,ncol(design)]
q.value<-p.adjust(p.value,method="BH")
ranking<-rank(p.value)

tmp<-cbind(rownames(data),data,p.value,q.value,ranking)
write.table(tmp,out_f,sep="¥t",append=F,quote=F,row.names=F)

topTable(out,coef=colnames(design)[ncol(design)],adjust="BH",number=8)

moy<-topTable(out,coef=colnames(design)[ncol(design)],adjust="BH",number=54675)

out_f2=" moylan_tested.txt"
write.table(moy,out_f2,sep="¥t",append=F,quote=F,row.names=T)
```
